# Risk Factors for Ovarian Cancer: An Umbrella Review of the Literature

**DOI:** 10.3390/cancers14112708

**Published:** 2022-05-30

**Authors:** Eilbhe Whelan, Ilkka Kalliala, Anysia Semertzidou, Olivia Raglan, Sarah Bowden, Konstantinos Kechagias, Georgios Markozannes, Sofia Cividini, Iain McNeish, Julian Marchesi, David MacIntyre, Phillip Bennett, Kostas Tsilidis, Maria Kyrgiou

**Affiliations:** 1 Department of Metabolism, Digestion and Reproduction, Faculty of Medicine, Imperial College London, London W12 0NN, UK; e.whelan@imperial.ac.uk (E.W.); i.kalliala@imperial.ac.uk (I.K.); anysia.semertzidou09@imperial.ac.uk (A.S.); o.raglan@imperial.ac.uk (O.R.); s.bowden@imperial.ac.uk (S.B.); konstantinos.kechagias18@imperial.ac.uk (K.K.); i.mcneish@imperial.ac.uk (I.M.); j.marchesi@imperial.ac.uk (J.M.); d.macintyre@imperial.ac.uk (D.M.); p.bennett@imperial.ac.uk (P.B.); 2 Queen Charlotte’s and Chelsea—Hammersmith Hospital, Imperial College Healthcare NHS Trust, London W12 0HS, UK; 3 Department of Obstetrics and Gynaecology, University of Helsinki and Helsinki University Hospital, FI 00014 Helsinki, Finland; 4 Department of Hygiene and Epidemiology, University of Ioannina School of Medicine, PC45110 Ioannina, Greece; g.markozannes@imperial.ac.uk (G.M.); k.tsilidis@imperial.ac.uk (K.T.); 5 Department of Epidemiology and Biostatistics, School of Public Health, Imperial College London, London SW7 2AZ, UK; 6 Department of Health Data Science, University of Liverpool, Liverpool L69 3GF, UK; sofia.cividini@liverpool.ac.uk; 7 School of Biosciences, Cardiff University, Cardiff CF10 3AX, UK

**Keywords:** ovarian cancer, fallopian tube cancer, gynaecological oncology, hormone replacement therapy, risk factors

## Abstract

**Simple Summary:**

Ovarian cancer is the most lethal cancer of the female genital tract despite major advances in both surgical and oncological treatments. This is in part due to difficulties in identifying those most at risk of developing ovarian cancer, and that there are currently no effective screening strategies. Whilst 20% of cases have a genetic component, the majority have no obvious cause. Many risk factors have been associated with ovarian cancer, although the strength of this evidence remains unclear. This umbrella review attempts to review the validity of associations between non-genetic risk factors and the risk of developing or dying from ovarian cancer. There were six associations that were supported by strong evidence. Greater height, BMI and use of HRT increased the risk, whilst the use of oral contraceptive pill reduced that risk. This review will enable further research into these areas and may promote identification of individuals at high risk.

**Abstract:**

Several non-genetic factors have been associated with ovarian cancer incidence or mortality. To evaluate the strength and validity of the evidence we conducted an umbrella review of the literature that included systematic reviews/meta-analyses that evaluated the link between non-genetic risk factors and ovarian cancer incidence and mortality. We searched PubMed, EMBASE, Cochrane Database of Systematic Reviews and performed a manual screening of references. Evidence was graded into strong, highly suggestive, suggestive or weak based on statistical significance of the random effects summary estimate and the largest study in a meta-analysis, the number of cases, between-study heterogeneity, 95% prediction intervals, small study effects, and presence of excess significance bias. We identified 212 meta-analyses, investigating 55 non-genetic risk factors for ovarian cancer. Risk factors were grouped in eight broad categories: anthropometric indices, dietary intake, physical activity, pre-existing medical conditions, past drug history, biochemical markers, past gynaecological history and smoking. Of the 174 meta-analyses of cohort studies assessing 44 factors, six associations were graded with strong evidence. Greater height (RR per 10 cm 1.16, 95% confidence interval (CI) 1.11–1.20), body mass index (BMI) (RR ≥ 30 kg/m^2^ versus normal 1.27, 95% CI 1.17–1.38) and three exposures of varying preparations and usage related to hormone replacement therapy (HRT) use increased the risk of developing ovarian cancer. Use of oral contraceptive pill reduced the risk (RR 0.74, 95% CI 0.69–0.80). Refining the significance of genuine risk factors for the development of ovarian cancer may potentially increase awareness in women at risk, aid prevention and early detection.

## 1. Introduction

Ovarian cancer is the most lethal gynaecological malignancy in high-income countries [1]. Worldwide, 434,184 new ovarian cancer diagnoses and 293,039 deaths are predicted to occur in 2040, an increase from 295,414 and 184,799, respectively, in 2018 [2]. In the United Kingdom (UK), over 7400 cases are diagnosed every year and 4100 die from the disease [3]. Although recent improvements in treatment with a combination of surgery, chemotherapy and more recently PARP inhibitors [4] have led to an increase in 5-year survival for all stages from 21% in 1970 to 42% in 2017 [3], the overall cure rates and prognosis remain poor [3,5]. Despite ovarian cancer causing only 2.5% of cancers in UK women, it accounts for 5% of all deaths [3]. Poor outcomes have been predominantly related to delays in diagnosis and absence of prevention, as nearly four out of five women are still diagnosed with advanced disease [3,5]. Concerted efforts explored the value of screening with disappointing results [6], whilst to date, the identification of women at high-risk from non-genetic causes, which could enhance prevention and early detection, has not been possible. 

Although approximately 20% of the cases of ovarian cancer have been associated with genetic mutations in Breast Cancer (BRCA)1 and BRCA2 [5], the majority of cases are sporadic. Increased body mass index (BMI) and hormone replacement therapy (HRT) have been proposed as major contributors along with smoking and occupational hazards e.g., asbestos exposure [7]. Conversely, use of the combined contraceptive pill has been suggested to reduce the risk of ovarian cancer [8]. Although many of these suggested associations could be true, some may well be biased. Residual confounding and selective reporting of strong positive results could result in false positive findings and overinflate the observed magnitudes of effect [9,10,11]. Umbrella reviews can systematically appraise the evidence presented in meta-analyses exploring associations between putative risk factors and related outcomes. Recent umbrella reviews have shown that despite claims of significant associations between several risk factors and outcomes, only a fraction were supported by robust evidence without hints of bias [12,13,14,15,16,17,18].

In this study, we present an umbrella review of systematic reviews and meta-analyses of observational studies to systematically appraise the evidence and validity of associations between non-genetic risk factors and the risk of developing or dying from ovarian cancer. 

## 2. Methods

### 2.1. Literature Search

We searched PubMed, EMBASE and the Cochrane Database of Systematic Reviews from inception to September 2019 (more information provided in Supplementary Materials). We also reviewed the citations of the retrieved eligible papers to identify additional publications that may have been overlooked during the preliminary search.

### 2.2. Eligibility Criteria

Systematic reviews or meta-analyses of observational studies in humans that assessed non-genetic risk factors and ovarian cancer incidence and mortality were included. We also included meta-analyses of randomised controlled trials investigating the impact of interventions e.g., dietary, exercise, or mixed interventions.

We excluded meta-analyses that reported benign ovarian pathology as outcome (such as benign ovarian masses), and studies assessing genetic factors as the exposure of interest, as well as studies investigating the prognostic ability of factors on survival of patients previously diagnosed with ovarian cancer [19]. We further excluded any meta-analyses that did not present comprehensive study-specific data (number of cases of ovarian cancer or deaths, number of study population or person years, relative risks with corresponding 95% confidence intervals or standard error) and these missing data were not retrievable from the original studies or after contacting the authors of the meta-analyses. 

In the event of multiple meta-analysis reporting on the same exposure-outcome associations, we selected the one with the largest number of studies to preclude duplication of the original studies. Hand searches were performed to ensure primary studies were not included in more than one systematic review that formed the evidence base. 

### 2.3. Evaluation of the Strength of Evidence

Each identified risk factor and ovarian cancer association was graded into four groups; strong, highly suggestive, suggestive and weak evidence using a series of criteria described previously [16,17,18,20,21,22]. These included: summary random-effects *p*-value; *p*-value of largest study in meta-analysis; number of cases; between-study heterogeneity; 95% prediction interval; small study effects; excess significance bias. Full details of the statistical analysis and grading is presented in the Supplementary Materials.

### 2.4. Evaluation of the Quality of Included Meta-Analyses

The strength and quality of all included meta-analyses was assessed using the AMSTAR 2 tool, which uses 16 measures to appraise the methodological quality of systematic reviews [23] into high, moderate, low or critically low quality (Supplementary Materials).

### 2.5. Data Analysis

Our primary analysis focused on cohort studies as these studies provide most robust evidence among observational studies. We also conducted sensitivity analyses afterwards including case-control studies.

We performed further sensitivity analyses and applied the credibility ceilings threshold to account that a single observational study cannot give more than a maximum certainty, c% (credibility ceiling), that the true effect size is in a different direction from the one suggested by the point estimate [24]. The concordance between the included and duplicate meta-analyses was explored in a sensitivity analysis (Supplementary Materials).

The complete statistical analyses were performed using Stata version 15 (College Station, Texas) (StataCorp 2015) [25], all *p* values were two tailed.

There was no patient involvement.

This project is registered on Open Science Framework (DOI: 10.17605/OSF.IO/YMDSQ). 

## 3. Results

### 3.1. Characteristics of Meta-Analyses

**Figure 1:**
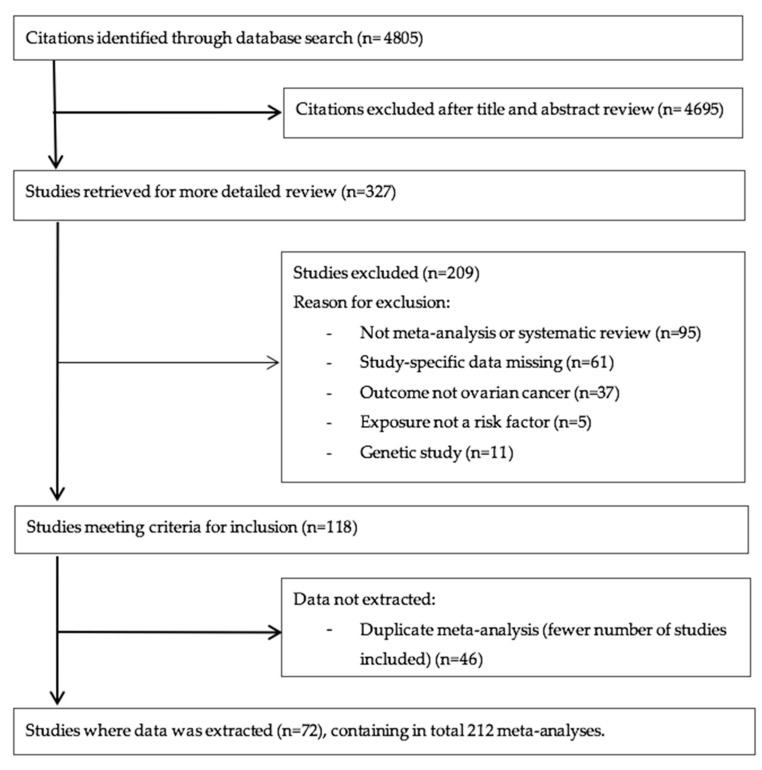
Flow diagram of literature search and study selection.

We identified 72 eligible publications that included 212 meta-analyses of 1853 individual study estimates exploring associations between non-genetic risk factors and the risk of developing or dying from ovarian cancer (Figure 1) [8,19,26,27,28,29,30,31,32,33,34,35,36,37,38,39,40,41,42,43,44,45,46,47,48,49,50,51,52,53,54,55,56,57,58,59,60,61,62,63,64,65,66,67,68,69,70,71,72,73,74,75,76,77,78,79,80,81,82,83,84,85,86,87,88,89,90,91,92,93,94,95,96,97,98]. We identified no meta-analyses of randomized controlled trials investigating the impact of interventions on ovarian cancer incidence and mortality. Out of the 1853 study estimates, 946 (51%) were cohort studies, 795 (43%) case-control studies, 47 (2.5%) were nested case-control, seven (0.4%) were case cohort, one was a pooled analysis of cohort studies and seven (0.4%) were randomised controlled trials. There was a median of six (range: 2 to 40) study estimates combined per meta-analyses. The median (range) number of cases and total population in each meta-analysis was 2873 (20 to 60,524) and 304,057 (16 to 20,307,196), respectively. In 181 out of the 212 included meta-analyses, there were more than 1000 cases of ovarian cancer.

A total of 55 risk factors were studied in the 212 meta-analyses, belonging broadly to eight categories; seven anthropometric indices, 17 dietary factors, two risk factors including physical activity and sedentary behaviour, three risk factors associated with pre-existing medical conditions or interventions, nine factors related to past drug history, four biomarkers, 11 risk factors related to past gynaecological history and three carcinogens. Full details of the risk factors in each category are found in the Supplementary Materials. 

Of the 212 meta-analyses, 174 included at least two cohort studies examining 44 risk factors and were included in the main analysis. Eight of the 174 meta-analyses reported on ovarian cancer incidence and mortality, one meta-analysis reported on ovarian cancer mortality only, and the remaining meta-analysis reported on ovarian cancer incidence.

### 3.2. Summary Effect Size

With *p* < 0.05 as the threshold of statistical significance, the summary fixed and random effects estimates were significant in 39 (22%) and 40 (23%) out of the 174 meta-analyses of cohort studies, respectively (Supplementary Table S3). At *p* < 0.001, 22 (13%) and 17 (10%) meta-analyses produced significant summary results using the fixed and random effects model, respectively. At *p* < 10^−6^, summary fixed effects estimates were significant in 14 (8%) meta-analyses and summary random effects were significant in 10 (6%) meta-analyses. Of the 10 meta-analyses with highly statistically significant summary random effect estimates, eight described an increased risk of ovarian cancer incidence or mortality for the following risk factors: obesity, height, hormone replacement therapy (HRT), endometriosis and asbestos exposure. Two meta-analyses including exposure to the oral contraceptive pill (OCP) and metformin demonstrated a decreased risk of ovarian cancer incidence. 

Assessment of between-study heterogeneity, 95% prediction intervals, small study effects, and excess significance bias were performed only on those meta-analyses that demonstrated statistically significant results i.e., *p* < 0.05, as previously performed by He and colleagues [99]. The meta-analyses with non-significant results are presented in Supplementary Tables S1 and S2. 

The association of the largest study included in each meta-analysis was nominally statistically significant in 20 of the 40 statistically significant meta-analyses (50%), and the relative risks of the largest studies were more conservative than the summary random effects in 28 (70%) meta-analyses.

### 3.3. Heterogeneity between Studies

Seven (18%) of the 40 meta-analyses presented significant evidence of between-study heterogeneity at *p* ≤ 0.10 using the Cochran’s Q test. Large heterogeneity (I^2^ = 50–75%) was found in five (13%) meta-analyses and very large heterogeneity (I^2^ > 75%) in two (5%) meta-analyses for five risk factors including endometriosis, diabetes mellitus, HRT, in vitro fertilisation (IVF) and pelvic inflammatory disease (PID) (Supplementary Table S4).

### 3.4. Small Study Effects

Four meta-analyses (10%) reported small study effects (Egger’s test at *p* < 0.10 with more conservative effects in the largest study of a meta-analysis compared to the summary random effects estimate) for endometriosis, HRT and BMI. (Supplementary Table S4). 

### 3.5. Excess Significance

Five (12.5%) meta-analyses demonstrated evidence of excess significance bias using the largest study estimate as the plausible effect size (*p* < 0.10). These included varying exposures, namely endometriosis, BMI, asbestos exposure, and breastfeeding. Using either the summary fixed or random effects estimates instead of the largest study estimate did not highlight any further meta-analyses with excess significance bias (Supplementary Table S4). 

### 3.6. Grading the Evidence

**Figure 2:**
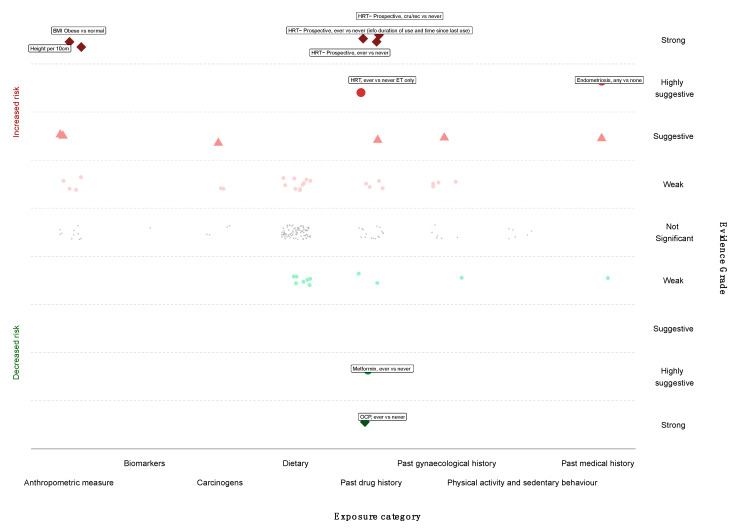
Evidence grading results of the main analysis (cohorts only, random summary effects) displaying the association with increased or decreased risk of ovarian cancer by risk category.

**Figure 3:**
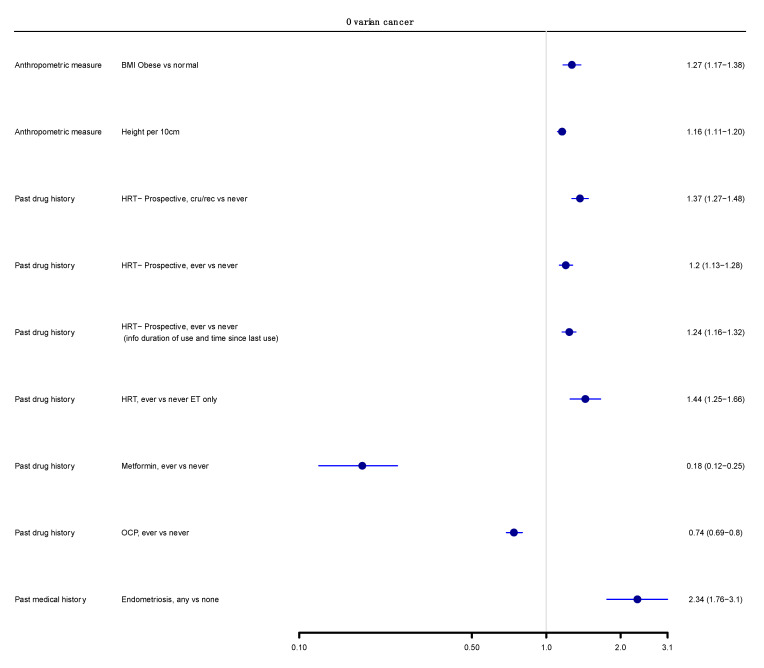
Main analysis results (cohorts only, summary random effects). Forest plot of strong and highly suggestive evidence of an association with ovarian cancer.

We next graded each of the meta-analyses as presenting strong, highly suggestive, suggestive or weak evidence (Table 1 and Table 2) [12,14,100]. Six out of 174 meta-analyses (3%) satisfied the criteria of strong evidence for an association with ovarian cancer incidence; these included prospective studies. Height (RR per 10 cm 1.16; 95% CI 1.11–1.20), BMI (RR ≥ 30 kg/m^2^ vs. <25 kg/m^2^ 1.27; 95% CI 1.17–1.38), and three associations for use of HRT (ever vs. never use—RR 1.20; 95% CI 1.13–1.28; current or recent use vs. never use—RR 1.37, 95% CI 1.27–1.48; ever vs. never use, including information on duration of use—RR 1.25, 95% CI 1.16–1.32) were associated with a higher risk of ovarian cancer. Use of OCP was strongly associated with lower risk of developing ovarian cancer (ever versus never, RR 0.74, 95% CI 0.69–0.80) (Figure 2 and Figure 3).

Only three meta-analyses (3/174, 2%) fulfilled the criteria for highly suggestive evidence. Two of them (2/174, 1%) reported an increased ovarian cancer incidence for use of HRT (ever versus never use, oestrogen therapy only, RR 1.44; 95% CI 1.25–1.66) and endometriosis (any versus none, RR 2.34; 95% CI 1.76–3.10). Metformin use was associated with a lower risk of ovarian cancer incidence (ever versus never, RR 0.18; 95% CI 0.12–0.25) (Figure 2 and Figure 3). There were no exposures that showed a strong or highly suggestive association with an increased risk of ovarian cancer mortality.

Five meta-analyses (5/174, 3%) received a suggestive evidence grade and 26 (26/174, 15%) meta-analyses had weak evidence, whereas the rest (134/174, 77%) had a nominal insignificant association.

### 3.7. Quality Assessment

Thirty-five percent of all the included papers were graded as high (5/72, 7%) or moderate (20/72, 28%) quality, whilst 26% (19/72) and 39% (28/72) were graded as low or critically low (Supplementary Table S5). Papers assessed with ‘low’ or ‘critically low’ quality failed to meet ‘critical’ criteria i.e., lack of protocol, literature search, description of excluded studies and risk of bias assessment. Further details are found in the Supplementary Materials.

Only five included publications [44,58,63,67,101] were considered to be of high quality (Supplementary Table S5), including one of the publications presenting strong evidence on the association between BMI and increased OC incidence [67]. Three publications including the other five meta-analyses presenting strong evidence were deemed to be of low (height and increased OC incidence) [26] or critically low quality (HRT and increased incidence of OC [8] and OCP use and decreased incidence of OC) [34]. Grading of low or critically low quality was due to lack of description for the excluded studies [26] and lack of assessment for publication bias [8,34].

The three meta-analyses that received highly suggestive evidence had high quality (endometriosis) [101], or moderate quality (ever vs. never use of estrogen), only HRT [73] and metformin use [81].

### 3.8. Sensitivity Analyses

None of the associations that received strong evidence grading were downgraded when cohort and case-control studies were both included (Supplementary Table S6). Two statistically non-significant meta-analyses on talcum powder use were upgraded when case-control studies were included as the evidence on the association between talcum powder use and ovarian cancer risk was derived mainly from case-control studies; ever vs. never use demonstrated highly suggestive and long-term (>10 years) vs. no use suggestive evidence. Two statistically non-significant meta-analyses on breastfeeding were also upgraded when case-control studies were included; ever versus never use to highly suggestive and breastfeeding >12 months versus never to weak evidence. After the inclusion of case-control studies, the association of parity with ovarian cancer risk was supported with weak evidence although this was previously insignificant.

All six of the risk factors that attained strong evidence (height per 10 cm, BMI ≥ 30 kg/m^2^ versus normal, all three HRT risk factors and OCP use) remained nominally statistically significant in the main analysis until a 15% credibility ceiling was applied. The three risk factors found to have highly suggestive evidence remained nominally statistically significant until a 10% credibility ceiling was applied (Supplementary Table S7).

We identified that 33 associations had more than one published meta-analysis assessing the same exposure and outcome combination, frequently with several meta-analyses examining different exposure contrasts of the same risk factor (*n* = 41). For most of the duplicate meta-analyses (*n* = 38/41 93%) there was concordance in direction, magnitude and statistical significance of the summary associations between them (Supplementary Table S8).

## 4. Discussion

### 4.1. Main Findings and Interpretation

This umbrella review explored the associations of 55 non-genetic risk factors with the risk of developing or dying from ovarian cancer and included 212 meta-analyses. Only six associations were graded with strong evidence. Greater height and BMI and the use of HRT were associated with a higher risk of developing ovarian cancer whilst the use of OCP was associated with lower disease risk. Further positive associations between HRT use as well as endometriosis received a highly suggestive evidence grade, whilst metformin use had highly suggestive evidence for lower disease risk.

Our finding for height as a risk factor for ovarian cancer incidence is in agreement with the WCRF CUP report, which judged the evidence as ‘convincingly strong’ [80]. Two mechanisms have been postulated: increased total number of stem cells with subsequent increased chance of DNA mutations during cell division [102] and/or raised insulin-like growth factor 1 (IGF1) (major determinant of height) and its link to a number of solid tumours including ovarian tumours [102,103]. However, a recent Mendelian randomisation study showed evidence for an association of height only with clear cell carcinoma (OR 1.36; 95% CI 1.15–1.61) but not with other subtypes [104]. Most observational studies have not performed analyses by histological subtypes of ovarian cancer; thus, we were unable to assess these associations in the current umbrella review. Future large epidemiological studies or consortia thereof should address potential differential associations by ovarian cancer subtypes.

The strong evidence on the association of obesity with ovarian cancer risk is also in line with previously published umbrella reviews [16,17] and the World Health Organisation (WHO)/International Agency for Research on Cancer (IARC) World Cancer Report 2020 [105]. This was also supported by a recent Mendelian study, which suggested evidence of a link between obesity and invasive epithelial ovarian cancer [104]. This study also presented evidence suggesting that BMI is differentially associated by histological subtypes of ovarian cancer. Positive associations of BMI with high grade serous and endometrioid carcinomas were reported, but associations for low grade serous, mucinous and clear cell carcinomas had wide confidence intervals including the null value. A pooled analysis of observational studies in the Ovarian Cancer Association Consortium reported an increased risk of high BMI with total ovarian cancer, which was more pronounced for borderline serous, invasive endometrioid and invasive mucinous tumours [106]. Whilst our analysis examined the association between BMI and ovarian cancer throughout different life stages i.e., in young adulthood, pre- and post-menopause, the included studies did not contain subanalyses by histology. Several pathways have been proposed linking obesity with cancer including the IGF-axis signalling and hyperinsulinaemia, alterations in sex hormone metabolism, and subclinical chronic low-grade inflammation with alterations in adipokine pathophysiology [107,108]. Both insulin and leptin have been shown to promote the growth of cancer cells, whilst leptin also functions as an inflammatory cytokine. 

A number of hormonal and reproductive factors have been previously associated with ovarian cancer. Our findings confirm strong evidence to support higher risk with HRT and lower risk with OCP use. An analysis according to the length of OCP use was not possible. Breastfeeding [53] reported highly suggestive evidence for lower risk, whilst nulliparity [61] showed weak evidence of a higher risk when both cohort and case-control studies were included. 

The impact of HRT use was confirmed in all nine meta-analyses using different HRT formulations (e.g., oestrogen alone, oestrogen and progesterone continuously, oestrogen and progesterone sequentially) and in the WHO/IARC World Cancer Report (WCR) 2020 [105]. No Mendelian randomisation studies on HRT use exist due to the lack of robust genetic variants available to serve as proxies. The WHO/IARC WCR 2020 also agreed with our analysis for an inverse association for OCP use (RR 0.74, 95% CI 0.69–0.80) [105].

The mechanisms that underpin these associations are complex. The concept of the ‘incessant ovulation’ supports the notion that recurrent minor trauma caused to the ovarian epithelial surface as a result of ovulation increases the risk of malignant transformation. This process may explain the reduction in risk with OCP, breastfeeding and parity as they are associated with fewer ovulatory cycles [109,110,111,112,113]. Furthermore, it has been suggested that prolonging the ovarian epithelial surface proliferation stimulated by oestrogen exposure e.g., in HRT could result in ovarian oncogenesis. Indeed, 60% of ovarian tumours are oestrogen-receptor positive [114]. Several mechanisms have been suggested linking oestrogens with ovarian cancer. These include (i) a direct transcriptional effect of oestrogens on target genes via activation of the oestrogen receptor-α leading to the transcription of several genes which stimulate cell proliferation and thus an increased risk of mutations, (ii) promotion of tumour progression through influencing of signalling pathways leading to the activation of epidermal growth factor receptor, and (iii) generation of free radicals from the metabolic activation of catechol oestrogens causing neoplastic transformation of cells [115]. Conversely, progesterone exposure may counteract these effects. Progesterone receptors are also present in normal ovarian epithelium and it has been suggested that progestins may counteract the proliferative effect of oestrogen by prompting apoptosis within the ovary [116]. This plausible explanation has been reflected both in our study where oestrogen-only HRT was supported by highly suggestive evidence, whilst mixed preparations demonstrated weak evidence, and also in observational studies where oestrogen plus progestin HRT preparations display a slightly weaker effect than oestrogen-only preparations [117,118]. OCP studies have also consistently demonstrated a reduction in ovarian cancer risk with cyclical administration of progestins [110,119,120,121].

Several advances have been made since the theory of ‘incessant ovulation’ as a causative factor in ovarian cancer was first postulated in the 1970′s. One such concept includes that of extra-ovarian origins of ovarian cancer, the most common of which is that of fallopian tube cancers, considered to be the origin of many ovarian high grade serous ovarian cancers (HGSOC), along with serous tubal intraepithelial carcinoma (STIC) lesions found in fallopian tubes removed prophylactically in BRCA-positive patients. The pro-inflammatory environment of repeated ovulation has also been demonstrated to affect the fallopian tubes, with the resulting pro-repair environment providing tumorigenic factors which potentially supports malignant transformation. Other extra-ovarian sites include mucinous tumours originating from the gastrointestinal tract or cervix, and clear cell and endometrioid ovarian cancers being linked with endometriosis [122,123,124].

The positive association of endometriosis with ovarian cancer was supported by highly suggestive evidence when using only cohort studies, and it was upgraded to strong evidence when case-control studies were included. Endometriosis has been primarily associated with clear cell (OR 3.05, 95% CI 2.43–3.84) [125] and endometrioid (OR 2.04, 95% CI 1.67–2.48) [125] ovarian cancer in the published literature, although the studies included in our analysis did not provide data by histological subtype. The Mendelian randomization study by Yarmolisky et al. confirmed these findings (invasive epithelial ovarian cancer, OR 1.10, 95% CI 1.06–1.15; endometrioid, OR 1.14, 95% CI 1.04–1.24; clear cell, OR 1.49, 95% CI 1.29–1.73) [104]. Several mechanistic theories have been postulated linking endometriosis to these endometriosis-associated ovarian cancers (EAOC). Loss of tumour suppressor genes *PTEN* and *ARID1A*, caused by oxidative stress from iron-rich endometriotic cysts have been found in both EAOC tumours and adjacent endometriotic tissue [126]. ‘Retrograde menstruation’ has also been extensively discussed whereby endometrial cells transplanted to the pelvic peritoneum and ovaries undergo neoplastic change as a result of inflammatory processes leading initially to atypical endometriosis and subsequently neoplastic change [127]. This concept is further supported by our finding of suggestive evidence that tubal ligation reduces the risk of ovarian cancer (OR 0.70, 95% CI 0.60–0.81).

Our umbrella review revealed suggestive evidence that diabetes increases the risk of ovarian cancer incidence [89], which is in line with a previously published assessment of the literature by Tsilidis and colleagues (RR 1.17, 95% CI 1.02–1.34) [14]. Furthermore, ever use of metformin was found to have highly suggestive evidence for a lower ovarian cancer risk (RR 0.18, 95% CI 0.12–0.25) [81]. Several mechanisms linking diabetes and oncogenesis have been described. It has been suggested that insulin resistance could induce the expression of cytokines which have tumour-promoting effects, elevated blood glucose is associated with increased vascular endothelial growth factor thus promoting angiogenesis and aiding tumour proliferation [128] and hyperinsulinaemia could increase the levels of bioavailable oestrogen through a reduction in sex hormone binding globulin (SHBG) levels. This apparent inconsistency in grading between diabetes and use of metformin may be due to the inclusion under diabetes of people with type 1 and type 2 diabetes and gestational diabetes, with possibly only a subset of these patients using metformin. Furthermore, metformin may also be used for other indications e.g., polycystic ovarian syndrome. 

There has been intense debate as to whether talcum powder use increases the risk of ovarian cancer [129]. The multinational cosmetics company Johnson & Johnson are currently appealing a $4.69 bn verdict against it from July 2018 in Missouri, United States of America (USA), towards 22 plaintiffs who claim that the use of their talcum powder led to ovarian cancer due to contamination with asbestos and heavy metals. A further ruling in April 2020 in New Jersey, USA has dictated that a new lawsuit against the company for the same claim may proceed after an appeal against inclusion of certain expert testimonies. The main body of evidence derives from case-control studies. The meta-analysis comparing ever vs. never use included 27 studies, of which only three were cohort studies. Although none of the cohort studies reported significant results, the evidence grading was highly suggestive when case-control studies were included and this would have been graded as strong if the 95% PI did not just include unity. Case-control studies are susceptible to recall bias, which has driven our main analysis to include cohort studies only. Therefore, we do not consider the evidence of this association as robust, which is supported by a recent pooled analysis of four prospective cohort studies showing no association [130].

### 4.2. Strengths and Limitations

In this umbrella review, we have presented the most extensive critical appraisal of published associations to date. A wide array of statistical tests and sensitivity analysis were used to assess evidence strength and validity in an attempt to create a systematic and transparent method of evidence appraisal of the literature.

Several limitations and caveats should also be considered when interpreting our findings. Although some individual studies may have been missed in the literature search of the original meta-analyses, this is not expected to have altered our inference as duplicate meta-analyses on the same exposure-outcome pairs gave similar results. The quality of the original studies included in the meta-analyses was also not assessed and was beyond the scope of this umbrella review, but we did assess the quality of the meta-analyses. Furthermore, the statistical tests used can ‘hint’ the presence of possible bias but are unable to confirm its definitive presence and source. The analysis was further restricted by the inability to explore the impact of risk factors separately on pre- and post-menopausal women, for different histological subtypes (e.g., serous or endometrioid) [8], the preparation and duration of use of HRT or OCP or the impact of treatment, e.g., metformin, on DM or the interval over which weight is gained due to the lack of study-specific data provided by the published meta-analyses. Although BMI, weight and weight gain as well as diabetes and metformin use are closely related, the evidence for each was assessed independently.

## 5. Conclusions

The positive associations of height, obesity and use of HRT with ovarian cancer risk, and the inverse association of OCP use with disease risk were supported by strong evidence. There was further highly suggestive evidence that endometriosis increases disease risk, whilst metformin reduces it. Although further exposures may be also associated with ovarian cancer, the evidence is less certain and further research is required.

The description of potentially genuine risk factors found to alter ovarian cancer incidence and mortality is of great interest to the scientific community, clinicians and the public given the dearth of preventative strategies for ovarian cancer. Information on hormonal modifiable risk factors such as HRT and OCP use could be used in the counselling of women considering their use and how this may theoretically positively or inversely affect their risk, whilst further research on the mechanisms that promote reduction in risk in exposures such as the oral contraceptive pill is required. Drugs such as metformin or others working on similar metabolic pathways may be further used and are possible chemopreventative targets. The development of successful methods of screening for ovarian cancer is an ongoing effort and effective preventative measures are yet to be discovered. Whilst the use of risk factors to determine level of risk clinically may be challenging, their recognition is a useful and necessary contribution to the continuing exploration of the non-genetic causes associated with ovarian cancer. Whilst acknowledging that further research is required, in the absence of efficient screening and prevention, evidence on the strength of previously proposed associations may enhance awareness of individuals at higher risk of ovarian cancer and promote prevention or early detection.

## Figures and Tables

**Table 1:** Summary of evidence grading for meta-analysis of risk factors associated with ovarian cancer incidence or mortality–cohort studies only *.

Evidence	Criteria Used	Decreased Risk	Increased Risk
**Strong**	*p* < 10^−6 ||^; >1000 cases; I^2^ < 50%; no small study effects ^¶^; prediction interval excludes the null value; no excess significance bias ^†^ *n* = 6	***Past drug history*** OCP inc Ever vs. never **	***Anthropometric measure*** Height inc Per 10 cm BMI; ≥30 kg/m^2^ vs. normal
			***Past drug history*** HRT inc Ever vs. never (prospective studies) HRT inc Current/recent vs. never (prospective studies) HRT inc Ever vs. never (prospective studies; info duration of use and time since last use known)
**Highly Suggestive**	*p* < 10^−6 ||^; >1000 cases; *p* < 0.05 of the largest study in a meta-analysis *n* = 3	***Past drug history*** Metformin inc Ever vs. never	***Past drug history*** HRT inc Ever vs. never ET only
			***Medical history*** Endometriosis inc Any vs. none
**Suggestive**	*p* < 10^−3 ||^; >1000 cases *n* = 5	***None***	***Anthropometric measures*** BMI inc iya per 5 kg/m^2^ increase BMI inc per 5 kg/m^2^ increase
			***Asbestos*** Any vs. none MO
			***Medical history*** Diabetes inc; Yes vs. no
			***Past drug history*** HRT inc; Current vs. ever
**Weak**	*p* < 0.05 *n* = 26	**Reproductive factors** Breastfeeding inc; Per 5 mo increase in duration	**Anthropometric measures** BMI inc PrMP Obese vs. normal BMI inc PoMP; Obese vs. normal Per 5 kg weight inc WG per 5 kg increase PoMP, HRT, inc
		**Past drug history** NSAIDS inc, Non aspirin; Ever vs. never OCP inc; Ever vs. never	
		**Medical history** SLE inc; observed vs. expected	**Asbestos** Total exposed vs. non-exposed, MO High exposed vs. non-exposed, MO
		**Dietary Intake** Tea (black) inc; Highest vs. lowest Non herbal tea inc; Highest vs. lowest Calcium inc; Highest vs. lowest Non-starchy vegetables inc; Per 100 g/day	
			**Dietary intake** Dairy total inc; Highest vs. lowest Dairy skim/low fat inc; Highest vs. lowest Dairy lactose inc; Highest vs. lowest Meat (processed) inc; Highest vs. lowest Meat (red and processed) MO; Per 100 g/week increment
			**Past drug history** HRT inc; Ever vs. never (continuous E + P) HRT inc; Ever vs. never (sequential E + P) HRT inc; Ever vs. never (E + P) HRT inc; Ever vs. never (E + E/P)
			**Reproductive Factors** PID inc; Ever vs never IVF inc; Ever vs. never (reference group general population) IVF inc; Ever vs. never (reference group IVF population)

(1) Abbreviations: BMI, body mass index; BMI iya, body mass index in young adulthood; BMI PrMP, body mass index premenopausal; CC, case control; HRT, hormone replacement therapy; inc, incidence; MO, mortality; NSAID, non-steroidal anti-inflammatory drugs; WG, weight gain; BMI PoMP, body mass index postmenopausal; CRP, c-reactive protein; OCP, oral contraceptive pill; SLE, systemic lupus erythematous; E + P, estrogen and progesterone; E + E/P, estrogen and estrogen/progesterone; PID, pelvic inflammatory disease; IVF, in-vitro fertilization; RR, relative risk. (2) Key: * only meta-analyses meeting at least weak grade of evidence listed. ** % reduction in the standard error. ^||^ *p* indicates the *p*-values of the meta-analysis random effects model. ^¶^ Small study effect is based on the *p*-value from the Egger’s regression asymmetry test (*p* > 0.1) where the random effects summary estimate was larger compared to the point estimate of the largest study in a meta-analysis. ^†^ Based on the *p*-value (*p* > 0.1) of the excess significance test using the largest study (smallest standard error) in a meta-analysis as the plausible effect size.

**Table 2:** Details of evidence grading for meta-analysis of risk factors for ovarian cancer incidence or mortality—only cohort studies included *.

Exposure	Exposure Contrast	*N ˆ*	Sample Size Cases/Cohort	Largest Study ^#^	Random Effects Summary RR (95% CI) ^¥^	Random *p*-Value ^||^	95% Prediction Interval	Egger’s *p* ^∞^	I^2^ (%)	Excess Significance ^§^		Evidence Grading **^,†,¶^
										O/E ^α^	*p*-Value ^φ^	
**Strong evidence**												
**Anthropometric measure**												
**Height**	**Per 10 cm**	**16**	**18663/13600000**	**1.14 (1.10–1.18)**	**1.16 (1.11–1.20)**	**2.2 × 10^−13^**	**1.06–1.26**	0.18	27	9/6.22	0.15	Strong
BMI	Obese vs. normal	13	6947/20560388 ^τ^	1.27 (1.19–1.36)	1.27 (1.17–1.38)	2.6 × 10^−8^	1.09–1.47	0.88	12	3/5.30	NP	Strong
**Past drug history**												
HRT-Prospective	Current/recent vs. never	12	11664/948390	1.28 (1.14–1.44)	1.37 (1.27–1.48)	1.3 × 10^−15^	1.26–1.50	0.68	0	3/7.20	NP	Strong
HRT-Prospective	Ever vs. never	17	12110/950663	1.15 (1.06–1.26)	1.20 (1.13–1.28)	2.1 × 10^−9^	1.13–1.28	0.71	0	4/4.90	NP	Strong
HRT-Prospective	Ever vs. never (info duration of use and time since last use)	14	11866/949657	1.16 (1.05–1.28)	1.24 (1.16–1.32)	6.0 × 10^−10^	1.15–1.33	0.97	0	2/4.87	NP	Strong
OCP	Ever vs. never	45	7726/32201	0.74 (0.67–0.82)	0.74 (0.69–0.80)	5.8 × 10^−16^	0.68–0.81	0.61	0	3/6.68	NP	Strong
**Highly suggestive evidence**												
**Past drug history**												
HRT	Ever vs. never ET only	11	7512/2302683	1.31 (1.11–1.54)	1.44 (1.25–1.66)	7.1 × 10^−7^	0.99–2.09	0.71	48	6/7.01	NP	Highly suggestive
Metformin	Ever vs. never	3	3288/513702	0.16 (0.14–0.17)	0.18 (0.12–0.25)	2.5 × 10^−23^	0.01–4.31	0.38	14	2/2.42	NP	Highly suggestive
**Anthropometric measure**												
Height	per 5 cm increase	13	16198/3514114	1.07 (1.05–1.09)	1.07 (1.05–1.10)	1.2 × 10^−9^	1.02–1.14	0.31	32	8/2.77	<0.01	Highly suggestive
**Medical history**												
Metformin	Ever vs. never	3	3288/513702	0.16 (0.14–0.17)	0.18 (0.12–0.25)	2.5 × 10^−23^	0.01–4.31	0.38	14	2/2.42	NP	Highly suggestive
**Suggestive evidence**												
**Environmental factors**												
Asbestos	Any vs. none	14	5165/906145	1.30 (0.90–1.80)	1.86 (1.46–2.36)	5.0 × 10^−7^	1.05–3.29	0.64	28	4/1.87	0.10	Suggestive
**Anthropometric measures**												
BMI	per 5 kg/m^2^ increase	24	17734/16300000	0.97 (0.93–1.01)	1.07 (1.04–1.11)	<0.01	0.96–1.21	0.07	48	6/1.80	<0.01	Suggestive
BMI	iya per 5 kg/m^2^ increase	6	9452/11100000	1.16 (1.04–1.29)	1.12 (1.05–1.19)	<0.01	1.03–1.23	0.60	0	0/1.00	NP	Suggestive
**Medical history**												
Diabetes Mellitus	DM vs. no DM	17	5036/2868215	1.23 (1.15–1.32)	1.32 (1.14–1.52)	<0.01	0.81–2.15	0.30	80	6/6.71	NP	Suggestive
**Past drug history**												
HRT	Current vs. ever	5	3958/1342899	1.20 (1.09–1.32)	1.28 (1.15–1.42)	6.5 × 10^−6^	1.01–1.62	0.08	14	3/2.91	0.93	Suggestive
**Weak evidence**												
**Asbestos**												
Asbestos	Total exp vs. nonexp	20	126/21973	1.12 (0.66–1.80)	1.77 (1.37–2.27)	9.7 × 10^−6^	0.85–3.66	0.72	35	6/1.06	<0.01	Weak
Asbestos	High exp vs. nonexp	6	20/6149	1.10 (0.37–2.21)	2.78 (1.36–5.66)	0.01	0.42–18.44	0.78	45	2/0.31	<0.01	Weak
**Anthropometric measure**												
BMI	PrMP Obese vs. normal	3	71/350211	1.56 (1.14–2.16)	1.57 (1.20–2.06)	<0.01	0.27–9.02	0.61	0	1/0.66	0.64	Weak
BMI	PoMP Obese vs. normal	5	350/546195	1.02 (0.82–1.26)	1.23 (1.03–1.47)	2.6 × 10^−1^	0.72–2.09	0.52	46	1/ 0.25	0.13	Weak
Weight	Per 5 kg weight	4	1006/297350	1.02 (1.00–1.05)	1.03 (1.01–1.05)	<0.01	0.98–1.08	0.42	7	1/0.21	0.08	Weak
Weight gain	per 5 kg increase PoMP, HRT	2	217/23984	1.16 (1.03–1.31)	1.13 (1.03–1.24)	0.01	NA	NA	NA	1/0.27	0.13	Weak
**Dietary intake**												
Dairy, total products	Highest vs. lowest	2	427/90001	1.61 (1.07–2.42)	1.66 (1.19–2.31)	<0.01	NA	NA	NA	1/1.87	NP	Weak
Dairy, skim/low fat	Highest vs. lowest	3	728/170327	1.32 (0.97–1.82)	1.35 (1.09–1.68)	<0.01	0.35–5.43	0.21	0	0/1.93	NP	Weak
Dairy, lactose	Highest vs. lowest	3	728/170327	1.48 (1.05–2.09)	1.47 (1.17–1.84)	<0.01	0.34–6.29	0.42	0	1/2.64	NP	Weak
Meat; processed	Highest vs. lowest	3	1018/696100	1.23 (0.92–1.63)	1.26 (1.02–1.56)	3.5 × 10^−2^	0.31–5.07	0.37	0	0/1.56	NP	Weak
Meat; red and processed	Per 100 g/week increment	21	6536/2140286	1.02 (0.98–1.06)	1.01 (1.00–1.04)	3.4 × 10^−2^	1.00–1.04	0.12	0	0/1.14	NP	Weak
Non starchy vegetables	Per 100 g/day	6	2053/641079	1.00 (0.93–1.07)	0.94 (0.89–1.00)	4.0 × 10^−2^	0.82–1.08	0.21	28	1/0.30	0.19	Weak
Tea; black	Highest vs. lowest	5	1299/203998	0.63 (0.40–0.99)	0.73 (0.56–0.93)	1.2 × 10^−2^	0.42–1.24	0.44	15	2/4.71	NP	Weak
Calcium	Highest vs. lowest	5	1726/351192	0.86 (0.68–1.10)	0.86 (0.74–1.00)	4.0 × 10^−2^	0.67–1.09	0.62	0	0/1.66	NP	Weak
Non herbal tea	Highest vs. lowest	3	734/164882	0.63 (0.40–0.99)	0.69 (0.52–0.93)	1.4 × 10^−2^	0.11–4.57	0.03	0	1/2.74	NP	Weak
**Past drug history**												
HRT	Ever vs. never (continuous E + P)	4	3337/1265735	1.13 (0.96–1.34)	1.22 (1.06–1.40)	<0.01	0.90–1.65	0.07	0	1/1.63	NP	Weak
HRT	Ever vs. never (sequential E + P)	4	3337/1265736	1.14 (0.98–1.32)	1.35 (1.06–1.72)	1.5 × 10^−2^	0.54–3.35	0.18	50	2/1.75	0.80	Weak
HRT	Ever vs. never (ET + PT)	9	7512/2302683	1.50 (1.34–1.68)	1.23 (1.08–1.14)	2.3 × 10^−3^	0.87–1.75	0.40	53	3/8.65	NP	Weak
HRT	Ever vs. never (ET+ E/PT)	2	543/141880	1.50 (0.92–2.44)	1.55 (1.05–2.30)	2.7 × 10^−2^	NA	NA	NA	0/1.89	NP	Weak
NSAIDS Non aspirin	Ever vs. never	6	1782/505136	0.90 (0.75–1.08)	0.90 (0.81–1.00)	4.4 × 10^−2^	0.78–1.04	0.55	0	0/1.02	NP	Weak
OCP	Ever vs. never	3	60/80670	0.60 (0.30–1.40)	0.43 (0.25–0.75)	3.0 × 10^−3^	0.01–15.16	0.41	NA	1/0.73	0.71	Weak
**Reproductive factors**												
PID	Ever vs. never	6	8285/2929284	1.05 (0.92–1.20)	1.32 (1.05–1.66)	1.6 × 10^−2^	0.71–2.47	0.35	65	2/1.01	0.28	Weak
IVF	Ever vs. never (reference group general population excluding OC diagnosis < 1yr post treatment)	6	31606/1438001	1.30 (0.90–1.88)	1.47 (1.06–2.03)	2.0 × 10^−2^	0.73–2.96	0.64	23	1/1.55	NP	Weak
IVF	Ever vs. never reference group IVF population; total follow up	6	31606/1438002	1.35 (0.93–1.96)	1.66 (1.08–2.55)	2.2 × 10^−2^	0.52–5.28	0.91	52	2/1.64	0.74	Weak
Breastfeeding	Per 5 mo increase in duration	3	1180/447386	0.98 (0.92–1.05)	0.94 (0.89–1.00)	0.03	0.80–1.49	0.59	22	1/0.17	0.04	Weak
**Medical history**												
SLE	Observed vs. expected	4	44/40855	0.82 (0.54–1.20)	0.73 (0.53–1.00)	4.9 × 10^−2^	0.36–1.46	0.97	0	0/0.26	NP	Weak

(1) Abbreviations: BMI, body mass index; BMI iya, body mass index in young adulthood; BMI PoMP, body mass index postmenopausal; BMI PrMP, body mass index premenopausal; CC, case control; CRP, c-reactive protein; E + P, estrogen and progesterone; E + E/P, estrogen and estrogen/progesterone; HRT, hormone replacement therapy; IVF, in-vitro fertilization; NA, not available; np; not pertinent, because the estimated is larger than the observed, and there is no evidence of excess statistical significance based on the assumption made for the plausible effect size; NSAID, non- steroidal anti -inflammatory drugs; OC, ovarian cancer; OCP, oral contraceptive pill; PID, pelvic inflammatory disease; RR, relative risk; SLE, systemic lupus erythematous; WG, weight gain; (2) Key: * only meta-analyses meeting at least weak grade of evidence listed. ˆ Number of studies. ^#^ Relative risk and 95% confidence interval of largest study (smallest standard error) in each meta-analysis. ^¥^ Random effects refer to summary risk ratio (95% confidence interval) using the random-effects model. ^||^ *p*-value of summary random effects estimate. ^∞^ *p*-value from the Egger’s regression asymmetry test. ^§^ Expected number of statistically significant studies using the point estimate of the largest study (smallest standard error) as the plausible effect size. ^α^ Observed/Expected number of statistically significant studies. **^φ^** *p* value of the excess statistical significance test. All statistical tests were two-sided. ^¶^ Small study effect is based on the *p*-value from the Egger’s regression asymmetry test (*p* > 0.1) where the random effects summary estimate was larger compared to the point estimate of the largest study in a meta-analysis. ^†^ Based on the *p*-value (*p* > 0.1) of the excess significance test using the largest study (smallest standard error) in a meta-analysis as the plausible effect size. ^τ^ Person years; (3) ** Summary of evidence grading criteria: (i) Weak: *p* < 0.05 ^||^; (ii) Suggestive: *p* < 10^−3^ **^||^**; >1000 cases; (iii) Highly suggestive: *p* < 10^−6^ **^||^**; >1000 cases; *p* < 0.05 of the largest study in a meta-analysis; (iv) Strong: *p* < 10^−6^ **^||^**; >1000 cases; *p* < 0.05 of the largest study in a meta-analysis; I^2^ < 50%; no small study effect ^¶^; prediction interval excludes the null value; no excess significance bias ^†^.

## Supplementary Materials

The following supporting information can be downloaded at https://www.mdpi.com/article/10.3390/cancers14112708/s1, Appendix S1: Search strategy; Appendix S2: Protocol; Appendix S3: Supplementary methods and results; Table S1: Excluded duplicate studies; Table S2: Cohort studies, not statistically significant; Table S3: All studies, not statistically significant; Table S4: Description of cohort studies, statistically significant; Table S5: Evaluation of heterogeneity, small study effects and excess significance bias, statistically significant cohort studies; Table S6: AMSTAR 2; Table S7: Evidence grading, all statistically significant studies; Table S8: Sensitivity analysis using credibility ceilings, cohort studies; Table S9: Evaluation of heterogeneity, small study effects and excess significant bias, all studies; File S1: PRISMA Checklist.
