# Supplementary material for: Risk Factors for Ovarian Cancer: An Umbrella Review of the Literature
Source: Cancers (Basel). 2022 May 30;14(11):2708. doi: 10.3390/cancers14112708 (PMC9179274; doi:10.3390/cancers14112708)
Supplement: Supplementary file 1 [file cancers-14-02708-s001.zip › Appendix S1.pdf]

Search Strategy

Search strategy used in the literature review for PubMed and Embase databases and Cochrane Library of Systematic Reviews (search limited to humans, conducted 17th September 2019). Two investigators (EW and OR) independently performed the literature search and assessed eligibility of retrieved papers whilst any disagreements were resolved through discussion with a third investigator (IK).

PubMed and Embase	
1. Exposure terms	Hip circumference OR waist circumference OR waist to hip ratio OR weight OR height OR weight gain OR obesity OR obese OR BMI OR Body Mass index OR bariatric surgery OR insulin OR metformin OR antiglycaemics OR aspirin OR aspirin containing medication OR paracetamol OR painkillers OR Diabetes OR type 2 diabetes mellitus OR birth weight OR exercise OR physical activity OR radiation OR radiotherapy OR hormone replacement therapy OR HRT OR HPV OR human papillomavirus OR breast cancer OR cancer OR tamoxifen OR smoking OR smoker OR tobacco OR contraception OR OCP or oral contraceptives OR oral contraceptive pills OR ethnic origin OR ethnicity OR race OR PCOS OR polycystic ovarian syndrome OR family history OR familial OR menarche OR menopause OR premenopausal OR post menopausal OR parity OR pregnancy OR alcohol OR coffee OR caffeinated intake OR caffeine intake OR caffeine OR decaffeinated OR decaffeinated intake OR marital status OR married OR single OR divorced OR cohabiting OR living together OR partner OR partnered OR breastfeeding OR endometrial hyperplasia OR socioeconomic status OR poverty OR wealth OR education OR level of education OR educational level OR schooling OR past gynaecological history OR endometriosis OR fibroid* OR non-cancerous cyst* OR pelvic inflammatory disease OR PID
	AND
2. Primary outcome terms	ovarian cancer OR ovarian carcinoma OR ovarian neoplasm OR ovarian tumor* OR ovarian tumour* OR adenocarcinoma OR cystadenoma* OR cystadenocarcinoma* OR adenoma* OR endometrioid carcinoma* OR ovarian maligna* OR borderline
	AND
3. Review terms	systematic review OR meta-analysis OR metaanalysis
Cochrane Database of Systematic reviews	
1. Exposure terms	Hip circumference OR waist circumference OR waist to hip ratio OR weight OR height OR weight gain OR obesity OR obese OR BMI OR Body Mass index OR bariatric surgery OR insulin OR metformin OR antiglycaemics OR aspirin OR aspirin containing medication OR

	paracetamol OR painkillers OR Diabetes OR type 2 diabetes mellitus OR birth weight OR exercise OR physical activity OR radiation OR radiotherapy OR hormone replacement therapy OR HRT OR HPV OR human papillomavirus OR breast cancer OR cancer OR tamoxifen OR smoking OR smoker OR tobacco OR contraception OR OCP or oral contraceptives OR oral contraceptive pills OR ethinic origin OR ethnicity OR race OR PCOS OR polycystic ovarian syndrome OR family history OR familial OR menarche OR menopause OR premenopausal OR post menopausal OR parity OR pregnancy OR alcohol OR coffee OR caffeinated intake OR caffeine intake OR caffeine OR decaffeinated OR decaffeinated intake OR marital status OR married OR single OR divorced OR cohabiting OR living together OR partner OR partnered OR breastfeeding OR endometrial hyperplasia OR socioeconomic status OR poverty OR wealth OR education OR level of education OR educational level OR schooling OR past gynaecological history OR endometriosis OR fibroid* OR non-cancerous cyst* OR pelvic inflammatory disease OR PID
	AND
2. Primary outcome terms	ovarian cancer OR ovarian carcinoma OR ovarian neoplasm OR ovarian tumor* OR ovarian tumour* OR adenocarcinoma OR cystadenoma* OR cystadenocarcinoma* OR adenoma* OR endometrioid carcinoma* OR ovarian maligna* OR borderline
