# Supplementary material for: Risk Factors for Ovarian Cancer: An Umbrella Review of the Literature
Source: Cancers (Basel). 2022 May 30;14(11):2708. doi: 10.3390/cancers14112708 (PMC9179274; doi:10.3390/cancers14112708)
Supplement: Supplementary file 1 [file cancers-14-02708-s001.zip › Appendix S2 Protocol.pdf]

SUPPLEMENTARY PROTOCOL

Introduction

Ovarian cancer is the deadliest gynaecological cancer in high-income countries with an estimated 7, 400 new cases diagnosed and 4, 100 deaths in the United Kingdom (UK) every year.[1] Ovarian cancer causes 2.5% of cancers in women but 5% of deaths, due to a combination of lack of screening with resultant delays in diagnosis.[1] Advances in treatment through a combination of surgery, chemotherapy and PARP inhibitors[2] have improved the all stages 5-year survival in the UK from 21% in 1970 to 42% in 2017.[1] Unfortunately four out of five women are diagnosed with advanced disease where the 5-year survival drops to 27%.[1]

Factors associated with developing ovarian cancer include genetic mutations e.g., BRCA mutations, older age and a family history of ovarian cancer. There are several other postulated non-genetic risk factors e.g., obesity, post-menopausal hormone use and smoking. Most of these postulated risk factors have derived from retrospective case-control studies however which are prone to recall bias. Moreover, publication bias in current literature is also common where studies with positive results being published preferentially over studies with insignificant findings, potentially leading to only 50% of studies being published.[3] This was illustrated by a recent umbrella review which concluded that only three risk factors had strong evidence for an association with endometrial cancer without a hint of bias, despite several claims of robust evidence for many other risk factors.[4]

We will perform an umbrella review of meta-analyses or systematic reviews to evaluate the robustness of the current evidence and existence for potential bias in the currently published literature which researches the association between putative risk factors and ovarian cancer.

Methods

Literature search

Two researchers will independently search PubMed, Cochrane Database of systematic reviews and Embase from inception for meta-analyses or systematic reviews investigating the association between any non-genetic risk factors and ovarian cancer.

We have developed the following as a search algorithm: “hip circumference OR waist circumference OR weight OR body mass index OR BMI OR aspirin OR NSAIDS or non-steroidal anti-inflammatory drugs OR diabetes OR endometriosis OR smoking OR oral contraceptive pills OR menarche OR parity OR hormone replacement therapy OR alcohol OR caffeine* AND (ovarian cancer OR ovarian carcinoma OR ovarian neoplasm OR borderline OR ovarian tumor*) AND (meta-analysis OR systematic review)”.

We will additionally hand search references of all included papers.

Two researchers will review the titles, abstracts, and full texts of the eligible papers. Any discrepancies will be resolved by consensus with a third researcher.

Eligibility and data extraction

Only meta-analyses or systematic reviews of epidemiological studies about human subjects will be considered to be eligible. We will exclude studies in which non-genetic risk factors are not the exposure of interest (such as BRCA gene mutations), where ovarian cancer incidence or mortality is not the outcome of interest, and meta-analyses of prognostic studies associating an exposure of interest with ovarian cancer survival or mortality among patients already diagnosed with ovarian cancer. Furthermore, articles that do not present comprehensive study specific data (relative risks, 95% confidence intervals, and numbers of cases and population) will also be excluded. Separate meta-analyses on more than one outcome or exposure of interest within one article (such different categories of exposure of interest) will be reviewed separately. Whenever more than one meta-analysis exists on the same outcome and category of exposure, the meta-analysis with the largest number of cohort studies will be selected.

We will extract from each individual study in each included meta-analysis:
- the name of first author
- the year of publication
- the outcome studied
- type of exposure (BMI, alcohol intake, parity etc.)
- the epidemiological design (case-control, cohort, cross-sectional etc.)
- from case-control studies the number of cases and controls
- from the cohort studies the number of cases and population
- the maximally adjusted relative risk (OR, HR, RR, etc.)

- the 95% confidence intervals.

Assessment of summary effects and heterogeneity

We will estimate the summary effects using both fixed and inverse variance random effects methods.[5]

We will use the *P*-value of the Cochran Q test and the I^2 metric with 95% confidence intervals to assess for between-study heterogeneity which reflects the proportion of the total variation across studies beyond chance, caused by either genuine diversity between studies or bias.[6] We will also calculate the 95% prediction intervals for the summary random effects estimates for each included meta-analysis to further account for any between-study heterogeneity.[7]

Assessment of small study effects

To assess for any small study effects, where smaller studies tend to give higher estimates than larger studies, we will implement the Egger's regression asymmetry test.[8] The small study effect can indicate reporting biases as well as true heterogeneity between small and large studies.[9]

Evaluation of excess significance

To investigate whether the observed number of studies with nominally significant results ("positive" studies, $P<0.05$) was different from the expected number of significant results we will use the excess significant test.[10]

The expected number of significant studies in each meta-analysis was calculated from the sum of the statistical power estimates for each component study with an algorithm using a non-central t distribution.[11,12] The power estimates of each component study again depend on the plausible effect size for the tested association in question. The actual size of the true effect in each meta-analysis cannot be known, but it can be assumed to be the effect of the largest study (smallest standard error) in each meta-analysis. Excess significant for individual meta-analyses was determined as $P<0.10$ (one sided $P<0.05$ with N of observed $>N$ of expected).[10]

Credibility ceilings

We will employ credibility ceilings as one method of sensitivity analysis. Every study has a probability *c* (credibility ceiling) that the true effect size of the study is in a different direction from the point estimate. The greatest certainty that each observational study reports the true effect size is (100-c) %. The sensitivity analysis that uses the 'credibility ceilings' aims to

account for these methodological limitations of the observational studies that may lead to spurious results by inflating the variance but not the point estimate of a single study.[13] The pooled effect size as well as the inter-study heterogeneity is then re-estimated using the inflated variances.[13,14] We will estimate the credibility ceiling of each meta-analysis where the last estimate before the estimate becomes statistically insignificant.

Grading the evidence

We will categorise the results of the analysis into four classifications:

a. Strong: *P*<10E-6 (random effects model); >1000 cases; *P*<0.05 of the largest study in a meta-analysis; between-study heterogeneity $I^2<50\%$; no evidence of small study effect; 95% prediction interval excludes the null value; no evidence of excess significance; and the association survives the 10% credibility ceiling
b. Highly suggestive: *P*<10E-6 (random effect model); inclusion of >1000 cases; *P*<0.05 of the largest study in a meta-analysis.
c. Suggestive associations: *P*<10E-3 (random effect model); and inclusion of >1000 cases
d. Weak associations: *P*<0.05 (random effect model).

Analyses will be done separately for cohort studies only (two or more in a meta-analysis) and all study types separately.

All calculations will be performed in STATA version 15.

References:

1. UK CR. Ovarian cancer statistics. http://www.cancerresearchuk.org/health-professional/cancer-statistics/statistics-by-cancer-type/ovarian-cancer#heading-One (accessed 2 February 2018).
2. Querleu D, Planchamp F, Chiva L, et al. European Society of Gynaecological Oncology (ESGO) Guidelines for Ovarian Cancer Surgery. *Int J Gynecol Cancer* 2017; **27**(7): 1534-42.
3. Dwan K, Gamble C, Williamson PR, Kirkham JJ, Reporting Bias G. Systematic review of the empirical evidence of study publication bias and outcome reporting bias - an updated review. *PLoS One* 2013; **8**(7): e66844.
4. Raglan O, Kalliala I, Markozannes G, et al. Risk factors for endometrial cancer: An umbrella review of the literature. *Int J Cancer* 2019; **145**(7): 1719-30.
5. DerSimonian R, Laird N. Meta-analysis in clinical trials. *Control Clin Trials* 1986; **7**(3): 177-88.

6. Ioannidis JP, Patsopoulos NA, Evangelou E. Uncertainty in heterogeneity estimates in meta-analyses. *BMJ* 2007; **335**(7626): 914-6.
7. Riley RD, Higgins JP, Deeks JJ. Interpretation of random effects meta-analyses. *BMJ* 2011; **342**: d549.
8. Egger M, Davey Smith G, Schneider M, Minder C. Bias in meta-analysis detected by a simple, graphical test. *BMJ* 1997; **315**(7109): 629-34.
9. Sterne JA, Sutton AJ, Ioannidis JP, et al. Recommendations for examining and interpreting funnel plot asymmetry in meta-analyses of randomised controlled trials. *BMJ* 2011; **343**: d4002.
10. Ioannidis JP, Trikalinos TA. An exploratory test for an excess of significant findings. *Clin Trials* 2007; **4**(3): 245-53.
11. Tsilidis KK, Papatheodorou SI, Evangelou E, Ioannidis JP. Evaluation of excess statistical significance in meta-analyses of 98 biomarker associations with cancer risk. *J Natl Cancer Inst* 2012; **104**(24): 1867-78.
12. Tsilidis KK, Panagiotou OA, Sena ES, et al. Evaluation of excess significance bias in animal studies of neurological diseases. *PLoS Biol* 2013; **11**(7): e1001609.
13. Salanti G, Ioannidis JP. Synthesis of observational studies should consider credibility ceilings. *J Clin Epidemiol* 2009; **62**(2): 115-22.
14. Papatheodorou SI, Tsilidis KK, Evangelou E, Ioannidis JP. Application of credibility ceilings probes the robustness of meta-analyses of biomarkers and cancer risk. *J Clin Epidemiol* 2015; **68**(2): 163-74.
