# Supplementary material for: Risk Factors for Ovarian Cancer: An Umbrella Review of the Literature
Source: Cancers (Basel). 2022 May 30;14(11):2708. doi: 10.3390/cancers14112708 (PMC9179274; doi:10.3390/cancers14112708)
Supplement: Supplementary file 1 [file cancers-14-02708-s001.zip › Appendix S3 Methods and Results.pdf]

SUPPLEMENTARY METHODS AND RESULTS

Data Extraction

We extracted the name of the first author and the year of publication, the modifiable exposure and outcome studied, and the summary effect (relative risk, odds risk, hazard ratio, standard incidence ratio, standard mortality ratio), and 95% confidence intervals (CI) or standard error (SE) from each eligible meta-analysis. From each individual study in a meta-analysis, we then extracted the first author and the publication year, epidemiological design (cohort, case control), number of cancer cases and controls in case- control studies or the number of cases and total population or person years in cohort studies, maximally adjusted relative risk (odds ratio in case-control studies, risk ratio or hazard ratio in cohort studies and the 95% confidence intervals. Two investigators (EW and OR) independently performed the data extraction. Disagreements were resolved and consensus reached by discussion with a third investigator (IK).

Meta-analyses were evaluated as they were originally presented; expansion of one meta-analysis with studies detected by another on the same topic was beyond the scope of this review.

Data Analysis

Assessment of summary effect, heterogeneity and prediction intervals

For each exposure, we calculated the summary effect and 95% confidence interval using both fixed and inverse variance weighted random effects methods.[1] We used the Cochran Q test[2] and the I^2 metric of inconsistency[3] including its 95% confidence intervals[4] to assess for inter-study heterogeneity. The I^2 metric ranges between 0% and 100% and could reflect either genuine diversity within the studies, chance or bias. A further assessment of inter-study heterogeneity was performed by calculating the 95% prediction intervals (PI) for the summary random effect estimates. The PI enables direct comparison with future clinically relevant effect estimates as it provides a range within which the effect estimate is predicted to fall[5,6].

Assessment of small study effects

To evaluate whether the smaller studies in a meta-analysis were creating an exaggerated risk estimate relative to the larger studies, we used Egger's test at $p<0.10$[7] with the the random-

effects summary estimate being further away from the null-value compared to the point estimate of the largest study in a meta-analysis (i.e. the study with the smallest standard error). Small study effects can occur due to publication or reporting bias, true heterogeneity or chance[8].

Evaluation of excess statistical significant bias

The test for excess significance aims to evaluate whether the observed (O) number of statistically significant results (p<0.05) in the studies included in a meta-analysis is too large compared to the expected (E) number[4]. The number of significant studies that were expected in each meta-analysis was calculated using the sum of the statistical power estimates for each included study using non-central t-distribution[4,9]. The effect of the largest study (i.e. has the smallest standard error) was used as the plausible effect size as the true effect size is not known. Sensitivity analyses were performed using the summary fixed and random effect estimates as alternative plausible effect sizes. Excess significance for each individual meta-analysis was defined as two-sided P<0.10.

Evaluation of the quality of included meta-analyses

To date there is no quality assessment tool specifically designed for umbrella reviews. We used the AMSTAR 2 tool[10] as a proxy to assess the methodological quality of the 73 included publications. The World Cancer Research Fund Continuous Update Project (WCRF CUP) report[11] was not included in this assessment, having already been subjected to extensive peer review processes.

The AMSTAR 2 tool places more weight on seven of the sixteen questions, with unsatisfactory answers to any of the seven being regarded as a critical weakness. The four grades range from ‘high’ to ‘critically low’ with ‘high’ having zero or one non-critical weakness in the study and ‘critically low’ having more than one critical flaw with or without non-critical weaknesses. The critical weaknesses detail an ‘a priori’ published protocol, a comprehensive literature search, a list of excluded studies, satisfactory risk of bias assessment for each included study, appropriate statistical analysis, adequate discussion regarding any bias detected and an appropriate investigation of publication bias. As the AMSTAR 2 tool aims to evaluate systematic reviews of randomised and non-randomised trials, some of the criteria were not applicable to umbrella reviews.

Evidence Grading

A description of the individual assessment criteria used to classify meta-analyses into evidence grades is presented in the table below:

Details of criteria for evidence grading

Assessment criteria / Evidence Grade	Summary random effect (*P*-value)	No. of cases	Heterogeneity (I^2)	95% prediction interval exc. null hypothesis	Small study effects	Excess significance	*P*-value of largest study
**Strong**	$<10^{-6}$	>1,000	<50%	Yes	None demonstrated	None demonstrated	x
**Highly suggestive**	$<10^{-6}$	>1,000	x	x	x	x	<0.05
**Suggestive**	$<10^{-3}$	>1,000	x	x	x	x	x
**Weak**	<0.05	x	x	x	x	x	x

Sensitivity analysis and credibility ceilings

We used a sensitivity tool called a credibility ceiling[12] which is based on the assumption that a single observational study has a probability *c* (credibility ceiling), or maximum certainty (100-*c*)%, that the true effect size of the study is in a different direction from the one suggested by the point estimate. In our review, we presented the credibility ceiling at which the association was not significant (Supplementary Table 7). The summary effect sizes and the inter-study heterogeneity were re-examined using credibility ceilings. By using this tool in sensitivity analysis, we aim to accounts for possible methodological limitations of the observational studies, which can cause overestimation of the combined effect estimates[12,13]. For each meta-analysis and given a ceiling of c%, the likelihood ratio of the real effect size being in the direction indicated by the summary estimate for the corresponding unit increase (or level) of exposure was computed[12].

SUPPLEMENTARY RESULTS

Characteristics of the meta-analyses

A total of 55 risk factors were examined in the 212 meta-analyses, belonging broadly to eight categories.

Details of risk factors assessed

**Risk Factor Categories**		
**Anthropometric *n*=7**	Hip circumference<br>Waist circumference<br>Waist to hip ratio<br>Weight gain<br>Weight<br>BMI<br>Height	
**Dietary *n*=17**	Dairy<br>Type of dairy intake<br>Fruit<br>Meat<br>Eggs<br>Polyphenols<br>Vegetables<br>Alcohol<br>Tea	Fish<br>Fat<br>Acrylamide<br>Dietary inflammatory index<br>Fibre<br>Protein<br>Coffee<br>Dietary vitamins and minerals
**Physical activity and sedentary behaviour *n*=2**	Recreational activity<br>Sedentary behaviour	
**Past medical history *n*=3**	SLE<br>DM<br>PCOS	
**Past drug history *n*=9**	Aspirin<br>NSAIDS<br>Acetaminophen<br>HRT<br>OCP	Statins<br>Metformin<br>Bisphosphonates<br>Antidepressants
**Biomarkers n=4**	CRP<br>IL6	

	TNFα<br>TNFR2	
**Past gynaecological history *n*=11**	Number of terminations<br>Age at menarche<br>Tubal ligation<br>Hysterectomy<br>PID<br>Breastfeeding	Multiple births<br>Parity<br>IVF<br>Age at last birth<br>Endometriosis
**Carcinogens *n*=3**	Talcum powder<br>Smoking<br>Asbestos	

Abbreviations: BMI, body mass index; CRP c- reactive protein; DM, diabetes mellitus; HRT, hormone replacement therapy; IL6, interleukin 6; IVF, in vitro fertilisation; NSAIDS, non steroidal anti-inflammatory drugs; OCP, oral contraceptive pill; PCOS, polycystic ovarian syndrome; SLE, systemic lupus erythematosus; TNFα, tumour necrosis factor α; TNFR2, tumour necrosis factor receptor 2

Quality assessment

76% (55/72) of included meta-analyses stated 'a priori' published protocols and 89% (64/72) described a literature review. Only 4% (3/74) both listed excluded studies and justified their exclusion, whilst a further 35% (25/72) declared the excluded studies without explanation. Most meta-analyses provided satisfactory methods when assessing for risk of bias (78%, 56/72), when combining the findings (97%, 70/72), when accounting for risk of bias from individual studies in interpretation of the main results (81%, 58/72) and when investigating and discussing any small study bias (81%, 58/72). Of the remaining components of the AMSTAR 2, the majority of included meta-analyses fulfilled the criteria except for completing the study selection in duplicate (44%, 32/72) and assessing the potential impact of risk of bias of individual studies on the main results (33%, 24/72).

References:

1. DerSimonian R LN. Meta-analysis in clinical trials. *Controlled clinical trials* 1986; **7**(3): 177-88.
2. WG C. The combination of estimates from different experiments. *Biometrics* 1954; **10**: 101-29.
3. Higgins JP, Thompson SG, Deeks JJ, Altman DG. Measuring inconsistency in meta-analyses. *BMJ* 2003; **327**(7414): 557-60.
4. Ioannidis JP, Trikalinos TA. An exploratory test for an excess of significant findings. *Clin Trials* 2007; **4**(3): 245-53.
5. IntHout J, Ioannidis JP, Rovers MM, Goeman JJ. Plea for routinely presenting prediction intervals in meta-analysis. *BMJ Open* 2016; **6**(7): e010247.

6. Riley RD, Higgins JP, Deeks JJ. Interpretation of random effects meta-analyses. *BMJ* 2011; **342**: d549.
7. Egger M, Davey Smith G, Schneider M, Minder C. Bias in meta-analysis detected by a simple, graphical test. *BMJ* 1997; **315**(7109): 629-34.
8. Nuesch E, Trelle S, Reichenbach S, et al. Small study effects in meta-analyses of osteoarthritis trials: meta-epidemiological study. *BMJ* 2010; **341**: c3515.
9. Tsilidis KK, Papatheodorou SI, Evangelou E, Ioannidis JP. Evaluation of excess statistical significance in meta-analyses of 98 biomarker associations with cancer risk. *J Natl Cancer Inst* 2012; **104**(24): 1867-78.
10. Shea BJ, Reeves BC, Wells G, et al. AMSTAR 2: a critical appraisal tool for systematic reviews that include randomised or non-randomised studies of healthcare interventions, or both. *BMJ* 2017; **358**: j4008.
11. WCRF/AICR. The Associations between Food, Nutrition, and Physical Activity and the Risk of Ovarian Cancer Continuous Update Project Report. *WCRF/ AICR* 2013.
12. Salanti G, Ioannidis JP. Synthesis of observational studies should consider credibility ceilings. *J Clin Epidemiol* 2009; **62**(2): 115-22.
13. Papatheodorou SI, Tsilidis KK, Evangelou E, Ioannidis JP. Application of credibility ceilings probes the robustness of meta-analyses of biomarkers and cancer risk. *J Clin Epidemiol* 2015; **68**(2): 163-74.
