# Supplementary material for: Risk Factors for Ovarian Cancer: An Umbrella Review of the Literature
Source: Cancers (Basel). 2022 May 30;14(11):2708. doi: 10.3390/cancers14112708 (PMC9179274; doi:10.3390/cancers14112708)
Supplement: Supplementary file 1 [file cancers-14-02708-s001.zip › Table S1 .pdf]

Supplementary Table S1: Excluded duplicate studies and studies selected in their place to be included in the analysis.

Exposure	Exposure contrast	Author, year	Outcome	N[a] studies	N cohort studies	Summary relative risk & 95% CI[b]	Included	Evidence grade[c]
Alcohol	Any vs none	Rota 2012[1]	OC	27	4	1.00 (0.95-1.05)	No	
Alcohol	Any vs none	Yan-Hong 2015[2]	OC	13	13	1.03 (0.96-1.10)	Yes	NS
Alcohol	Light vs none	Rota 2012[1]	OC	20	3	0.97 (0.92-1.02)	No	
Alcohol	Light vs none	Yan-Hong 2015[2]	OC	13	13	0.96 (0.93-1.00)	Yes	NS
Alcohol	Moderate vs none	Rota 2012[1]	OC	16	3	1.03 (0.96-1.11)	No	
Alcohol	Moderate vs none	Yan-Hong 2015[2]	OC	13	13	1.08 (0.92-1.27)	Yes	NS
Alcohol	High vs none	Rota 2012[1]	OC	4	0	1.09 (0.80-1.50)	No	
Alcohol	High vs none	Yan-Hong 2015[2]	OC	13	13	0.99 (0.88-1.12)	Yes	NS
Aspirin	Any vs none	Bonovas 2005[3]	OC	9	3	0.90 (0.80-1.06)	No	
Aspirin	Ever vs never	Qiao 2018[4]	OC	21	8	0.89 (0.83-0.95)	No	
Aspirin	Ever vs never	Ni 2013[5]	OC	17	3	0.91 (0.82-1.02)	No	
Aspirin	Ever vs never	Zhang 2016[6]	OC	22	8	0.89 (0.83-0.96)	Yes	Weak
Aspirin	Regular vs non regular	Bosetti 2012[7]	OC	11	5	0.91 (0.81-1.01)	No	
Aspirin	Regular vs non regular	Trabert 2014[8]	OC	12	0	0.91 (0.84-0.99)	No	
Aspirin	Regular vs non regular	Murphy 2012[9]	OC	14	4	0.96 (0.85-1.08)	Yes	NS
NSAIDS non aspirin	Regular vs non regular	Bonovas 2005[3]	OC	6	6	0.86 (0.68-1.08)	No	
NSAIDS non aspirin	Ever vs never	Ni 2013[5]	OC	7	3	0.89 (0.74-1.08)	No	
NSAIDS non aspirin	Ever vs never	Baandrup 2013[10]	OC	16	6	0.94 (0.84-1.06)	Yes	Weak
Coffee	Highest vs lowest	Braem 2012[11]	OC	7	7	1.13 (0.89-1.46)	No	
Coffee	Highest vs lowest	Berretta 2018[12]	OC	7	7	1.06 (0.89-1.67)	No	
Coffee	Highest vs lowest	Salari-Moghaddam 2019[13]	OC	13	13	1.10 (0.90-1.35)	Yes	NS
Breastfeeding	Ever vs never	Chowdhury 2015[14]	OC	40	5	0.70 (0.64-0.77)	No	
Breastfeeding	Ever vs never	Feng 2014[15]	OC	19	3	0.66 (0.57-0.76)	No	
Breastfeeding	Ever vs never	Li 2014[16]	OC	40	5	0.69 (0.34-0.76)	Yes	Highly suggestive
Breastfeeding	Ever vs never	Luan 2013[17]	OC	32	5	0.76 (0.69-0.83)	No	
Breastfeeding	Ever vs never, PA	Park 2016[18]	OC	5	0	1.17 (1.02-1.33)	No	
Breastfeeding	Ever vs never	Ip 2007[19]	OC	9	0	0.79 (0.61-0.91)	No	
Breastfeeding	Ever vs never	WCRF CUP 2013[20]	OC	3	3	0.90 (0.75-1.08)	No	
Tubal ligation	Yes vs no, PA	Park 2016[18]	OC	2	0	1.44 (1.33-1.56)	No	
Tubal ligation	Ever vs never	Sieh 2013[21]	OC	13	0	0.71 (0.66-0.77)	No	
Tubal ligation	Ever vs never	Rice 2012[22]	OC	36	6	0.70 (0.64-0.75)	No	
Tubal ligation	Ever vs never	Cibula 2011[23]	OC	21	5	0.69 (0.64-0.75)	No	
Tubal ligation	Ever vs never	Yoon 2016[24]	OC	3	1	0.51 (0.35-0.75)	No	
Tubal ligation	Ever vs never	Wang 2016[25]	OC	25	0	0.70 (0.60-0.81)	Yes	Suggestive
BMI	25-29.9kg/m^2 vs normal	Poorolajal 2014[26]	OC	13	13	1.08 (0.97-1.19)	Yes	NS
BMI	25-29.9kg/m^2 vs normal	Liu 2015[27]	OC	25	12	1.08 (1.02-1.14)	No	
BMI	25-29.9kg/m^2 vs normal, OR	Olsen 2007[28]	OC	14	6	1.16 (1.01-1.32)	No	
BMI	≥30kg/m^2 vs normal	Poorolajal 2014[26]	OC	13	13	1.27 (1.16-1.38)	Yes	Strong
BMI	≥30kg/m^2 vs normal	Liu 2015[27]	OC	25	12	1.23 (1.10-1.39)	No	
BMI	≥30kg/m^2 vs normal, OR	Olsen 2007[28]	OC	16	8	1.30 (1.12-1.50)	No	
BMI	Per 5kg/m2 increase	WCRF Cup 2013[20]	OC	34 (inc pooled study)	22	1.06 (1.02-1.11)	No	
BMI	Per 5kg/m2 increase	Aune 2015[29]	OC	25	25	1.07 (1.04-1.11)	Yes	Suggestive

Cruciferous vegetables	High vs low	Hu 2015[30]	OC	8	4	0.89 (0.81-0.99)	No	
Cruciferous vegetables	High vs low	Han 2014[31]	OC	11	4	0.89 (0.80-1.00)	Yes	Weak
Metformin	Any vs none	Dilokthornsakul 2013[32]	OC	3	0	0.67 (0.44-1.04)	No	
Metformin	Ever vs never	Li 2016[33]	OC	4	1	0.71 (0.54-0.92)	No	
Metformin	Ever vs never	Wen 2019[34]	OC	3	2	0.18 (0.12-0.25)	Yes	Highly suggestive
Diabetes Mellitus	Yes vs no	Wang 2017[35]	OC	14	14	1.19 (1.06-1.34)	No	
Diabetes Mellitus	Yes vs no	Zhang 2017[36]	OCM	17	17	1.32 (1.14-1.52)	Yes	Suggestive
Talc	Exposed vs unexposed	Gross 1995[37]	OC	9	0	1.27 (1.09-1.48)	No	
Talc: on contraceptive diaphragm	Any vs none	Huncharek 2007[38]	OC	9	0	1.00 (0.77-1.29)	Yes	NS
Talc	Ever vs never	Terry 2013[39]	OC	8	0	1.24 (1.15-1.33)	No	
Talc	Ever vs never	Berge 2017[40]	OC	27	3	1.26 (1.17-1.35)	Yes	Highly suggestive
Talc	Ever vs never	Huncharek 2003[41]	OC	16	1	1.33 (1.16-1.45)	No	
Endometriosis	Any vs none; CC/2 arm cohort	Kim 2014[42]	OC	21	3	1.61 (1.01-1.99)	No	
Endometriosis	Any vs none; single arm cohort	Kim 2014[42]	OC	5	5	1.80 (1.28-2.53)	No	
Endometriosis	Ever vs never	Wang 2016[25]	OC	12	0	1.42 (1.28-1.97)	No	
Endometriosis	Any vs none	Li 2019[43]	OC	25	16	1.42 (1.28-1.47)	Yes	Strong
Animal fat	Highest vs lowest	Huncharek 2001[44]	OC	3	1	1.70 (1.43-2.03)	No	
Animal fat	Highest vs lowest	Hou 2015[45]	OC	8	3	1.15 (0.95-1.39)	No	
Animal fat	Highest vs lowest	Qiu 2016[46]	OC	10	5	1.21 (0.99-1.47)	Yes	NS
Dairy fat	Highest vs lowest	Hou 2015[45]	OC	6	5	1.05 (0.92-1.19)	No	
Dairy fat	Highest vs lowest	Qiu 2016[46]	OC	3	2	1.05 (0.92-1.19)	Yes	NS
Monounsaturated fat	Highest vs lowest	Hou 2015[45]	OC	10	3	0.98 (0.84-1.13)	No	
Monounsaturated fat	Highest vs lowest	Qiu 2016[46]	OC	13	5	1.00 (0.90-1.10)	Yes	NS
Plant fat	Highest vs lowest	Hou 2015[45]	OC	9	3	0.93 (0.77-1.13)	No	
Plant fat	Highest vs lowest	Qiu 2016[46]	OC	6	5	0.95 (0.83-1.09)	Yes	
Saturated fat	Highest vs lowest	Qiu 2016[46]	OC	11	6	1.10 (0.99-1.21)	Yes	NS
Saturated fat	Highest vs lowest	Huncharek 2001[44]	OC	3	1	1.20 (1.04-1.29)	No	
Total fat	Highest vs lowest	Huncharek 2001[44]	OC	7	1	1.26 (1.11-1.42)	No	
Total fat	Highest vs lowest	Hou 2015[45]	OC	14	3	1.12 (0.95-1.33)	No	
Total fat	Highest vs lowest	Qiu 2016[46]	OC	17	6	1.19 (1.04-1.37)	Yes	Weak
Fibre	Highest vs lowest	Huang 2018[47]	OC	17	4	0.76 (0.63-0.92)	No	
Fibre	Highest vs lowest	Xu 2018[48]	OC	19	5	0.70 (0.57-0.87)	Yes	NS
Hysterectomy	Yes vs no	Jordan 2013[49]	OC	21	5	0.81 (0.72-0.92)	No	
Hysterectomy	Yes vs no	Rice 2012[22]	OC	36	5	0.74 (0.65-0.84)	No	
Hysterectomy	Yes vs no	Huo 2019[50]	OC	18	0	0.97 (0.83-1.12)	No	
Hysterectomy	Yes vs no	Wang 2016[25]	OC	22	0	0.97 (0.81-1.15)	Yes	NS
Height	Per 10cm	Aune 2015[29]	OC	16	16	1.16 (1.11-1.20)	Yes	Strong
Height	Per 5cm	WCRF CUP 2013[20]	OC	13	13	1.07 (1.05-1.10)	No	
CRP	High vs low	Zeng 2016[51]	OC	7	1	1.91 (1.51-2.40)	No	
CRP	Middle vs low	Zeng 2016[51]	OC	7	1	1.13 (0.96-1.33)	No	
CRP	High vs low	Li 2017[52]	OC	7	1	1.35 (1.06-1.71)	Yes	Weak
CRP	Middle vs low	Li 2017[52]	OC	7	1	1.52 (1.01-2.27)	Yes	Weak
HRT	Ever vs never	Negri 1999[53]	OC	4	0	1.71 (1.30-2.25)	No	
HRT	Ever vs never	Collab Group 2015[54]	OC	17	16	1.20 (1.13-1.28)	Yes	Strong
Dietary α-carotene	Per 600ug/day	WCRF CUP 2013[20]	OC	3	3	1.00 (0.98-1.01)	No	
Dietary α-carotene	Per 600ug/day	Koushik 2006[55]	OC	11	11	1.00 (0.95-1.05)	Yes	NS

Dietary β- Carotene	Per 2500ug/day	WCRF CUP 2013[20]	OC	4	4	0.99 (0.92-1.07)	No	
Dietary β- Carotene	Per 2500ug/day	Koushik 2006[55]	OC	11	11	0.98 (0.93-1.03)	Yes	NS
Dietary β-cryptoxanthin	Per 100ug/day	WCRF CUP 2013[20]	OC	3	3	1.02 (0.90-1.15)	No	
Dietary β-cryptoxanthin	Per 100ug/day	Koushik 2006[55]	OC	11	11	0.99 (0.97-1.02)	Yes	NS
Dietary lycopene	Per 4000ug/day	WCRF CUP 2013[20]	OC	3	3	1.00 (0.93-1.17)	No	
Dietary lycopene	Per 4000ug/day	Koushik 2006[55]	OC	11	11	1.02 (0.98-1.06)	Yes	NS
Tea	Highest vs lowest, HR	Braem 2012[11]	OC	6	6	0.88 (0.71-1.09)	No	
Tea	Any vs none	Zhou 2007[56]	OC	9	2	0.84 (0.66-1.07)	No	
Tea	Highest vs lowest	Zhan 2017[57]	OC	18	7	0.89 (0.80-1.00)	Yes	Weak
Green tea	Highest vs lowest	Butler 2011[58]	OC	4	0	0.63 (0.42-0.93)	Yes	Weak
Green tea	Highest vs lowest	Nagle 2010[59]	OC	2	0	0.58 (0.33-1.01)	No	
Parity	Parous vs Nullip, PA	Park 2016[18]	OC	9	7	0.80 (0.61-1.05)	Yes	NS
Parity	Nullip vs Parous	Negri 1991[60]	OC	3	0	1.40 (1.10-1.70)	No	
Parity	Parous vs Nullip	Whittemore 1993[61]	OC	6	0	0.47 (0.40-0.56)	No	
Acrylamide	Highest vs lowest	Pelucchi 2011[62]	OC	3	2	1.09 (0.76-1.57)	No	
Acrylamide	Highest vs lowest	Pelucchi 2015[63]	OC	4	2	1.12 (0.85-1.47)	Yes	NS
Acrylamide	Per 10ug/day increase	Pelucchi 2011[62]	OC	3	2	1.01 (0.94-1.08)	No	
Acrylamide	Per 10ug/day increase	Pelucchi 2015[63]	OC	4	2	1.02 (0.96-1.09)	Yes	NS
Sedentary behaviour	Highest vs lowest	Shen 2014[64]	OC	2	2	1.26 (0.87-1.82)	No	
Sedentary behaviour	Highest vs lowest	Schmid 2014[65]	OC	5	2	1.22 (0.97-1.70)	Yes	NS
OCP	Ever vs never	Coll Gr 2008[66]	OC	45	13	0.74 (0.70-0.78)	Yes	Strong
OCP	Ever vs never	Hankinson 1992[67]	OC	20	3	0.72 (0.54-0.97)	No	
OCP	Never vs Ever, PA	Park 2016[18]	OC	2	1	1.87 (0.89-3.94)	No	
OCP	Ever vs never	Zhong 2015[68]	OC	3	3	0.58 (0.35-0.94)	No	

Abbreviations: BMI, body mass index; CC, case control; CI, confidence interval; Coll, collaborative; CRP, C reactive protein; Gr, group; HR, hazard ratio; HRT, hormone replacement therapy; inc, including; kg, kilogram; m^2, metre squared; NS, non significant; NSAID, non-steroidal anti-inflammatory drug; OCP, oral contraceptive pill; OR, odds ratio; PA, pooled analysis; ug, microgram; yoa, years of age; WCRF, World Cancer Research Fund

Key:

[a] Number of studies

[b] Summary relative risk of random effects model including all study types

[c] Summary of evidence grading criteria:

Weak	P<0.05[d]
Suggestive	$P<10^{-3}$[d]; >1,000 cases
Highly suggestive	$P<10^{-6}$[d]; >1,000 cases; P<0.05 of the largest study in a meta-analysis
Strong	$P<10^{-6}$[d]; >1,000 cases; P<0.05 of the largest study in a meta-analysis; $I^2<50\%$; no small study effect[e]; prediction interval excludes the null value; no excess significance bias[f]

[d] P value of summary random effects estimate

[e] Small study effect is based on the P-value from the Egger's regression asymmetry test (P>0.1) where the random effects summary estimate was larger compared to the point estimate of the largest study in a meta-analysis
[f] Based on the p-value (P>0.1) of the excess significance test using the largest study (smallest standard error) in a meta-analysis as the plausible effect size.

References:

1. Rota M, Pasquali E, Scotti L, et al. Alcohol drinking and epithelial ovarian cancer risk. a systematic review and meta-analysis. *Gynecol Oncol* 2012; **125**(3): 758-63.
2. Yan-Hong H, Jing L, Hong L, Shan-Shan H, Yan L, Ju L. Association between alcohol consumption and the risk of ovarian cancer: a meta-analysis of prospective observational studies. *BMC Public Health* 2015; **15**: 223.
3. Bonovas S, Filioussi K, Sitaras NM. Do nonsteroidal anti-inflammatory drugs affect the risk of developing ovarian cancer? A meta-analysis. *Br J Clin Pharmacol* 2005; **60**(2): 194-203.
4. Qiao Y, Yang T, Gan Y, et al. Associations between aspirin use and the risk of cancers: a meta-analysis of observational studies. *BMC Cancer* 2018; **18**(1): 288.
5. Ni X, Ma J, Zhao Y, Wang Y, Wang S. Meta-analysis on the association between non-steroidal anti-inflammatory drug use and ovarian cancer. *Br J Clin Pharmacol* 2013; **75**(1): 26-35.
6. Zhang D, Bai B, Xi Y, Wang T, Zhao Y. Is aspirin use associated with a decreased risk of ovarian cancer? A systematic review and meta-analysis of observational studies with dose-response analysis. *Gynecol Oncol* 2016; **142**(2): 368-77.
7. Bosetti C, Rosato V, Gallus S, Cuzick J, La Vecchia C. Aspirin and cancer risk: a quantitative review to 2011. *Annals of Oncology* 2012; **23**(6): 1403-15.
8. Trabert B, Ness RB, Lo-Ciganic WH, et al. Aspirin, nonaspirin nonsteroidal anti-inflammatory drug, and acetaminophen use and risk of invasive epithelial ovarian cancer: a pooled analysis in the Ovarian Cancer Association Consortium. *J Natl Cancer Inst* 2014; **106**(2): djt431.
9. Murphy MA, Trabert B, Yang HP, et al. Non-steroidal anti-inflammatory drug use and ovarian cancer risk: findings from the NIH-AARP Diet and Health Study and systematic review. *Cancer Causes Control* 2012; **23**(11): 1839-52.
10. Baandrup L, Faber MT, Christensen J, et al. Nonsteroidal anti-inflammatory drugs and risk of ovarian cancer: systematic review and meta-analysis of observational studies. *Acta Obstet Gynecol Scand* 2013; **92**(3): 245-55.
11. Braem MG, Onland-Moret NC, Schouten LJ, et al. Coffee and tea consumption and the risk of ovarian cancer: a prospective cohort study and updated meta-analysis. *Am J Clin Nutr* 2012; **95**(5): 1172-81.
12. Berretta M, Micek A, Lafranconi A, et al. Coffee consumption is not associated with ovarian cancer risk: a dose-response meta-analysis of prospective cohort studies. *Oncotarget* 2018; **9**(29): 20807-15.
13. Salari-Moghaddam A, Milajerdi A, Surkan PJ, Larijani B, Esmaillzadeh A. Caffeine, Type of Coffee, and Risk of Ovarian Cancer: A Dose-Response Meta-Analysis of Prospective Studies. *J Clin Endocrinol Metab* 2019; **104**(11): 5349-59.
14. Chowdhury R, Sinha B, Sankar MJ, et al. Breastfeeding and maternal health outcomes: a systematic review and meta-analysis. *Acta Paediatr* 2015; **104**(467): 96-113.
15. Feng LP, Chen HL, Shen MY. Breastfeeding and the risk of ovarian cancer: a meta-analysis. *J Midwifery Womens Health* 2014; **59**(4): 428-37.
16. Li DP, Du C, Zhang ZM, et al. Breastfeeding and ovarian cancer risk: a systematic review and meta-analysis of 40 epidemiological studies. *Asian Pac J Cancer Prev* 2014; **15**(12): 4829-37.
17. Luan NN, Wu QJ, Gong TT, Vogtmann E, Wang YL, Lin B. Breastfeeding and ovarian cancer risk: a meta-analysis of epidemiologic studies. *Am J Clin Nutr* 2013; **98**(4): 1020-31.
18. Park B, Park S, Shin HR, et al. Population attributable risks of modifiable reproductive factors for breast and ovarian cancers in Korea. *BMC Cancer* 2016; **16**: 5.

19. Ip S, Chung M, Raman G, et al. Breastfeeding and maternal and infant health outcomes in developed countries. *Evidence Report/Technology Assessment* 2007; (153): 1-186.
20. WCRF/AICR. The Associations between Food, Nutrition, and Physical Activity and the Risk of Ovarian Cancer Continuous Update Project Report. *WCRF/ AICR* 2013.
21. Sieh W, Salvador S, McGuire V, et al. Tubal ligation and risk of ovarian cancer subtypes: a pooled analysis of case-control studies. *Int J Epidemiol* 2013; **42**(2): 579-89.
22. Rice MS, Murphy MA, Tworoger SS. Tubal ligation, hysterectomy and ovarian cancer: A meta-analysis. *J Ovarian Res* 2012; **5**(1): 13.
23. Cibula D, Widschwendter M, Majek O, Dusek L. Tubal ligation and the risk of ovarian cancer: review and meta-analysis. *Hum Reprod Update* 2011; **17**(1): 55-67.
24. Yoon SH, Kim SN, Shim SH, Kang SB, Lee SJ. Bilateral salpingectomy can reduce the risk of ovarian cancer in the general population: A meta-analysis. *European Journal of Cancer* 2016; **55**: 38-46.
25. Wang C, Liang Z, Liu X, Zhang Q, Li S. The Association between Endometriosis, Tubal Ligation, Hysterectomy and Epithelial Ovarian Cancer: Meta-Analyses. *Int J Environ Res Public Health* 2016; **13**(11).
26. Poorolajal J, Jenabi E, Masoumi SZ. Body mass index effects on risk of ovarian cancer: a meta- analysis. *Asian Pac J Cancer Prev* 2014; **15**(18): 7665-71.
27. Liu Z, Zhang TT, Zhao JJ, et al. The association between overweight, obesity and ovarian cancer: a meta-analysis. *Jpn J Clin Oncol* 2015; **45**(12): 1107-15.
28. Olsen CM, Green AC, Whiteman DC, Sadeghi S, Kolahdooz F, Webb PM. Obesity and the risk of epithelial ovarian cancer: a systematic review and meta-analysis. *Eur J Cancer* 2007; **43**(4): 690-709.
29. Aune D, Navarro Rosenblatt DA, Chan DS, et al. Anthropometric factors and ovarian cancer risk: a systematic review and nonlinear dose-response meta-analysis of prospective studies. *Int J Cancer* 2015; **136**(8): 1888-98.
30. Hu J, Hu Y, Hu Y, Zheng S. Intake of cruciferous vegetables is associated with reduced risk of ovarian cancer: a meta-analysis. *Asia Pac J Clin Nutr* 2015; **24**(1): 101-9.
31. Han B, Li X, Yu T. Cruciferous vegetables consumption and the risk of ovarian cancer: a meta-analysis of observational studies. *Diagn Pathol* 2014; **9**: 7.
32. Dilokthornsakul P, Chaiyakunapruk N, Termrungruanglert W, Pratoomsoot C, Saokaew S, Sruamsiri R. The effects of metformin on ovarian cancer: a systematic review. *Int J Gynecol Cancer* 2013; **23**(9): 1544-51.
33. Li L, Qi X, Xu M, et al. The effects of metformin on ovarian cancer: An updated systematic review and meta-analysis. *International Journal of Clinical and Experimental Medicine* 2016; **9**(9): 17559-68.
34. Wen Q, Zhao Z, Wen J, et al. The association between metformin therapy and risk of gynecological cancer in patients: Two meta-analyses. *Eur J Obstet Gynecol Reprod Biol* 2019; **237**: 33-41.
35. Wang L, Wang L, Zhang J, Wang B, Liu H. Association between diabetes mellitus and subsequent ovarian cancer in women: A systematic review and meta-analysis of cohort studies. *Medicine (Baltimore)* 2017; **96**(16): e6396.
36. Zhang D, Zhao Y, Wang T, Xi Y, Li N, Huang H. Diabetes mellitus and long-term mortality of ovarian cancer patients. A systematic review and meta-analysis of 12 cohort studies. *Diabetes Metab Res Rev* 2017; **33**(4).
37. Gross AJ, Berg PH. A meta-analytical approach examining the potential relationship between talc exposure and ovarian cancer. *J Expo Anal Environ Epidemiol* 1995; **5**(2): 181-95.
38. Huncharek M, Muscat J, Onitilo A, Kupelnick B. Use of cosmetic talc on contraceptive diaphragms and risk of ovarian cancer: a meta-analysis of nine observational studies. *Eur J Cancer Prev* 2007; **16**(5): 422-9.
39. Terry KL, Karageorgi S, Shvetsov YB, et al. Genital powder use and risk of ovarian cancer: a pooled analysis of 8,525 cases and 9,859 controls. *Cancer Prev Res (Phila)* 2013; **6**(8): 811-21.
40. Berge W, Mundt K, Luu H, Boffetta P. Genital use of talc and risk of ovarian cancer: a meta-analysis. *Eur J Cancer Prev* 2017.

41. Huncharek M, Geschwind JF, Kupelnick B. Perineal application of cosmetic talc and risk of invasive epithelial ovarian cancer: a meta-analysis of 11,933 subjects from sixteen observational studies. *Anticancer Res* 2003; **23**(2C): 1955-60.
42. Kim HS, Kim TH, Chung HH, Song YS. Risk and prognosis of ovarian cancer in women with endometriosis: a meta-analysis. *Br J Cancer* 2014; **110**(7): 1878-90.
43. Li J, Liu R, Tang S, et al. Impact of endometriosis on risk of ovarian, endometrial and cervical cancers: a meta-analysis. *Arch Gynecol Obstet* 2019; **299**(1): 35-46.
44. Huncharek M, Kupelnick B. Dietary fat intake and risk of epithelial ovarian cancer: a meta-analysis of 6,689 subjects from 8 observational studies. *Nutr Cancer* 2001; **40**(2): 87-91.
45. Hou R, Wu QJ, Gong TT, Jiang L. Dietary fat and fatty acid intake and epithelial ovarian cancer risk: evidence from epidemiological studies. *Oncotarget* 2015; **6**(40): 43099-119.
46. Qiu W, Lu H, Qi Y, Wang X. Dietary fat intake and ovarian cancer risk: a meta-analysis of epidemiological studies. *Oncotarget* 2016; **7**(24): 37390-406.
47. Huang X, Wang X, Shang J, et al. Association between dietary fiber intake and risk of ovarian cancer: a meta-analysis of observational studies. *J Int Med Res* 2018; **46**(10): 3995-4005.
48. Xu H, Ding Y, Xin X, Wang W, Zhang D. Dietary fiber intake is associated with a reduced risk of ovarian cancer: a dose-response meta-analysis. *Nutr Res* 2018; **57**: 1-11.
49. Jordan SJ, Nagle CM, Coory MD, et al. Has the association between hysterectomy and ovarian cancer changed over time? A systematic review and meta-analysis. *Eur J Cancer* 2013; **49**(17): 3638-47.
50. Huo X, Yao L, Han X, et al. Hysterectomy and risk of ovarian cancer: a systematic review and meta- analysis. *Arch Gynecol Obstet* 2019; **299**(3): 599-607.
51. Zeng F, Wei H, Yeoh E, et al. Inflammatory Markers of CRP, IL6, TNFalpha, and Soluble TNFR2 and the Risk of Ovarian Cancer: A Meta-analysis of Prospective Studies. *Cancer Epidemiol Biomarkers Prev* 2016; **25**(8): 1231-9.
52. Li J, Jiao X, Yuan Z, Qiu H, Guo R. C-reactive protein and risk of ovarian cancer: A systematic review and meta-analysis. *Medicine (Baltimore)* 2017; **96**(34): e7822.
53. Negri E, Tzonou A, Beral V, et al. Hormonal therapy for menopause and ovarian cancer in a collaborative re-analysis of European studies. *Int J Cancer* 1999; **80**(6): 848-51.
54. Collaborative Group On Epidemiological Studies Of Ovarian C, Beral V, Gaitskell K, et al. Menopausal hormone use and ovarian cancer risk: individual participant meta-analysis of 52 epidemiological studies. *Lancet* 2015; **385**(9980): 1835-42.
55. Koushik A, Hunter DJ, Spiegelman D, et al. Intake of the major carotenoids and the risk of epithelial ovarian cancer in a pooled analysis of 10 cohort studies. *Int J Cancer* 2006; **119**(9): 2148-54.
56. Zhou B, Yang L, Wang L, et al. The association of tea consumption with ovarian cancer risk: A metaanalysis. *Am J Obstet Gynecol* 2007; **197**(6): 594 e1-6.
57. Zhan X, Wang J, Pan S, Lu C. Tea consumption and the risk of ovarian cancer: A meta-analysis of epidemiological studies. *Oncotarget* 2017.
58. Butler LM, Wu AH. Green and black tea in relation to gynecologic cancers. *Mol Nutr Food Res* 2011; **55**(6): 931-40.
59. Nagle CM, Olsen CM, Bain CJ, Whiteman DC, Green AC, Webb PM. Tea consumption and risk of ovarian cancer. *Cancer Causes Control* 2010; **21**(9): 1485-91.
60. Negri E, Franceschi S, Tzonou A, et al. Pooled analysis of 3 European case-control studies: I. Reproductive factors and risk of epithelial ovarian cancer. *Int J Cancer* 1991; **49**(1): 50-6.
61. Whittemore AS. Personal characteristics relating to risk of invasive epithelial ovarian cancer in older women in the United States. *Cancer* 1993; **71**(2 Suppl): 558-65.
62. Pelucchi C, La Vecchia C, Bosetti C, Boyle P, Boffetta P. Exposure to acrylamide and human cancer--a review and meta-analysis of epidemiologic studies. *Ann Oncol* 2011; **22**(7): 1487-99.
63. Pelucchi C, Bosetti C, Galeone C, La Vecchia C. Dietary acrylamide and cancer risk: an updated meta-analysis. *Int J Cancer* 2015; **136**(12): 2912-22.
64. Shen D, Mao W, Liu T, et al. Sedentary behavior and incident cancer: a meta-analysis of prospective studies. *PLoS One* 2014; **9**(8): e105709.
65. Schmid D, Leitzmann MF. Television viewing and time spent sedentary in relation to cancer risk: a meta-analysis. *J Natl Cancer Inst* 2014; **106**(7).

66. Collaborative Group on Epidemiological Studies of Ovarian C, Beral V, Doll R, Hermon C, Peto R, Reeves G. Ovarian cancer and oral contraceptives: collaborative reanalysis of data from 45 epidemiological studies including 23,257 women with ovarian cancer and 87,303 controls. *Lancet* 2008; **371**(9609): 303-14.
67. Hankinson SE, Colditz GA, Hunter DJ, Spencer TL, Rosner B, Stampfer MJ. A quantitative assessment of oral contraceptive use and risk of ovarian cancer. *Obstet Gynecol* 1992; **80**(4): 708-14.
68. Zhong GC, Cheng JH, Xu XL, Wang K. Meta-analysis of oral contraceptive use and risks of all-cause and cause-specific death. *Int J Gynaecol Obstet* 2015; **131**(3): 228-33.
