# Supplementary material for: Risk Factors for Ovarian Cancer: An Umbrella Review of the Literature
Source: Cancers (Basel). 2022 May 30;14(11):2708. doi: 10.3390/cancers14112708 (PMC9179274; doi:10.3390/cancers14112708)
Supplement: Supplementary file 1 [file cancers-14-02708-s001.zip › Tables S2-S9.pdf]

Supplementary Table S2: Results with random p value >0.05, not statistically significant, cohorts only

Author_year	Exposure	Exposure_contrast	RR (95% CI)	p value
Pelucchi 2015	Acylamide	Highest v Lowest	1.02 (0.71-1.48)	0.88
Pelucchi 2015	Acylamide	10ug/day increase	1.02 (0.91-1.16)	0.71
Yan -Hong 2015	Alcohol	None vs some	1.03 (0.96-1.10)	0.47
Yan -Hong 2015	Alcohol	Low vs none	0.96 (0.93-1.00)	0.06
Yan -Hong 2015	Alcohol	Moderate vs none	1.08 (0.92-1.27)	0.33
Yan -Hong 2015	Alcohol	High vs none	0.99 (0.88-1.12)	0.90
Kim 2010	Wine	Ever vs never	1.44 (0.74-2.82)	0.28
WCRF Cup 2013	Wine	per 10g increase per day	1.16 (0.76-1.75)	0.50
Zhang 2016	Aspirin	Ever vs never	0.95 (0.85-1.05)	0.29
Zhang 2016	Aspirin	Ever vs never; cohort	0.95 (0.85-1.05)	0.29
WCRF Cup 2013	Beef	per 50g increase per day	1.15 (0.64-2.08)	0.64
Poorolajal 2014	BMI PrMp	Overweight vs normal	1.21 (0.96-1.53)	0.11
Poorolajal 2014	BMI PoMp	Overweight vs normal	1.03 (0.87-1.21)	0.74
Poorolajal 2014	BMI	Overweight vs normal	1.08 (0.97-1.19)	0.17
WCRF Cup 2013	BMI PrMp	per 5kg/m2 increase	1.10 (0.99-1.22)	0.09
WCRF Cup 2013	BMI PoMp	per 5kg/m2 increase	1.04 (1.00-1.09)	0.06
Olsen 2007	BMI	>25 (iya)	1.21 (0.88-1.67)	0.24
Aune 2015	Weight	per 5kg gain	1.02 (0.96-1.09)	0.50
Aune 2015	Waist circumference	per 10cm	1.06 (1.00-1.12)	0.05
Aune 2015	Waist to hip ratio	per 0.1 unit	1.00 (0.93-1.07)	0.92
Aune 2015	Hip circumference	per 10cm	1.04 (0.82-1.31)	0.78
Li 2014	Breastfeeding	Ever vs never	0.89 (0.78-1.01)	0.08
WCRF Cup 2013	Cabbage	per 5g increase per day	1.00 (0.95-1.06)	0.90
WCRF Cup 2013	Coffee	per 200ml/day	1.02 (0.98-1.06)	0.27
Li 2017	CRP	High vs low	1.38 (0.88-2.17)	0.16
Han 2014	Cruciferous vegetables	Any vs none	0.97 (0.86-1.11)	0.70
WCRF Cup 2013	Dairy products	per 200g per day	1.06 (0.92-1.23)	0.41
WCRF Cup 2013	Cheese	per 50g per day	1.00 (0.81-1.25)	0.94
WCRF Cup 2013	Whole milk	per 200g per day	1.03 (0.87-1.23)	0.69
WCRF Cup 2013	Yoghurt	per 200g per day	1.02 (0.95-1.09)	0.55
WCRF Cup 2013	Lactose	Per 10g/day increase	1.03 (0.94-1.13)	0.54
Larsson 2006	Total milk	Highest vs lowest	1.40 (0.99-1.98)	0.06
Larsson 2006	Whole milk	Highest vs lowest	1.17 (0.81-1.68)	0.40
Larsson 2006	Yoghurt	Highest vs lowest	0.95 (0.69-1.30)	0.73
Larsson 2006	Cheese	Highest vs lowest	1.04 (0.60-1.80)	0.88
WCRF Cup 2013	Total β- carotene	per 1000ug per day	1.02 (0.99-1.05)	0.15
WCRF Cup 2013	Total folate	per 50ug per day	1.00 (0.97-1.03)	0.88
WCRF Cup 2013	Dietary folate	per 50ug per day	0.96 (0.88-1.05)	0.38

WCRF Cup 2013	Dietary vitamin A	per 2000IU per day	0.99 (0.95-1.03)	0.52
WCRF Cup 2013	Total vitamin C	per 200ug per day	1.03 (0.98-1.08)	0.19
WCRF Cup 2013	Dietary vitamin C	per 25mg per day	1.00 (0.97-1.03)	0.99
WCRF Cup 2013	Total vitamin E	per 50mg per day	1.00 (0.98-1.03)	0.60
WCRF Cup 2013	Dietary vitamin E	per 10g per day	1.05 (0.93-1.20)	0.49
Keum 2015	Egg consumption	per 5 eggs increase/week	1.69 (0.70-4.02)	0.23
Zeng 2015	Egg consumption	Highest vs lowest	1.21 (0.95-1.54)	0.13
WCRF Cup 2013	Egg consumption	per 25g increase per day	1.13 (0.89-1.44)	0.31
Qiu 2016	Total fat	Highest vs lowest	1.10 (0.97-1.24)	0.12
Qiu 2016	Animal fat	Highest vs lowest	1.09 (0.93-1.27)	0.28
Qiu 2016	Dairy fat	Highest vs lowest	1.10 (0.94-1.28)	0.24
Qiu 2016	Monounsaturated fat	Highest vs lowest	1.06 (0.95-1.19)	0.29
Qiu 2016	Plant fat	Highest vs lowest	0.93 (0.74-1.17)	0.54
Qiu 2016	Polyunsaturated fat	Highest vs lowest	1.06 (0.86-1.30)	0.57
Qiu 2016	Saturated fat	Highest vs lowest	1.06 (0.89-1.26)	0.52
WCRF Cup 2013	Saturated fat	per 5g per day	1.00 (0.91-1.12)	0.87
WCRF Cup 2013	Total fat	per 10g per day	1.03 (0.97-1.08)	0.31
WCRF Cup 2013	Vegetable fat	per 5g per day	1.00 (0.97-1.02)	0.78
Qiu 2016	Transaturated fats	Highest vs lowest	1.24 (0.85-1.90)	0.26
WCRF Cup 2013	Fibre	per 5g increase per day	0.94 (0.84-1.05)	0.26
WCRF Cup 2013	Fish	Per 25g increase/day	1.02 (0.90-1.13)	0.83
Jiang 2014	Fish	Any vs none; cohort	1.04 (0.89-1.22)	0.60
WCRF Cup 2013	Fruit	per 100g per day	1.06 (0.98-1.16)	0.15
WCRF Cup 2013	Total fruit and non-starchy vegetables	per 100g per day	1.01 (0.98-1.04)	0.46
Coll Gr 2015	HRT	Ever vs never; retrospective studies	0.83 (0.54-1.30)	0.42
Garg 1998	HRT	Ever vs never; invasive	1.15 (0.94-1.41)	0.18
Garg 1998	HRT	<1year duration; invasive	1.10 (0.79-1.54)	0.58
Garg 1998	HRT	Ever vs never; invasive and borderline	1.15 (0.94-1.41)	0.18
Zhou 2008	HRT	Former vs never; cohort	1.03 (0.95-1.13)	0.44
WCRF Cup 2013	Milk	per 200g per day	1.02 (0.95-1.09)	0.55
Koushik 2006	Dietary α-carotene	per 600ug per day	1.00 (0.95-1.05)	0.97
Koushik 2006	Dietary β-carotene	per 2500ug per day	0.98 (0.93-1.03)	0.41
Koushik 2006	Dietary β- cryptoxanthin	per 100ug per day	1.00 (0.97-1.02)	0.79
Koushik 2006	Dietary lycopene	per 4000ug per day	1.02 (0.98-1.06)	0.41
Koushik 2006	Dietary lutein/ zeaxanthine	per 2500ug per day	0.99 (0.94-1.03)	0.51
Huncharek 2001	Dietary β-carotene	Highest vs lowest	0.91 (0.53-1.56)	0.73
Li 2014	Dietary lycopene	Highest vs lowest	0.87 (0.92-1.23)	0.44
Kolahdooz 2010	Total meat	Highest vs lowest	1.08 (0.54-2.18)	0.83
Kolahdooz 2010	Red meat	Highest vs lowest	1.17 (0.97-1.42)	0.10
Kolahdooz 2010	Poultry	Highest vs lowest	1.03 (0.84-1.27)	0.77
Kolahdooz 2010	Fish	Highest vs lowest	1.00 (0.76-1.34)	0.95

WCRF Cup 2013	Monounsaturated fat	per 5g per day	0.97 (0.88-1.06)	0.48
Wallin 2011	Processed meat	per 100g increase/week	1.06 (0.98-1.14)	0.17
WCRF Cup 2013	Processed meat	per 50g increase per day	1.13 (0.88-1.46)	0.34
WCRF Cup 2013	Red meat	per 100g increase per day	1.03 (0.87-1.24)	0.71
WCRF Cup 2013	Poultry	per 25g increase per day	1.00 (0.91-1.10)	1.00
Murphy 2012	Aspirin	Regular v non regular; >1 per week	1.00 (0.87-1.17)	0.90
Bonovas 2006	Acetominophen	Ever vs never	0.97 (0.85-1.10)	0.66
Qu 2014	Total phytoestrogen intake	Highest vs lowest	0.77 (0.53-1.10)	0.15
Qu 2014	Isoflavones	Highest vs lowest	0.85 (0.38-1.88)	0.69
Qu 2014	Daidzein	Highest vs lowest	0.75 (0.48-1.15)	0.19
Qu 2014	Genistein	Highest vs lowest	0.65 (0.42-1.01)	0.60
Qu 2014	Quercetin	Highest vs lowest	0.80 (0.55-1.16)	0.24
Qu 2014	Ligans	Highest vs lowest	1.04 (0.63-1.72)	0.88
Qu 2014	Soyfood	Highest vs lowest	0.77 (0.57-1.04)	0.09
Qu 2014	Non soy food	Highest vs lowest	0.89 (0.55-1.44)	0.62
Qu 2014	Tofu	Highest vs lowest	0.72 (0.47-1.11)	0.13
Qu 2014	Non asian polyphenols	Highest vs lowest	0.79 (0.52-1.19)	0.26
Qu 2014	Asian polyphenols	Highest vs lowest	0.61 (0.26-1.44)	0.26
Zhou 2014	Recreational activity	Highest vs lowest	1.12 (0.88-1.42)	0.36
WCRF Cup 2013	Leisure- time physical activity	per 20 MET- hours per week	1.05 (0.97-1.14)	0.19
Zhong 2014	Non occupational physical activity	Moderate/high vs low	1.03 (0.87-1.20)	0.76
Zhong 2014	Non occupational physical activity	Moderate vs low	0.97 (0.86-1.10)	0.61
Zhong 2014	Non occupational physical activity	High vs low	0.97 (0.74-1.28)	0.84
Gong 2013	Age at menarche	Oldest vs youngest	0.89 (0.76-1.03)	0.11
Siristatidis 2013	IVF	Ever vs never (ref group infertile population, excl OC Dx <1yr post Rx)	1.26 (0.62-2.54)	0.52
Siristatidis 2013	IVF	Ever vs never (ref group gen population, total follow up)	1.42 (0.74-2.76)	0.29
Park 2016	Parity	Nullip vs parous	0.87 (0.64-1.18)	0.37
Schmid 2014	Sedentary behaviour	Highest vs lowest	1.28 (0.85-1.92)	0.24
Lee 2016	Environmental tobacco smoke	Any vs none	0.96 (0.77-1.19)	0.70
Liu 2014	Statin	Any vs none	0.81 (0.65-1.01)	0.06
Liu 2014	Statin	Any vs none (long term use)	0.51 (0.26-1.01)	0.05
Berge 2017	Genital talc use	Ever vs never	1.05 (0.91-1.22)	0.5
WCRF Cup 2013	Tea	per 200ml per day	0.96 (0.91-1.00)	0.08
Zhan 2017	Tea	Highest vs lowest	0.84 (0.68-1.03)	0.09
Song 2017	Dietary calcium and supplemental	Highest vs lowest	1.11 (0.84-1.46)	0.46
Song 2017	Dairy calcium	Highest vs lowest	0.84 (0.69-1.01)	0.06
Xu 2018	Fibre	Highest vs lowest	0.97 (0.85-1.12)	0.71
Penninkilampi 2018	Perineal talc	Long term (>10yr) vs none	0.98 (0.75-1.29)	0.88
Wang 2018	Dietary pattern; healthy	Highest vs lowest	0.95 (0.85-1.06)	0.35
Wang 2018	Dietary pattern; western	Highest vs lowest	1.22 (0.91-1.65)	0.18

WCRF Cup 2013	Beer	per 10g increase per day	1.06 (0.60-1.88)	0.83
Zhang X 2018	Bisphosphonates	Any vs none	0.64 (0.40-1.03)	0.06
Zhang X 2018	Bisphosphonates	>1 yr of use vs none	0.88 (0.45-1.72)	0.71
Li 2019	CRP	>10mg/L vs <10mg/L	2.02 (0.96-4.26)	0.06
Pang 2018	Dietary protein intake	Highest vs lowest	0.90 (0.68-1.20)	0.48
Zheng 2018	Dietary fibre intake	per 10g increase per day	0.93 (0.80-1.08)	0.33
Salari-Moghaddam 2019	Coffee	Highest vs lowest; cohort	1.10 (0.90-1.35)	0.35
Salari-Moghaddam 2019	Caffeine	Highest vs lowest; cohort	0.91 (0.69-1.19)	0.49
Salari-Moghaddam 2019	Caffeinated coffee	Highest vs lowest	0.98 (0.65-1.49)	0.93
Salari-Moghaddam 2019	Decaffeinated coffee	Highest vs lowest; cohort	0.87 (0.67-1.14)	0.31
Santucci 2019	Cigarette smoking	Current vs never	1.15 (0.99-1.33)	0.07
Wen 2019	Metformin	Ever vs never (PY)	0.38 (0.09-1.50)	0.17
Wu 2019	Age at last birth	Highest vs lowest	0.92 (0.85-1.00)	0.05
Collaborative Group 2015	HRT	Ever vs never; info duration of use and times since last use	0.87 (0.52-1.47)	0.61
Collaborative Group 2015	HRT	Current/recent use vs never	0.75 (0.41-1.36)	0.34

Abbreviations: BMI, body mass index; cc, case control; CI, confidence interval; cm, centimetres; CRP, C reactive protein; Dx, diagnosis; excl, excluding; g, grams; HRT, hormone replacement therapy; IL6, interleukin 6; IU, international units; IVF, in vitro fertilisaiton; inf, infertile; iya, in young adulthood; kg, kilograms; m2, metre squared; MET, metabolic equivalent of task; ml, millilitres; ng, nanograms; nmol, nanomole; NSAIDS, non-steroidal anti-inflammatory drugs; OC, ovarian cancer; PCOS, polycystic ovarian syndrome; PrMp, premenopausal; PoMp, postmenopausal; ref, reference; RR, relative risk; RX, treatment; TNFα, tumour necrosis factor α; TNFR2, tumour necrosis factor receptor 2; ug, micrograms; WCRF, World Cancer Research Fund

Supplementary Table S3: Results with random p value >0.05, not statistically significant, all studies

Author, year	Exposure	Exposure contrast	RR (95% CI)	p value
Pelucchi 2015	Acylamide	High v Low	1.12 (0.85-1.47)	0.43
Pelucchi 2015	Acylamide	10ug/day increase	1.02 (0.96-1.09)	0.47
Yan -Hong 2015	Alcohol	Any vs none	1.03 (0.96-1.10)	0.47
Yan -Hong 2015	Alcohol	Low vs none	0.96 (0.93-1.00)	0.96
Yan -Hong 2015	Alcohol	Moderate vs none	1.08 (0.92-1.27)	0.33
Yan -Hong 2015	Alcohol	High vs none	0.99 (0.88-1.12)	0.90
Kim 2010	Wine	Ever vs never	1.13 (0.92-1.28)	0.24
Kim 2010	Wine	Former vs never	1.12 (0.87-1.44)	0.37
Kim 2010	Wine	Current vs never	1.12 (0.87-1.44)	0.37
Kim 2010	Wine	Current vs former	0.74 (0.41-1.34)	0.32
WCRF Cup 2013	Wine	per 10g increase per day	1.07 (0.88-1.29)	0.49
Zhang 2016	Aspirin	Ever vs never; cohort	0.95 (0.85-1.05)	0.29

Murphy 2012	Aspirin	regular v non regular	0.96 (0.85-1.08)	0.49
Bonovas 2006	Acetominophen (paracetamol)	Ever vs never	0.84 (0.70-1.00)	0.06
WCRF Cup 2013	Beef	per 50g increase per day	1.15 (0.85-1.56)	0.36
Pooralajal 2014	BMI PrMp	Normal vs overweight	1.21 (0.96-1.53)	0.11
Pooralajal 2014	BMI PoMp	Normal vs overweight	1.03 (0.87-1.21)	0.74
WCRF Cup 2013	BMI PrMp	per 5kg/m^2 increase	1.10 (0.99-1.22)	0.09
WCRF Cup 2013	BMI PoMp	per 5kg/m^2 increase	1.04 (1.00-1.09)	0.06
Olsen 2007	BMI	>25 (iya)	1.21 (0.88-1.67)	0.24
Aune 2015	Weight	per 5kg gain	1.02 (0.96-1.09)	0.50
Aune 2015	Waist circumference	per 10cm	1.06 (1.00-1.12)	0.05
Aune 2015	Waist to hip ratio	per 0.1 unit	1.00 (0.93-1.07)	0.92
Aune 2015	Hip circumference	per 10cm	1.04 (0.82-1.31)	1.31
Ip 2007	Breastfeeding	<12months vs never	0.95 (0.80-1.12)	0.52
WCRF Cup 2013	Cabbage	per 5g increase per day	1.00 (0.95-1.06)	0.90
Yin 2011	Circulating Vitamin D	per 20ng/ml increase	0.83 (0.63-1.08)	0.17
WCRF Cup 2013	Coffee	per 200ml/day	1.02 (0.98-1.06)	0.27
Li 2017	CRP	Middle vs lowest	1.06 (0.91-1.25)	0.46
WCRF Cup 2013	Dairy products	per 200g per day	1.06 (0.92-1.23)	0.41
WCRF Cup 2013	Cheese	per 50g per day	1.00 (0.81-1.25)	0.94
WCRF Cup 2013	Whole milk	per 200g per day	1.03 (0.87-1.27)	0.69
WCRF Cup 2013	Yoghurt	per 200g per day	1.02 (0.95-1.09)	0.55
WCRF Cup 2013	Milk	Per 200g/day increase	1.02 (0.95-1.09)	0.55
WCRF Cup 2013	Lactose	Per 10g/day increase	1.03 (0.94-1.13)	0.55
Larsson 2006	Total dairy products	Highest vs lowest	1.17 (0.85-1.60)	0.34
Larsson 2006	Total milk	Highest vs lowest	0.87 (0.68-1.10)	0.24
Larsson 2006	Skim/low milk	Highest vs lowest	0.94 (0.75-1.17)	0.57
Larsson 2006	Yoghurt	Highest vs lowest	1.13 (0.96-1.33)	0.14
Larsson 2006	Cheese	Highest vs lowest	0.95 (0.80-1.12)	0.54
Larsson 2006	Lactose	Highest vs lowest	1.01 (0.85-1.21)	0.88
WCRF Cup 2013	Total β- carotene	per 1000ug per day	1.02 (1.00-1.05)	0.15
WCRF Cup 2013	Total folate	per 50ug per day	1.00 (0.97-1.03)	0.88
WCRF Cup 2013	Dietary folate	per 50ug per day	0.96 (0.88-1.05)	0.38
WCRF Cup 2013	Dietary vitamin A	per 2000IU per day	0.99 (0.95-1.03)	0.52
WCRF Cup 2013	Total vitamin C	per 200ug per day	1.03 (0.98-1.08)	0.19
WCRF Cup 2013	Dietary vitamin C	per 25mg per day	1.00 (0.97-1.03)	0.99
WCRF Cup 2013	Total vitamin E	per 50mg per day	1.01 (0.98-1.03)	0.61
WCRF Cup 2013	Dietary vitamin E	per 10g per day	1.05 (0.92-1.20)	0.49
Keum 2015	Egg consumption	per 5 eggs increase per week	1.09 (0.97-1.22)	0.15
WCRF Cup 2013	Egg consumption	per 25g increase per day	1.13 (0.89-1.44)	0.31
Qiu 2016	Animal fat	Highest vs lowest	1.20 (0.99-1.47)	0.70
Qiu 2016	Dairy fat	Highest vs lowest	1.05 (0.92-1.19)	0.48

Qiu 2016	Monounsaturated fat	Highest vs lowest	1.00 (0.90-1.10)	0.92
Qiu 2016	Plant fat	Highest vs lowest	0.95 (0.83-1.09)	0.47
Qiu 2016	Polyunsaturated fat	Highest vs lowest	0.97 (0.86-1.10)	0.65
Qiu 2016	Saturated fat	Highest vs lowest	1.10 (0.99-1.21)	0.07
WCRF Cup 2013	Saturated fat	per 5g per day	1.07 (0.96-1.20)	0.24
WCRF Cup 2013	Total fat	per 10g per day	1.03 (0.99-1.07)	0.17
WCRF Cup 2013	Vegetable fat	per 5g per day	0.99 (0.97-1.02)	0.64
WCRF Cup 2013	Fibre	per 5g increase per day	0.94 (0.85-1.05)	0.26
WCRF Cup 2013	Fish	Per 25g increase/day	1.01 (0.90-1.13)	0.83
Jiang 2014	Fish	Any vs none; cc	0.90 (0.73-1.12)	0.36
Jiang 2014	Fish	Any vs none; cohort	1.04 (0.89-1.22)	0.60
WCRF Cup 2013	Fruit	per 100g per day	1.05 (0.98-1.12)	0.17
WCRF Cup 2013	Total fruit and non-starchy vegetables	per 100g per day	1.01 (0.98-1.04)	0.50
Coll Gr 2015	HRT	Ever vs never; retrospective studies	1.02 (0.93-1.11)	0.72
Garg 1998	HRT	<1 year duration; invasive cancer	1.12 (0.91-1.37)	0.27
Garg 1998	HRT	< 1 year duration; all	1.13 (0.93-1.38)	0.22
Zhou 2008	HRT	Former vs never; cohort	1.03 (0.95-1.13)	0.44
Wang 2016	Hysterectomy	Ever vs never	0.97 (0.81-1.15)	0.69
WCRF Cup 2013	Milk	per 200g per day	1.03 (0.87-1.27)	0.69
Koushik 2006	Dietary α-carotene	per 600ug per day	1.00 (0.95-1.05)	0.97
Koushik 2006	Dietary β-carotene	per 2500ug per day	0.98 (0.93-1.03)	0.41
Koushik 2006	Dietary β- cryptoxanthin	per 100ug per day	1.00 (0.97-1.02)	0.79
Koushik 2006	Dietary lycopene	per 4000ug per day	1.02 (0.98-1.06)	0.41
Koushik 2006	Dietary lutein/ zeaxanthine	per 2500ug per day	0.99 (0.94-1.03)	0.51
Huncharek 2001	Dietary β-carotene	Highest vs lowest	0.76 (0.58-1.01)	0.61
Li 2014	Dietary lycopene	Highest vs lowest	0.96 (0.86-1.08)	0.52
Kolahdooz 2010	Total meat	Highest vs lowest	1.16 (0.96-1.40)	0.13
Kolahdooz 2010	Poultry	Highest vs lowest	0.90 (0.79-1.02)	0.09
Kolahdooz 2010	Fish	Highest vs lowest	0.84 (0.68-1.02)	0.09
WCRF Cup 2013	Monounsaturated fat	per 5g per day	0.97 (0.88-1.06)	0.48
Wallin 2011	Processed meat	per 100g increase per week	1.06 (0.98-1.14)	0.17
WCRF Cup 2013	Processed meat	per 50g increase per day	1.13 (0.88-1.46)	0.34
WCRF Cup 2013	Red meat	per 100g increase per day	1.03 (0.87-1.24)	0.71
WCRF Cup 2013	Poultry	per 25g increase per day	1.00 (0.90-1.10)	1.00
Baandrup 2013	NSAIDS	Ever vs never; non aspirin	0.94 (0.84-1.06)	0.30
Barry 2014	PCOS	Yes vs no	1.50 (0.95-2.37)	0.09
Qu 2014	Genistein	Highest vs lowest	0.65 (0.42-1.01)	0.56
Qu 2014	Quercetin	Highest vs lowest	0.92 (0.69-1.23)	0.58
Qu 2014	Kaempferol	Highest vs lowest	0.77 (0.55-1.08)	0.13
Qu 2014	Flavones	Highest vs lowest	0.82 (0.66-1.02)	0.07
Qu 2014	Ligans	Highest vs lowest	0.83 (0.50-1.40)	0.49

Qu 2014	Soyfood	Highest vs lowest	0.77 (0.57-1.04)	0.09
Qu 2014	Non soy food	Highest vs lowest	0.86 (0.65-1.14)	0.30
Qu 2014	Tofu	Highest vs lowest	0.72 (0.47-1.11)	0.13
Qu 2014	Non asian polyphenols	Highest vs lowest	0.79 (0.52-1.19)	0.26
Zhou 2014	Recreational activity	High vs low	0.90 (0.71-1.12)	0.34
WCRF Cup 2013	Leisure- time physical activity	per 20 MET- hours per week	1.05 (0.97-1.14)	0.19
Zhong 2014	Non occupational physical activity	Moderate/high vs low	0.92 (0.84-1.00)	0.05
Zhong 2014	Non occupational physical activity	Moderate/high vs low	1.03 (0.87-1.20)	0.76
Zhong 2014	Non occupational physical activity	High vs low	0.89 (0.79-1.00)	0.06
Siristatidis 2013	IVF	Ever vs never (ref group inf population, excl OC Dx <1yr post Rx)	1.26 (0.62-2.54)	0.52
Siristatidis 2013	IVF	Ever vs never (ref group gen population, total follow up)	1.42 (0.74-2.76)	0.29
Negri 1991	Abortions	1 vs none	0.92 (0.74-1.15)	0.46
Park 2016	Parity	Nullip vs parous	0.80 (0.61-1.05)	0.12
Schmid 2014	Sedentary behaviour	Highest vs lowest	1.22 (0.97-1.70)	0.25
WCRF Cup 2013	Serum vitamin D	Highest vs lowest	0.91 (0.63-1.32)	0.63
WCRF Cup 2013	Serum vitamin D	Per 10 nmol/L	1.00 (0.87-1.17)	0.90
Lee 2016	Environmental tobacco smoke	Any vs none	0.91 (0.76-1.09)	0.29
Zeng 2016	IL6	Highest vs lowest	1.15 (0.89-1.00)	0.29
Zeng 2016	TNFα	Highest vs lowest	1.54 (0.88-2.68)	0.13
Zeng 2016	TNFR2	Highest vs lowest	1.35 (0.88-2.07)	0.17
Liu 2014	Recreational activity	High vs low	0.90 (0.72-1.12)	0.34
Huncharek 2007	Talc on contraceptive diaphragm	Any vs none	1.00 (0.77-1.29)	0.99
WCRF Cup 2013	Tea	per 200ml per day	0.96 (0.91-1.00)	0.08
Butler 2011	Black tea	Highest vs lowest	0.88 (0.76-1.02)	0.09
Gao 2013	Tea; green	1 cup/d v none	0.82 (0.61-1.10)	0.18
Song 2017	Dietary calcium and supplemental	Highest vs lowest	0.90 (0.65-1.24)	0.53
Wang 2018	Alcohol; heavy drinking	Highest vs lowest	0.89 (0.67-1.19)	0.43
WCRF Cup 2013	Beer	per 10g increase per day	1.06 (0.60-1.88)	0.83
Zhang X 2018	Bisphosphonates	<1 yr of use vs none	0.95 (0.74-1.21)	0.66
Zhang X 2018	Bisphosphonates	>1 yr of use vs none	0.87 (0.67-1.14)	0.31
Huo 2018	Antidepressants	Ever vs never	1.10 (0.91-1.32)	0.31
Salari-Moghaddam 2019	Coffee	Highest vs lowest; cohort	1.10 (0.90-1.35)	0.35
Huo 2018	Antidepressants	per 1 year increment	0.99 (0.94-1.05)	0.78
Salari-Moghaddam 2019	Caffeine	Highest vs lowest; cohort	0.90 (0.69-1.19)	0.49
Salari-Moghaddam 2019	Caffeinated coffee	Highest vs lowest	0.98 (0.65-1.49)	0.93
Salari-Moghaddam 2019	Decaffeinated coffee	Highest vs lowest; cohort	0.87 (0.67-1.14)	0.31
Santucci 2019	Cigarette smoking	Current vs never	1.05 (0.95-1.16)	0.30
Wen 2019	Metformin	Ever vs never	0.38 (0.09-1.50)	0.17
Coll Gr 2015	HRT	Ever vs never; info duration of use and times since last use	1.03 (0.94-1.15)	0.45
Coll Gr 2015	HRT	Current/recent use vs never	1.04 (0.93-1.17)	0.51
Shafiei 2019	Coffee	Ever vs never; cc	1.09 (0.93-1.26)	0.28

Shafiei 2019	Caffeine	Ever vs never; cc	0.89 (0.55-1.45)	0.65
Shafiei 2019	Caffeinated coffee	Ever vs never; cc	1.05 (0.87-1.28)	0.60

Abbreviations: BMI, body mass index; cc, case control; CI, confidence interval; cm, centimetres; CRP, C reactive protein; Dx, diagnosis; excl, excluding; g, grams; HRT, hormone replacement therapy; IL6, interleukin 6; IU, international units; IVF, in vitro fertilisaiton; inf, infertile; iya, in young adulthood; kg, kilograms; m2, metre squared; MET, metabolic equivalent of task; ml, millilitres; ng, nanograms; nmol, nanomole; NSAIDS, non-steroidal anti-inflammatory drugs; OC, ovarian cancer; PCOS, polycystic ovarian syndrome; PrMp, premenopausal; PoMp, postmenopausal; ref, reference; RR, relative risk; RX, treatment; TNFα, tumour necrosis factor α; TNFR2, tumour necrosis factor receptor 2; ug, micrograms; WCRF, World Cancer Research Fund

Supplementary Table S4: Description of 40 meta- analyses with statistically significant results investigating risk factors associated with ovarian cancer incidence or mortality –cohort studies.

Author, year	Exposure	Exposure contrast	N$^{\alpha}$	Sample size cases/ cohort	Summary relative risk (95% CI)			Fixed P-value$^{\varepsilon}$	Random P-value$^{\phi}$	95% Prediction interval$^{\gamma}$
					Fixed Effects$^{\beta}$	Random Effects$^{\chi}$	Largest Study$^{\delta}$			
**Anthropometric indices**										
Pooralajal 2014	BMI	BMI ≥30kg/m^2 vs normal, PrMP	3	71/ 350211	1.57 (1.20-2.06)	1.57 (1.20-2.06)	1.56 (1.14-2.16)	9.7E-04	9.7E-04	0.27-9.02
Pooralajal 2014	BMI	BMI ≥30kg/m^2 vs normal	13	6947/20, 560, 388[e]	1.27 (1.17-1.36)	1.27 (1.17-1.38)	1.27 (1.19-1.36)	2.8E-16	2.6E-8	1.09-1.47
Aune 2015	BMI	per 5kg/m2 increase	24	17734/ 16300000	1.06 (1.03-1.08)	1.07 (1.04-1.11)	0.97 (0.93-1.01)	2.9E-0.7	1.2E-04	0.96-1.21
Aune 2015	BMI	iya per 5kg/m2 increase	6	9452/ 11100000	1.12 (1.05-1.19)	1.12 (1.05-1.19)	1.16 (1.04-1.29)	3.8E-04	3.8E-04	1.03-1.23
Aune 2015	Weight	Per 5kg	4	1006/ 297350	1.03 (1.01-1.05)	1.03 (1.01-1.05)	1.02 (1.00-1.05)	8.0E-04	1.4E-03	0.98-1.08
Aune 2015	Height	Per 10cm	16	18663/ 13600000	1.14 (1.12-1.18)	1.16 (1.11-1.20)	1.14 (1.10-1.18)	1.17E-26	2.20E-13	1.06-1.26
Keum 2015	Weight gain	Per 5kg; never HRT (PoMP)	2	217/ 23984	1.13 (1.03-1.24)	1.13 (1.03-1.24)	1.16 (1.03-1.31)	8.5E-03	8.5E-03	NA
**Dietary intake**										
Song 2017	Calcium intake	HvL	5	1726/ 351192	0.86 (0.74-1.00)	0.86 (0.74-1.00)	0.86 (0.68-1.10)	4.00E-02	4.0E-02	0.67-1.09
Larsson 2006	Dairy	HvL, total dairy products	2	427/ 90001	1.66 (1.19-2.31)	1.66 (1.19-2.31)	1.61 (1.07-2.42)	2.8E-03	2.8E-03	NA
Larsson 2006	Dairy	HvL, skim/low fat	3	728/ 170327	1.35 (1.09-1.68)	1.35 (1.09-1.68)	1.32 (0.97-1.82)	5.7E-03	5.7E-03	0.35-5.43
Larsson 2006	Dairy	HvL, lactose	3	728/ 170327	1.47 (1.17-1.84)	1.47 (1.17-1.84)	1.48 (1.05-2.09)	7.8E-04	7.8E-04	0.34-6.29
Kolahdooz 2010	Meat	HvL, processed	3	1018/ 696100	1.26 (1.02-1.56)	1.26 (1.02-1.56)	1.23 (0.92-1.63)	3.5E-02	3.5E-02	0.31-5.07
Wallin 2011	Meat	Per 100g/wk increment; red and processed	21	6536/ 2140286	1.01 (1.00-1.04)	1.01 (1.00-1.04)	1.02 (0.98-1.06)	3.4E-02	3.4E-02	1.00-1.04
WCRF CUP 2013	Non starchy vegetables	Per 100g/day	6	2053/ 641079	0.95 (0.90-0.99)	0.94 (0.89-1.00)	1.00 (0.93-1.07)	2.8E-02	4.0E-02	0.82-1.08
Zhang 2018	Non herbal tea	HvL	3	734/ 164882	0.69 (0.52-0.93)	0.69 (0.52-0.93)	0.63 (0.40-0.99)	1.4E-02	1.4E-02	0.11-4.57
Butler 2011	Tea; black	HvL	5	1299/ 203998	0.72 (0.57-0.91)	0.73 (0.56-0.93)	0.63 (0.40-0.99)	5.4E-03	1.2E-02	0.42-1.24
**Medical history**										
Zhang 2017	Diabetes Mellitus	DM vs no DM	17	5036/ 2868215	1.22 (1.16-1.27)	1.32 (1.14-1.52)	1.23 (1.15-1.32)	1.4E-16	1.7E-04	0.81-2.15
Bernatsky 2011	SLE	Observed vs expected	4	44/40855	0.73 (0.53-1.00)	0.73 (0.53-1.00)	0.82 (0.54-1.20)	4.9E-01	4.9E-02	0.36-1.46
Li 2019	Endometriosis	Any vs none	25	1921/475988	1.58 (1.44-1.73)	2.35 (1.77-3.12)	1.34 (1.16-1.55)	8.82E-24	3.10E-09	0.87-6.34
**Use of medical/ hormonal therapy**										
Zhou 2008	HRT	Current vs ever	5	3958/ 1342899	1.25 (1.15-1.36)	1.28 (1.15-1.42)	1.20 (1.09-1.32)	8.5E-08	6.5E-06	1.01-1.62

Shi 2015	HRT	Ever vs never (cont E+P)	4	3337/ 1265735	1.22 (1.06-1.40)	1.22 (1.06-1.40)	1.13 (0.96-1.34)	5.8E-03	5.8E-04	0.90-1.65
Shi 2015	HRT	Ever vs never (seq E+P)	4	3337/ 1265735	1.23 (1.08-1.40)	1.35 (1.06-1.72)	1.14 (0.98-1.32)	1.4E-03	1.5E-02	0.54-3.35
Shi 2015	HRT	Ever vs never ET only	9	7512/ 2302683	1.40 (1.28-1.54)	1.44 (1.25-1.66)	1.31 (1.11-1.54)	2.8E-13	7.1E-07	0.99-2.09
Shi 2015	HRT	Ever vs never ET+PT	9	7512/ 2302683	1.28 (1.19-1.38)	1.23 (1.08-1.14)	1.50 (1.34-1.68)	1.1E-11	2.3E-03	0.87-1.75
Shi 2015	HRT	Ever vs never ET +E/PT	2	543/ 141880	1.55 (0.05-2.30)	1.55 (1.05-2.30)	1.50 (0.92-2.44)	2.7E-02	2.7E-02	NA
Coll Group 2015	HRT; Prospective	Current/recent vs never	11	11664/948390	1.37 (1.27-1.48)	1.37 (1.27-1.48)	1.28 (1.14-1.44)	1.3E-15	1.3E-15	1.26-1.50
Coll Group 2015	HRT; Prospective	Ever vs never	17	12110/950663	1.20 (1.13-1.28)	1.20 (1.13-1.28)	1.15 (1.06-1.26)	2.1E-09	2.1E-09	1.13-1.28
Coll Group 2015	HRT; Prospective	Ever vs never (info duration of use and times since last use)	14	11866/949657	1.24 (1.16-1.32)	1.24 (1.16-1.32)	1.16 (1.05-1.28)	5.2E-10	6.0E-10	1.15-1.33
Coll Group 2015	HRT; Prospective	Ever vs never	17	12110/950663	1.20 (1.13-1.28)	1.20 (1.13-1.28)	1.15 (1.06-1.26)	2.1E-09	2.1E-09	1.13-1.28
Coll Group 2008	OCP *	Ever vs never	45	7726/32201	0.74 (0.69-0.80)	0.74 (0.69-0.80)	0.74 (0.67-0.82)	5.7E-19	5.8E-16	0.68-0.81
Hankinson 1992	OCP	Ever vs never	3	60/ 80670	0.43 (0.25-0.75)	0.43 (0.25-0.75)	0.60 (0.30-1.40)	2.8E-03	3.0E-03	0.01-15.16
Baandrup 2013	NSAIDS; Non aspirin	Ever vs never	6	1782/ 505136	0.90 (0.81-1.00)	0.90 (0.81-1.00)	0.90 (0.75-1.08)	4.4E-02	4.4E-02	0.78-1.04
Wen 2019	Metformin	Ever vs never	3	3288/513702	0.16 (0.15-0.18)	0.18 (0.12-0.25)	0.16 (0.14-0.17)	0	2.5E-23	0.01-4.31
**Reproductive factors**										
Luan 2013	Breastfeeding	Per 5mo increase in duration	3	1180/ 447386	0.94 (0.90-0.99)	0.94 (0.89-1.00)	0.98 (0.92-1.05)	1.8E-02	3.4E-02	0.80-1.49
Siristatidis 2013	IVF	Ever vs never (reference group general population; excl OC diag <1yr post rx)	6	31606/ 1438001	1.50 (1.17-1.92)	1.47 (1.06-2.03)	1.30 (0.90-1.88)	1.2E-03	2.00E-02	0.73-2.96
Siristatidis 2013	IVF	Ever vs never (reference group IVF population; total follow up)	6	31606/ 1438001	1.62 (1.27-2.07)	1.66 (1.08-2.55)	1.35 (0.93-1.96)	1.0E-04	2.20E-02	0.52-5.28
Zhou 2017	PID	Ever vs never	6	8285/ 2929284	1.22 (1.11-1.34)	1.32 (1.05-1.66)	1.05 (0.92-1.20)	3.5E-05	1.62E-02	0.71-2.47
**Environmental factors**										
Reid 2011	Asbestos	Any vs none	14	5165/ 906145	1.77 (1.47-2.14)	1.86 (1.46-2.36)	1.30 (0.90-1.80)	2.4E-09	5.0E-07	1.05-3.29
Camargo 2011	Asbestos; Occupational	Total exposed vs nonexposed	20	126/ 21973	1.71 (1.42-2.06)	1.77 (1.37-2.27)	1.12 (0.66-1.80)	2.3E-08	9.7E-06	0.85-3.66
Camargo 2011	Asbestos; Occupational	High exposed vs nonexposed	6	20/ 6149	2.77 (1.69-4.54)	2.78 (1.36-5.66)	1.10 (0.37-2.21)	5.1E-05	5E-03	0.42-18.44

Abbreviations: BMI, body mass index; BMI iya, Body mass index in young adulthood; CC, case control; CI, confidence interval; CRP, C-reactive protein; cont E+P, continuous estrogen and progesterone; seq E+P, sequential estrogen and progesterone; ET, estrogen therapy; PT, progesterone therapy; E/PT, estrogen/ progesterone therapy; HvL, highest versus lowest; HR, hazard ratio; HRT, hormone replacement therapy; IVF, in vitro fertilisation; NA: Not available, due to <3 included studies; NSAID, non-steroidal anti-inflammatory drug; OC, ovarian cancer; OCP, oral contraceptive pill; PID, pelvic inflammatory disease; PoMP, postmenopausal; PrMP, premenopausal; RR, relative risk; rx, treatment; SLE, systemic lupus erythematous; T1T2, Type 1 or Type 2 diabetes mellitus; WG, weight gain

Key:

[α] Number of studies

[β] Fixed effects refers to summary relative risk 95% CI) using the meta-analysis fixed- effects model

[χ] Random effects refers to summary relative risk (95% CI) using the meta-analysis random -effects model.

[δ] Relative risk and 95% confidence interval of largest study (smallest SE) in each meta- analysis

[ε] P value of summary fixed effects estimate

[ϕ] P value of summary random effects estimate

[γ] Prediction intervals are reported only for meta-analyses including at least 3 studies

[η] Person years

All statistical tests were two-sided

* %reduction in the standard error

Supplementary Table S5: Evaluation of heterogeneity, small study effects and excess significance bias in the 40 meta-analyses investigating the risk factors associated with ovarian cancer incidence or mortality[§] – only statistically significant cohort studies included.

Author, year	Exposure	Exposure contrast	Egger's P^{α}	I^2 (95% CI) P^{β}	Studies	Observed$^{\chi}$	Expected$^{\delta}$, P-value$^{\varepsilon}$					
							Fixed effects		Random effects		Largest study	
**Anthrometric indices**												
Pooralajal 2014	BMI	BMI ≥30kg/m^2 vs normal, PrMP	0.61	0 (0 -73) 0.86	3	1	0.68	0.66	0.68	0.66	0.66	0.64
Pooralajal 2014	BMI	BMI ≥30kg/m^2 vs normal	0.88	12 (0-54) 0.33	13	3	5.20	NP	5.20	NP	5.30	NP
Aune 2015	BMI	per 5kg/m2 increase	0.07	48 (6-67) 0.00	12	5	2.93	0.55	3.86	0.23	1.80	<0.01
Aune 2015	BMI	iya per 5kg/m2 increase	0.60	0 (0-61) 0.56	24	6	2.00	NP	2.00	NP	2.48	NP
Aune 2015	Weight	Per 5kg	0.42	7 (0-70) 0.36	6	1	0.23	0.10	0.24	0.10	0.21	0.08
Aune 2015	Height	Per 10cm	0.18	27 (0-59) 0.15	4	1	6.51	NP	6.86	NP	6.22	NP
Keum 2015	Weight gain	Per 5kg; never HRT (PoMP)	NA	NA	16	9	0.21	0.69	0.21	0.69	0.27	0.13
**Dietary intake**												
Song 2017	Calcium intake	HvL	0.62	0 (0-64) 0.61	5	0	1.73	NP	1.73	NP	1.66	NP
Larsson 2006	Dairy	HvL, total dairy products	NA	NA	2	1	1.91	NP	1.91	NP	1.87	NP
Larsson 2006	Dairy	HvL, skim/low fat	0.21	0 (0-73) 0.64	3	0	2.14	NP	2.14	NP	1.93	NP
Larsson 2006	Dairy	HvL, lactose	0.42	0 (0-73) 0.92	3	1	2.61	NP	2.61	NP	2.64	NP
Kolahdooz 2010	Meat	HvL, processed	0.37	0 (0-73) 0.93	3	0	1.79	NP	1.79	NP	1.56	NP
Wallin 2011	Meat	Per 100g/wk increment; red and processed	0.11	0 (0-41) 1.00	21	0	1.13	NP	1.13	NP	1.14	NP
WCRF CUP 2013	Non starchy vegetables	Per 100g/day	0.21	28 (0-71) 0.23	6	1	0.49	0.44	0.58	0.57	0.30	0.19
Butler 2011	Tea; black	HvL	0.44	15 (0-69) 0.32	5	2	3.88	NP	3.84	NP	4.71	NP
Zhang 2018	Non herbal tea	HvL	0.03	0 (0-73) 0.53	3	1	2.44	NP	2.44	NP	2.74	<0.01
**Medical History**												
Zhang 2017	Diabetes Mellitus	DM vs no DM	0.30	80 (67-86) 0.00	17	6	6.33	NP	8.29	NP	6.71	NP
Bernatsky 2011	SLE	Observed vs expected	0.97	0 (0-68) 0.53	4	0	0.36	NP	0.36	NP	0.26	NP
Li 2019	Endometriosis	Any vs none	0.00	83 (73-89) 0.00	25	12	9.49	0.18	13.70	NP	5.70	<0.01
**Use of medical/ hormonal therapy**												
Zhou 2008	HRT	Current vs ever	0.08	14 (0-69) 0.33	5	3	3.39	NP	3.61	NP	2.91	0.93
Shi 2015	HRT	Ever vs never (cont E+P)	0.07	0 (0-68) 0.50	4	1	2.55	NP	2.55	NP	1.63	NP
Shi 2015	HRT	Ever vs never (seq E+P)	0.18	50 (0-82) 0.11	4	2	2.70	NP	3.53	NP	1.75	0.80
Shi 2015	HRT	Ever vs never ET only	0.71	48 (0-74) 0.54	9	6	8.04	NP	8.28	NP	7.01	NP
Shi 2015	HRT	Ever vs never ET+PT	0.40	53 (0-76) 0.03	9	3	6.55	NP	5.60	NP	8.56	NP
Shi 2015	HRT	Ever vs never ET +E/PT	NA	NA	2	0	1.94	NP	1.94	NP	1.89	NP
Collab Gr 2015	HRT; Prospective	Current/recent vs never	0.68	0 (0-50) 0.78	11	3	8.72	NP	8.72	NP	7.20	NP
Collab Gr 2015	HRT- Prospective	Ever vs never	0.71	0 (0-45) 0.81	17	4	6.27	NP	6.27	NP	4.90	NP
Collab Gr 2015	HRT- Prospective	Ever vs never (info duration of use and times since last use)	0.97	0 (0-48) 0.45	14	2	6.71	NP	6.72	NP	4.87	NP
Wen 2019	Metformin	Ever vs never	0.38	14 (0-77) 0.31	3	2	2.41	NP	2.38	NP	2.42	NP
Zhou 2008	HRT	Current vs ever	0.08	14 (0-69) 0.32	5	3	3.39	NP	3.61	NP	2.91	0.93
Hankinson 1992	OCP	Ever vs never	0.41	NA	22	1	1.58	NP	1.58	NP	0.73	0.71
Baandrup 2013	NSAIDS Non aspirin	Ever vs never	0.55	0 (061) 0.93	6	0	1.02	NP	1.02	NP	1.02	NP

Collab Gr 2008	OCP	Ever vs never	0.61	0 (0-56) 0.61	45	3	6.67	NP	6.67	NP	6.68	NP
**Reproductive factors**												
Luan 2013	Breastfeeding	Per 5mo increase in duration	0.59	22 (0-78) 0.28	3	1	0.28	0.16	0.30	0.18	0.17	0.04
Siristatidis 2013	IVF	Ever vs never (reference group general population; excl OC diag <1yr post rx)	0.64	65 (0-83) 0.01	6	2	1.96	NP	1.89	NP	1.55	NP
Siristatidis 2013	IVF	Ever vs never (reference group IVF population; total follow up)	0.91	23 (0-69) 0.26	6	1	2.22	NP	2.30	NP	1.64	0.74
Zhou 2017	Pelvic inflammatory disease	Ever vs never	0.35	NA	3	1	9.88	0.25	13.7	NP	6.42	<0.01
**Environmental factors**												
Reid 2011	Asbestos[ζ]	Any vs none	0.64	28 (0 -61) 0.15	14	4	2.75	0.40	2.94	0.49	1.87	0.10
Camargo 2011	Asbestos; Occupational[ζ]	Total exposed vs nonexposed	0.72	35 (0-61) 0.06	20	6	4.11	0.59	5.14	NP	2.67	<0.01
Camargo 2011	Asbestos; Occupational[ζ]	High exposed vs nonexposed	0.78	45 (0-77) 0.12	6	2	2.93	0.55	3.86	0.23	1.80	<0.01

Abbreviations: BMI, body mass index; BMI iya, Body mass index in young adulthood; CC, case control; CI, confidence interval; CRP, C-reactive protein; cont E+P, continuous estrogen and progesterone; seq E+P, sequential estrogen and progesterone; ET, estrogen therapy; PT, progesterone therapy; E/PT, estrogen/ progesterone therapy; HvL, highest versus lowest; HR, hazard ratio; HRT, hormone replacement therapy; IVF, in vitro fertilisation; NA: Not available, due to <3 included studies; NP, not pertinent (P0.05<); NSAID, non-steroidal anti-inflammatory drug; OC, ovarian cancer; PoMP, postmenopausal; PrMP, premenopausal; RR, relative risk; rx, treatment; SLE, systemic lupus erythematous; T1T2, Type 1 or Type 2 diabetes mellitus; WG, weight gain

Key:

[α] P-value from the Egger's regression asymmetry test (P<0.10)

[β] I^2 metric of inconsistency (95% confidence interval) and the P-value of the Q test

[χ] Observed number of statistically significant studies in each meta-analysis

[δ] Expected number of statistically significant studies using the point estimate of each meta-analysis (from fixed effect, random effect of largest study accordingly) as the plausible effect size

[ε] P value of the excess statistical significance test

[ζ] Risk of dying from ovarian cancer

All statistical tests were two-sided

Supplementary Table S6: A Measurement Tool to Assess Systematic Reviews 2 (AMSTAR 2) Summary quality assessment for all included systematic reviews

AMSTAR 2 Questions<br><br>Study Author, year	PICO	'A priori ' design and deviations justified	Study design	Literature search	Duplicate study selection review	Duplicate data extraction	Excluded studies	Description of included studies	Assess risk of bias	Funding	Statistical methods for meta-analysis	Impact of RoB from meta-analysis	RoB in individual studies in results	Heterogeneity	Small study bias	Conflict of interest	Score
**Aune 2015**	•	•	•	•	•	•	○	•	•	•	•	•	•	•	•	○	Low
**Baandrup 2013**	•	◎	•	○	○	•	○	•	•	•	•	○	•	•	•	•	Critically low
**Barry 2014**	•	•	•	•	•	•	○	•	•	•	•	○	•	•	•	•	Low
**Berge 2017**	•	•	•	•	•	•	○	•	•	○	•	•	•	•	•	•	Low
**Bonovas 2006**	•	◎	•	•	○	•	○	•	•	•	•	•	•	•	•	•	Low
**Butler 2011**	•	○	•	○	○	○	○	•	•	○	•	○	○	○	○	•	Critically low
**Camargo 2011**	•	◎	•	•	•	•	○	•	○	•	•	○	•	•	•	•	Critically low
**Collabor group 2008**	•	◎	•	•	○	○	○	•	◎	•	•	○	○	•	○	○	Critically low
**Collabor group 2015**	•	◎	•	•	•	•	○	•	•	•	•	•	•	•	○	○	Critically low
**Gao 2013**	•	○	•	◎	○	○	○	•	•	•	•	○	•	•	•	•	Critically low
**Garg 1998**	•	○	•	◎	○	○	○	•	○	○	•	○	○	•	•	○	Critically low
**Gong 2013**	•	•	•	◎	•	•	○	•	•	•	•	•	•	•	•	○	Low
**Han 2014**	•	◎	•	•	○	○	○	•	◎	○	•	•	•	•	•	○	Low
**Huncharek 2001**	•	•	○	•	○	•	◎	◎	•	○	•	•	•	•	•	•	Moderate
**Huncharek 2007**	•	○	○	◎	○	○	○	•	◎	•	•	○	•	•	○	○	Critically low
**Huo 2018**	•	◎	•	•	•	•	◎	•	•	○	•	•	•	•	•	○	Moderate
**Ip 2007**	•	◎	•	◎	○	○	○	•	•	•	•	○	•	•	•	○	Low
**Jiang 2014**	•	◎	•	•	•	•	◎	•	◎	•	•	•	•	•	•	•	High
**Keum 2015 a**	•	◎	•	◎	•	•	◎	•	•	○	•	○	•	•	•	○	Moderate
**Keum 2015 b**	•	•	•	◎	•	•	○	◎	•	○	•	•	•	•	•	•	Moderate
**Kim 2010**	•	○	•	◎	•	•	○	•	◎	○	•	○	○	•	•	•	Critically low
**Kolahdooz 2010**	•	◎	•	◎	○	○	○	•	•	•	•	•	•	•	•	•	Low
**Koushik 2006**	•	○	•	◎	○	○	○	◎	○	○	•	○	•	○	○	○	Critically low
**Larsson 2006**	•	○	•	◎	○	○	○	•	◎	•	•	○	•	•	•	○	Critically low
**Lee 2016**	•	◎	•	○	○	○	○	○	○	○	•	○	○	○	○	○	Critically low
**Li 2019**	•	◎	•	◎	•	•	◎	•	◎	○	•	○	•	•	•	•	High
**Li 2017**	•	◎	•	◎	•	•	◎	•	○	•	•	○	•	•	•	•	Low
**Li 2014 a**	•	◎	•	◎	○	•	◎	•	◎	•	•	•	•	•	•	○	Moderate
**Li 2014 b**	•	○	○	◎	○	•	•	•	○	○	○	•	•	•	•	•	Critically low
**Liu 2019**	•	◎	•	•	•	•	◎	•	•	•	•	•	•	•	•	•	High
**Liu 2014**	•	◎	•	•	○	•	○	•	•	○	•	•	•	•	•	•	Low
**Luan 2013**	•	○	•	◎	○	•	○	•	○	•	•	○	•	•	•	•	Critically low
**Murphy 2012**	•	○	○	○	○	○	○	•	○	○	•	○	•	•	○	○	Critically low
**Negri 1991**	○	○	○	○	○	○	○	○	○	○	○	○	○	•	○	○	Critically low
**Olsen 2007**	•	○	•	○	○	○	○	•	•	○	•	○	•	•	•	•	Critically low
**Pang 2018**	•	◎	•	◎	•	•	◎	•	•	•	•	•	•	•	•	•	High
**Park 2016**	○	○	○	○	○	○	○	○	○	○	•	○	○	○	○	○	Critically low
**Pelucchi 2015**	•	○	○	◎	○	○	○	•	○	•	•	○	○	○	○	•	Critically low
**Penninkilampi 2018**	•	•	•	◎	○	○	◎	•	•	○	•	○	•	•	•	•	Moderate

**Poorolajal 2014**	●	◎	●	◎	●	●	◎	●	●	●	●	●	●	●	●	●	High
**Qiu 2016**	●	●	●	○	○	○	●	●	●	●	●	○	●	●	●	●	Moderate
**Qu 2014**	●	◎	●	◎	●	●	○	●	●	●	●	●	●	●	●	●	Low
**Reid 2011**	●	○	○	○	○	○	○	●	●	○	●	○	○	○	○	●	Critically low
**Salar 2019**	●	◎	●	◎	○	●	◎	●	●	○	●	○	○	○	●	○	Low
**Santucci 2019**	●	●	●	●	○	○	○	●	○	○	●	○	○	●	●	○	Critically low
**Schmid 2014**	●	◎	●	◎	○	●	◎	●	◎	○	●	○	●	●	●	●	Moderate
**Shafei 2019**	●	◎	●	◎	●	●	◎	○	○	●	●	○	●	●	●	●	Low
**Shi 2015**	●	◎	○	◎	○	●	◎	●	◎	●	●	○	●	●	●	●	Moderate
**Siristatidis 2013**	●	●	●	◎	●	●	○	●	◎	●	●	●	●	●	○	●	Critically low
**Song 2017**	●	◎	●	◎	○	●	○	●	◎	○	●	○	○	●	○	●	Critically low
**Song 2018**	●	●	○	◎	●	●	○	◎	○	○	●	○	○	●	●	●	Critically low
**Wallin 2011**	●	○	●	◎	○	●	○	●	○	○	●	○	○	●	●	○	Critically low
**Wang 2018**	●	◎	●	◎	●	●	◎	●	◎	○	●	○	●	●	●	●	Moderate
**Wen 2019**	●	◎	●	◎	○	●	◎	●	◎	●	●	●	●	●	●	●	Moderate
**Whiteman 2000***	●	○	○	○	○	○	●	●	◎	○	●	●	●	●	○	○	Low
**Wu 2019**	●	◎	●	◎	●	●	◎	●	◎	●	●	○	●	●	●	●	Moderate
**Xu 2018**	●	◎	●	◎	●	●	◎	●	◎	●	●	○	●	●	●	●	Moderate
**Yan 2015**	●	◎	●	◎	●	●	○	●	◎	○	●	○	●	●	●	●	Low
**Yang 2019**	●	●	○	●	○	○	○	◎	●	○	●	●	●	○	●	●	Moderate
**Yin 2011**	●	◎	●	◎	●	●	○	●	○	●	●	○	●	●	●	●	Critically low
**Zeng 2015**	●	◎	●	◎	●	●	◎	●	●	○	●	●	●	●	●	●	Moderate
**Zeng 2016**	●	◎	●	◎	●	●	◎	●	●	●	●	●	●	●	●	●	Moderate
**Zhan 2017**	●	◎	●	◎	○	○	◎	●	●	●	●	○	●	●	●	●	Moderate
**Zhang 2016**	●	◎	●	◎	●	●	◎	●	●	●	●	○	●	●	●	●	Moderate
**Zhang 2017**	●	◎	●	◎	●	●	◎	●	●	●	●	○	●	●	●	●	Moderate
**Zhang 2018 a**	●	◎	●	◎	●	●	◎	●	●	●	●	○	●	●	●	●	Moderate
**Zhang 2018 b**	●	◎	●	◎	●	●	○	●	●	○	●	○	●	●	●	●	Low
**Zheng 2018**	●	●	●	◎	○	●	○	●	●	●	●	○	●	●	●	○	Low
**Zhong 2014**	●	◎	●	◎	○	●	○	●	●	○	●	○	●	○	●	●	Low
**Zhou 2014**	●	◎	●	◎	●	●	○	●	●	○	●	○	●	○	●	○	Critically low
**Zhou 2017**	●	◎	●	◎	○	●	○	●	●	●	●	○	●	●	●	●	Low
**Zhou 2008**	●	◎	●	○	○	●	○	●	●	●	●	○	●	●	●	○	Critically low

Key: ● Yes
◎ Partial yes
○ No
* Pooled analysis
NA Not Applicable
Critical flaw

Supplementary Table S7: Details of evidence grading for meta-analyses of risk factors for ovarian cancer incidence or mortality[κ] – all statistically significant study types included*

Exposure	Exposure contrast	N[*]	Sample size Cases/Cohort	Largest study[#]	Random effects summary RR (95% CI) [¥]	Random P-value[||]	95% Prediction interval	Egger's P	I2 (%)	Excess significance		Evidence Grading**
										O/E [α]	P-value	
**Strong evidence**												
**Anthropometric indices**												
Height	per 10cm	16	18663/13600000	1.14 (1.10-1.18)	1.16 (1.11-1.20)	2.2E-13	1.06-1.26	0.18	27	9/6.22	0.15	1: Strong
BMI	BMI ≥30kg/m[2] vs normal	13	6947/ 20,560,388ι	1.27 (1.19-1.36)	1.37 (1.17-1.38)	2.6E-8	1.09-1.47	0.88	12	3/5.30	NP	1: Strong
**Use of medical or hormonal therapy**												
HRT- Prospective	Ever vs never	17	12110/950663	1.15 (1.06-1.26)	1.20 (1.13-1.28)	2.1E-09	1.13-1.28	0.71	0	4/4.00	NP	1: Strong
HRT - Prospective	Ever vs never (info duration of use and times since last use)	14	11866/949657	1.16 (1.05-1.28)	1.24 (1.16-1.32)	6.0E-10	1.15-1.33	0.97	0	2/4.87	NP	1: Strong
HRT- Prospective	Cur/rec vs never	12	11664/948390	1.28 (1.14-1.44)	1.37 (1.27-1.48)	1.3E-15	1.26-1.50	0.68	0	3/7.20	NP	1: Strong
Repro factors: OCP	Ever vs never	45	19610/90211	0.74 (0.67-0.82)	0.74 (0.70-0.78)	9.0E-30	0.70-0.78	0.16	0	11/23	NP	1: Strong
**Highly suggestive evidence**												
**Reproductive factors**												
Breastfeeding	Ever vs Never	55	17139/415985	0.53 (0.46-0.62)	0.69 (0.34-0.76)	9.6E-16	0.43-1.13	0.71	76	24/3.85	<1e-100	2: Highly suggestive
**Use of medical or hormonal therapy**												
HRT	Ever vs never; ET only	11	7512/2302683	1.31 (1.11-1.54)	1.44 (1.25-1.66)	7.1E-07	0.99-2.09	0.71	48	6/7.01	NP	2: Highly suggestive
Metformin	Ever vs never	3	3288/513702	0.16 (0.14-0.17)	0.18 (0.12-0.25)	2.5E-23	0.01-4.31	0.38	NA	2/2.42	NP	2: Highly suggestive
**Medical history**												
Endometriosis	Any vs none	25	15921/858822	1.46 (1.31-1.63)	1.99 (1.67-2.32)	1.3E-17	1.05-3.75	0.00	76	22/17.50	0.08	2: Highly suggestive
**Carcinogens/ environmental irritants**												
Genital talc powder use	Ever vs never	27	15154/367198	1.32 (1.14-1.50)	1.26 (1.17-1.35)	1.3E-10	1.00-1.59	0.70	39	13/17.20	NP	2: Highly suggestive
**Suggestive evidence**												
**Reproductive factors**												
Tubal ligation	Ever vs never	25	31678/373632	0.81 (0.73-0.91)	0.70 (0.60-0.81)	1.3E-06	0.35-1.38	0.78	86	16/14.30	0.5	3: Suggestive
**Use of medical or hormonal therapy**												
HRT	Current vs ever	5	3958/1342899	1.20 (1.09-1.32)	1.28 (1.15-1.42)	6.5E-06	1.01-1.62	0.08	14	3/2.91	NP	3: Suggestive
**Anthropometric indices**												
BMI	per 5kg/m2 increase	25	17734/16300000	0.97 (0.93 1.01)	1.07 (1.04-1.11)	<0.01	0.96-1.21	0.07	48	6/1.80	0.00	3: Suggestive
BMI	iya per 5kg/m2 increase	6	9452/11100000	1.16 (1.04 1.29)	1.12 (1.05 -1.19)	<0.01	1.03-1.23	0.60	0	1/2.48	NP	3: Suggestive
**Carcinogen/ environmental irritants**												
Asbestos[κ]	Any vs none	16	5165/906145	1.30 (0.90-1.80)	1.86 (1.46-2.36)	5.0E-07	1.05 -3.29	0.64	28	4/1.87	0.1	3: Suggestive
Perineal talc use	Long term (>10yrs) vs none	12	2125/134414	1.29 (1.05-1.59)	1.25 (1.10-1.43)	<0.01	0.88-1.79	0.30	45	4/1.05	0.53	3: Suggestive

**Medical history**												
Diabetes Mellitus	DM vs no DM	17	5036/2868215	1.23 (1.15-1.32)	1.32 (1.14 -1.52)	<0.01	0.81-2.15	0.30	80	6/6.71	NP	3: Suggestive
**Dietary factors**												
Polyphenol; Genistein	HvL	4	1239/99776	0.42 (0.30-0.60)	0.56 (0.42-0.76)	<0.01	0.18-1.72	0.26	46	2/4.00	NP	3: Suggestive
Polyphenol; Soyfoods	HvL	6	2617/180668	0.54 (0.43-0.69)	0.51 (0.39-0.68)	2.9E-06	0.22-1.23	0.84	70	4/5.82	NP	3: Suggestive
Polyphenol; Tofu	HvL	4	1111/162722	0.57 (0.40-0.80)	0.59 (0.47-0.75)	<0.01	0.35-1.00	0.87	0	2/3.76	NP	3: Suggestive
Dietary fibre intake	Per 10g/day increase	9	3492/137025	0.91 (0.81-1.01)	0.91 (0.81-1.01)	5.0E-05	0.79-0.97	0.58	7	3/1.31	0.11	3: Suggestive
Dietary inflammatory index	HvL	4	3104/7982	1.31 (1.06-1.62)	1.31 (1.06-1.62)	8.6E-06	1.01-1.98	0.39	0	3/3.07	NP	3: Suggestive
**Biochemical markers**												
CRP	>10mg/L vs <10mg/L	4	1413/31442	1.67 (1.03-2.70)	2.14 (1.47-3.11)	<0.01	0.77-5.93	0.15	13	2/3.85	NP	3: Suggestive
**Weak evidence**												
**Reproductive factors**												
Breastfeeding	Per 5mo increase in duration	25	1180/447386	0.98 (0.92-1.05)	0.94 (0.89-1.00)	0.03	0.80-1.49	0.59	22	1/0.17	0.04	4: Weak
Breastfeeding	>12mo vs never	6	2833/11659	1.08 (0.87-1.34)	0.72 (0.53-0.97)	0.03	0.29-1.82	0.30	71	2/0.74	0.12	4: Weak
PID	Ever vs never	14	8285/2929284	1.05 (0.92-1.20)	1.32 (1.05-1.66)	1.6E-02	0.71-2.47	0.35	65	2/1.01	0.28	4: Weak
Age at menarche	Oldest vs youngest	24	8872/1022451	0.77 (0.62-0.95)	0.85 (0.75-0.97)	0.01	0.57-1.28	0.87	42	4/1.31	<0.01	4: Weak
IVF	Ever vs never (ref group gen population excl OC diagnosis <1yr post treatment)	6	31606/1438001	1.30 (0.90-1.88)	1.47 (1.06-2.03)	2.0E-02	0.73-2.96	0.64	23	1/1.55	NP	4: Weak
IVF	Ever vs never ref group IVF pop; total follow up	6	31606/1438002	1.35 (0.93-1.96)	1.66 (1.08-2.55)	2.2E-02	0.52-5.28	0.91	52	2/1.64	0.74	4: Weak
Nulliparity	Yes vs no	3	285/835	1.40 (1.10-1.70)	1.42 (1.18-1.72)	<0.01	0.42-4.84	0.87	0	1/0.89	0.89	4: Weak
Age at last birth	Highest vs lowest	13	19959/2470521	0.93 (0.85-1.01)	0.77 (0.65-0.91)	2.2E-03	0.45-1.32	0.58	74	5/2.37	0.06	4: Weak
**Use of medical or hormonal therapy**												
OCP	Ever vs never	22	60/80670	0.60 (0.30-1.40)	0.43 (0.25-0.75)	3.0E-03	0.01-15.16	0.41	13	1/0.73	0.71	4: Weak
Aspirin	Ever vs never	22	14581/511293	0.94 (0.85-1.04)	0.89 (0.83-0.96)	<0.01	0.75-1.06	0.01	23	3/2.74	0.86	4: Weak
Aspirin	Ever vs never CC	14	12593/101479	0.94 (0.85-1.04)	0.85 (0.77-0.94)	<0.01	0.68-1.10	0.01	38	3/2.07	0.48	4: Weak
NSAIDS, Non aspirin	Ever vs never	16	1782/505136	0.90 (0.75-1.08)	0.90 (0.81-1.00)	4.4E-02	0.78-1.04	0.55	0	0/1.02	NP	4: Weak
Statin use	Any vs none long-term	3	231/1829	0.54 (0.27-1.09)	0.48 (0.28-0.80)	<0.01	0.02-13.95	0.31	0	1/2.04	NP	4: Weak
Statin use	Any vs none	5	648/84234	0.83 (0.66-1.05)	0.79 (0.64-0.98)	0.03	0.57-1.11	0.23	0	0/1.88	NP	4: Weak
Antidepressants	Ever vs never	19	8200/571701	0.99 (0.83-1.18)	0.70 (0.57-0.87)	<0.01	0.28-1.75	0.28	83	7/0.97	<1e-100	4: Weak
HRT	Invasive; ever vs never	10	4392/255821	1.15 (0.94-1.42)	1.16 (1.01-1.32)	0.04	0.84-1.59	0.17	32	2/2.82	NP	4: Weak
HRT	Invasive and borderline; ever vs never	12	5421/260387	1.15 (0.94-1.42)	1.13 (1.01-1.27)	0.03	0.90-1.43	0.14	21	2/3.36	NP	4: Weak
HRT	Ever vs never (cont E+P)	4	3337/1265735	1.13 (0.96-1.34)	1.22 (1.06-1.40)	<0.01	0.90-1.65	0.07	0	1/1.63	NP	4: Weak
HRT	Ever vs never (seq E+P)	4	3337/1265735	1.14 (0.98-1.32)	1.35 (1.06-1.72)	1.5E-02	0.54-3.35	0.18	51	2/1.75	0.80	4: Weak
HRT	Ever vs never ET+PT	11	7512/2302683	1.50 (1.34-1.68)	1.23 (1.08-1.14)	2.3E-03	0.87-1.75	0.34	53	3/8.56	NP	4: Weak
HRT	Ever vs never ET +E/PT	2	543/141880	1.50 (0.92-2.44)	1.55 (1.05-2.30)	2.7E-02	NA	NA	0	0/1.89	NP	4: Weak

**Anthropometric indices**												
BMI	PrMP BMI ≥30kg/m^2 vs normal	3	71/350211	1.56 (1.14-2.16)	1.57 (1.20-2.06)	<0.01	0.27-9.02	0.61	0	1/0.66	0.64	4: Weak
BMI	PoMP; BMI ≥30kg/m^2 vs normal	5	350/546195	1.02 (0.82-1.26)	1.23 (1.03-1.47)	2.6E-01	0.72-2.09	0.52	46	1/0.25	0.13	4: Weak
BMI	BMI Highest vs lowest	9	1801/335870	1.20 (0.90-1.50)	1.20 (1.00-1.44)	0.05	0.74-1.95	0.37	47	3/1.63	0.24	4: Weak
Weight	Per 5kg weight	4	1006/297350	1.02 (1.00-1.05)	1.03 (1.01-1.05)	<0.01	0.98-1.08	0.42	7	1/0.21	0.08	4: Weak
Weight gain	per 5kg increase PoMP, HRT	2	217/23984	1.16 (1.03-1.31)	1.13 (1.03-1.24)	<0.01	NA	NA	NA	1/0.27	0.13	4: Weak
**Carcinogen/ environmental irritant**												
Asbestos; Occupational[κ]	Total exposed vs nonexposed	20	126/21973	1.12 (0.66-1.80)	1.77 (1.37-2.27)	9.7E-06	0.85-3.66	0.72	35	6/1.06	<0.01	4: Weak
Asbestos; Occupational[κ]	High exposed vs nonexposed	6	20/6149	1.10 (0.37-2.21)	2.78 (1.36-5.66)	<0.4	0.42-18.44	0.78	45	2/0.31	<0.01	4: Weak
**Pre existing medical conditions/ interventions**												
SLE	Observed vs expected	5	44/40855	0.82 (0.54-1.20)	0.73 (0.53-1.00)	4.9E-02	0.36-1.46	0.97	0	0/0.26	NP	4: Weak
**Dietary factors**												
Calcium	HvL	13	1726/351192	0.86 (0.68-1.10)	0.86 (0.74-1.00)	4.0E-02	0.67-1.09	0.62	0	0/1.66	NP	4: Weak
Calcium; dairy	HvL	4	1876/169084	0.86 (0.68-1.10)	0.80 (0.66-0.98)	0.03	0.41-1.59	0.57	34	1/1.50	NP	4: Weak
Cruciferous veg	HvL	11	4306/378489	0.87 (0.74-1.03)	0.89 (0.80-1.00)	0.04	0.71-1.13	0.49	25	1/3.08	NP	4: Weak
Dietary pattern; healthy	HvL	11	3055/395285	0.95 (0.83-1.06)	0.85 (0.74-0.98)	0.02	0.57-1.28	0.27	57	2/0.93	0.24	4: Weak
Dietary pattern; western	HvL	11	3168/328501	1.02 (0.85-1.24)	1.19 (1.00-1.41)	0.04	0.68-2.10	0.87	73	2/0.59	0.06	4: Weak
Dietary protein intake	HvL	10	2354/74464	0.92 (0.82-1.02)	0.92 (0.85-1.00)	3.9E-02	0.83-1.01	0.46	0	0/0.97	NP	4: Weak
Decaffeinated coffee	Ever vs never CC	5	2663/7907	0.71 (0.51-0.99)	0.72 (0.58-0.90)	4.3E-03	0.50-1.04	0.55	0	2/4.03	NP	4: Weak
Dairy Total dairy products	HvL	7	427/90001	1.61 (1.07-2.42)	1.66 (1.19-2.31)	<0.01	NA	NA	NA	1/1.87	NP	4: Weak
Dairy Skim/low fat	HvL	11	728/170327	1.32 (0.97-1.82)	1.35 (1.09-1.68)	<0.01	0.35-5.43	0.21	0	0/1.93	NP	4: Weak
Dairy; whole milk	HvL	9	3590/150503	1.10 (0.75-1.60)	1.25 (1.00-1.56)	0.04	0.67-2.33	0.96	52	2/1.20	0.43	4: Weak
Dairy Lactose	HvL	12	728/170327	1.48 (1.05-2.09)	1.47 (1.17-1.84)	<0.01	0.34-6.29	0.42	0	1/2.64	NP	4: Weak
Egg consumption	HvL	12	3728/632197	1.14 (0.67-1.20)	0.19 (1.00-1.41)	0.04	0.75-1.88	0.56	42	2/2.51	NP	4: Weak
Fat; total fat	HvL	17	7801/760734	1.07 (0.89-1.29)	1.19 (1.04-1.37)	0.01	0.75-1.89	0.57	60	4/1.82	0.09	4: Weak
Fat; trans	HvL	4	2955/152165	1.30 (1.08-1.57)	1.25 (1.08-1.44)	<0.01	0.91-1.71	0.60	0	2/3.29	NP	4: Weak
Meat; Red	HvL	10	5589/358333	1.30 (1.10-1.60)	1.17 (1.02-1.35)	0.03	0.80-1.71	0.95	46	3/7.13	NP	4: Weak
Meat; processed	HvL	7	1018/696100	1.23 (0.92-1.63)	1.26 (1.02-1.56)	3.5E-02	0.31-5.07	0.37	0	0/1.56	NP	4: Weak
Meat; red and processed[κ]	Per 100g/week increment	21	6536/2140286	1.02 (0.98-1.06)	1.01 (1.00-1.04)	3.4E-02	1.00-1.04	0.11	0	0/1.14	NP	4: Weak
Non starchy vegetables	Per 100g/day	6	2053/641079	1.00 (0.93-1.07)	0.94 (0.89-1.00)	4.0E-02	0.82-1.08	0.21	28	1/0.30	0.19	4: Weak
Non herbal tea	HvL	19	734/164882	0.63 (0.40-0.99)	0.69 (0.52-0.93)	1.4E-02	0.11-4.57	0.03	0	1/2.74	NP	4: Weak
Total phytoestrogen intake	HvL	10	4392/298165	1.05 (0.85-1.31)	0.70 (0.56-0.87)	<0.01	0.34-1.43	0.04	72	6/0.78	<1E-100	4: Weak
Isoflavones	HvL	6	2703/164267	0.51 (0.37-0.69)	0.63 (0.46-0.86)	<0.01	0.23-1.70	0.30	68	4/5.99	NP	4: Weak
Daidzein	HvL	4	1239/99776	0.41 (0.29-0.59)	0.60 (0.42-0.86)	<0.01	0.14-2.50	0.33	62	2/ 4.00	NP	4: Weak
Glycitein	HvL	3	959/2501	0.38 (0.37-9.55)	0.55 (0.35-0.86)	<0.01	0.00-86.89	0.22	66	2/ 3.00	NP	4: Weak

Kaempferol	HvL	3	347/66940	0.60 (0.42-0.87)	0.60 (0.42-0.86)	6.0E-03	NA	NA	NA	1/0.99	0.98	4: Weak
Flavones	HvL	4	347/66941	0.69 (0.52-0.92)	0.69 (0.52-0.92)	1.1E-02	NA	NA	NA	1/0.97	0.86	4: Weak
Soybean	HvL	2	754/1906	0.62 (0.45-0.85)	0.48 (0.28-0.84)	<0.01	NA	NA	NA	2/1.93	0.79	4: Weak
Polyphenol; Non asian	HvL	7	3561/232718	1.05 (0.85-1.31)	0.74 (0.58-0.96)	0.02	0.33-1.69	0.13	76	4/0.59	<0.01	4: Weak
Polyphenol; Asian	HvL	3	831/65447	0.45 (0.29-0.59)	0.48 (0.37-0.63)	2.0E-07	0.08-2.87	0.10	0	2/2.97	NP	4: Weak
Tea	HvL	18	8683/708263	1.20 (1.00-1.40)	0.89 (0.80-1.00)	0.04	0.64-1.25	0.01	43	5/8.02	NP	4: Weak
Tea; black	HvL	15	1299/203998	0.63 (0.40-0.99)	0.73 (0.56-0.93)	1.2E-02	0.42-1.24	0.44	15	2/4.71	NP	4: Weak
Tea; green	HvL	4	2580/5991	0.84 (0.64-1.12)	0.63 (0.42-0.93)	0.02	0.11-3.46	0.69	71	2/1.67	0.74	4: Weak
**Biochemical markers**												
CRP	Middle vs low	7	159/27746	1.52 (1.02-2.28)	1.52 (1.01-2.27)	0.04	NA		NA	1/0.83	0.65	4: Weak
CRP	HvL	7	1898/32580	1.35 (1.06-1.71)	1.35 (1.06-1.71)	0.02	0.67-2.70	0.09	61	4/3.68	0.81	4: Weak
**Physical activity**												
Recreational	Moderate vs low	20	11765/798786	1.10 (0.95-1.28)	0.91 (0.85-0.99)	0.02	0.71-1.18	0.59	45	4/4.07	1.00	4: Weak

Abbreviations: BMI, body mass index; BMI iya, body mass index in young adulthood; BMI PoMP, body mass index postmenopausal; BMI PrMP, body mass index premenopausal; CC, case control; CRP, c-reactive protein; E+P, estrogen and progesterone; E+E/P, estrogen and estrogen/progesterone; HRT, hormone replacement therapy; HvL, highest versus lowest; IVF, in-vitro fertilisation; NA, not available; np; not pertinent, because the estimated is larger than the observed, and there is no evidence of excess statistical significance based on the assumption made for the plausible effect size; NSAID, non- steroidal anti -inflammatory drugs; OC, ovarian cancer; OCP, oral contraceptive pill; PID, pelvic inflammatory disease; RR, relative risk; SLE, systemic lupus erythematous; WG, weight gain

Key:
*only meta-analyses meeting at least weak grade of evidence listed
[*] Number of studies
[#] Relative risk and 95% confidence interval of largest study (smallest standard error) in each meta-analysis
[¥] Random effects refer to summary risk ratio (95% confidence interval) using the random-effects model
[||] P value of summary random effects estimate
[∞] P-value from the Egger's regression asymmetry test
[§] Expected number of statistically significant studies using the point estimate of the largest study (smallest standard error) as the plausible effect size
[α] Observed/Expected number of statistically significant studies
φP value of the excess statistical significance test
All statistical tests were two-sided
¶Small study effect is based on the P-value from the Egger's regression asymmetry test (P>0.1) where the random effects summary estimate was larger compared to the point estimate of the largest study in a meta-analysis
†Based on the p-value (P>0.1) of the excess significance test using the largest study (smallest standard error) in a meta-analysis as the plausible effect size
ι Person years
κRisk of dying from ovarian cancer

**Summary of evidence grading criteria:

Weak	P<0.05[||]
Suggestive	$P<10^{-3}$[||]; >1,000 cases

Highly suggestive	$P<10^{-6}$[‖]; >1,000 cases; $P<0.05$ of the largest study in a meta-analysis
Strong	$P<10^{-6}$[‖]; >1,000 cases; $P<0.05$ of the largest study in a meta-analysis; $I^2<50\%$; no small study effect[¶]; prediction interval excludes the null value; no excess significance bias[†]

Supplementary Table S8: Sensitivity analysis using credibility ceilings when the association is non-significant of the 40 studies investigating the risk factors associated with incidence or mortality[§] of ovarian cancer - only cohort studies included.

Author, year	Exposure	Exposure contrast	N$^{\alpha}$ studies	Credibility ceiling (%) when association non-significant
**Reproductive factors**				
Luan 2013	Breastfeeding	Per 5month increase in duration	25	3
Siristatidis 2013	IVF	Ever vs never (ref group general population excluding OC diagnosis <1year post treatment)	6	3
Siristatidis 2013	IVF	Ever vs never ref group IVF pop; total follow up	6	3
Zhou 2017	PID	Ever vs never	14	3
**Use of medical or hormonal therapy**				
Baandrup 2013	NSAIDS Non aspirin	Ever vs never	16	5
Collaborative Group 2015	HRT- Prospective	Current/recent vs never	12	22
Collaborative Group 2015	HRT- Prospective	Ever vs never	17	19
Collaborative Group 2015	HRT- Prospective	Ever vs never (information on duration of use and time since last use)	14	16
Coll Gr 2008	OCP	Ever vs never	45	22
Hankinson 1992	OCP	Ever vs never	22	12
Shi 2015	HRT	Ever vs never ET only	11	15
Shi 2015	HRT	Ever vs never (continuous E+P)	4	13
Shi 2015	HRT	Ever vs never (sequential E+P)	4	10
Shi 2015	HRT	Ever vs never ET+PT	11	14
Shi 2015	HRT	Ever vs never ET + E/PT	2	9
Zhou 2008	HRT	Current vs ever	5	18
Wen 2019	Metformin	Ever vs never	3	10
**Anthropometric indices**				
Aune 2015	Anthropometric measure	Height per 10cm	16	25
Aune 2015	Anthropometric measure	BMI per 5kg/m^{2}increase	25	10
Aune 2015	Anthropometric measure	BMI iya per 5kg/m^2 increase	6	13
Aune 2015	Anthropometric measure	Per 5kg weight	4	12
Keum 2015	Anthropometric measure	WG per 5kg increase PoMP, HRT	2	6
Poorolajal 2014	Anthropometric measure	BMI ≥30kg/m^2 vs normal	13	15
Poorolajal 2014	Anthropometric measure	BMI PrMP; ≥30kg/m^2 vs normal	3	8
Poorolajal 2014	Anthropometric measure	BMI PoMP; ≥30kg/m^2 vs normal	5	3
**Carcinogens/ environmental irritants**				

Reid 2011	Asbestos[§]	Any vs none	16	22
Camargo 2011	Asbestos; Occupational[§]	Total expected vs unexpected	20	14
Camargo 2011	Asbestos; Occupational[§]	High expected vs unexpected	6	7
**Medical history**				
Bernatsky 2011	SLE	Observed vs expected	5	5
Zhang 2017	Diabetes Mellitus	DM vs no DM	17	9
Li 2019	Endometriosis	Any vs none	25	22
**Dietary factors**				
Larsson 2006	Dairy Total dairy products	Highest vs lowest	7	9
Larsson 2006	Dairy Skim/low fat	Highest vs lowest	11	12
Larsson 2006	Dairy Lactose	Highest vs lowest	12	13
Song 2017	Calcium	Highest vs lowest	13	6
Wallin 2011	Meat; red and processed[§]	Per 100g/week increment	21	18
Kolahdooz 2010	Meat; processed	Highest vs lowest	7	12
WCRF CUP 2013	Non starchy vegetables	Per 100g/day	6	2
Zhang 2018	Non herbal tea	Highest vs lowest	19	6
Butler 2011	Tea; black	Highest vs lowest	15	6

[α] Number
[§] Risk of dying from ovarian cancer

Key: BMI, body mass index; CC, case-control; DM, diabetes mellitus; ET, estrogen therapy; E+P, estrogen and progesterone; E/PT, combined estrogen/progesterone therapy; g, gram; HRT, hormone replacement therapy; IVF, in vitro fertilisation; iya, in young adulthood; kg, kilogram; m, metre; m^2, metre squared; NSAIDS, non-steroidal anti-inflammatory drugs; OCP, oral contraceptive pill; PID, pelvic inflammatory disease; PoMp, postmenopausal; PrMp, premenopausal; PT, progesterone therapy; ref, reference; SLE, systemic lupus erythematous; WG, weight gain

Supplementary Table S9: Evaluation of heterogeneity, small study effects and excess significance bias in the 88 statistically significant meta-analyses investigating the risk factors associated with ovarian cancer incidence or mortality§ – all study types included.

Author, year	Exposure	Exposure contrast	Egger's P[a]	I2 (95% CI) P[b]	Studies	Observed	Expected[c], P-value[d]					
							Fixed effects		Random effects		Largest study	
**Anthropometric indices**												
Pooralajal 2014	BMI	≥30kg/m^2 vs normal	0.88	12 (0-54) 0.33	13	3	5.20	NP	5.20	NP	5.30	NP
Pooralajal 2014	BMI	≥30kg/m^2 vs normal, PrMP	0.61	0 (0 -73) 0.86	3	1	0.68	0.66	0.68	0.66	0.66	0.64
Poorolajal 2014	BMI	≥30kg/m^2 vs normal, PoMP	0.52	46 (0-79) 0.12	5	1	0.71	0.71	0.79	0.80	0.25	0.13
Aune 2015	BMI	per 5kg/m2 increase	0.07	42 (0-69) 0.65	25	5	2.93	0.55	3.86	0.23	1.80	0.00
Aune 2015	BMI	iya per 5kg/m2 increase	0.60	48 (6-67) 0.00	6	6	2.00	NP	2.00	NP	2.48	NP
Liu 2015	BMI	25-29.9kg/m^2 vs normal	0.31	11 (0-46) 0.30	25	3	3.00	1.00	3.44	NP	2.11	0.52
Olsen 2007	BMI	HvL	0.37	47 (0-74) 0.06	9	3	1.81	0.32	1.65	0.24	1.63	0.24
Aune 2015	Height	Per 10cm	0.18	7 (0-70) 0.36	16	1	6.51	0.21	6.86	0.28	6.22	0.15
Keum 2015	Weight gain	Per 5kg; ever HRT (PoMP)	NA	27 (0-59) 0.15	2	9	0.21	0.69	0.21	0.69	0.27	0.13
**Dietary factors**												
Wang 2018	Alcohol consumption	HvL, heavy vs non/low drinking	NA	NA	6	1	0.99	0.94	0.99	0.94	0.99	0.94
Song 2017	Calcium	HvL	0.62	0 (0-64) 0.61	13	0	1.73	NP	1.73	NP	1.66	NP
Song 2017	Calcium	HvL, dairy	0.57	34 (0-78) 0.21	4	1	2.38	NP	2.53	NP	1.50	NP
Han 2014	Cruciferous veg	HvL	0.49	25 (0-63) 0.20	11	1	2.11	NP	2.19	NP	3.08	NP
Shafiei 2019	Decaffeinated coffee- cc	Ever vs never	0.55	0 (0-64) 0.76	5	2	3.91	NP	3.91	NP	4.03	NP
Pang 2018	Dietary protein intake	HvL	0.46	0.(0-53) 0.80	10	0	1.01	NP	1.01	NP	0.97	NP
Zheng 2018	Dietary fibre intake	Per 10g/day increase	0.58	7 (0-58) 0.37	9	3	2.11	0.48	2.11	0.48	1.31	0.11
Wang 2018	Dietary pattern; healthy	Highest vs lowest	0.27	57 (0-76) 0.01	11	2	1.94	0.96	2.90	NP	0.93	0.25
Wang 2018	Dietary pattern; western style	Highest vs lowest	0.87	73 (45-84) 0.00	11	2	3.18	0.43	3.39	0.37	0.59	0.06
Liu 2019	Dietary inflammatory index	Highest vs lowest	0.39	0 (0-68) 0.66	4	3	3.49	NP	3.49	NP	3.07	NP
Larsson 2006	Dairy	HvL, total dairy products	NA	NA	7	1	1.91	NP	1.91	NP	1.87	NP
Larsson 2006	Dairy	HvL, skim/low fat	0.21	0 (0-73) 0.64	11	0	2.14	NP	2.14	NP	1.93	NP
Larsson 2006	Dairy	HvL, lactose	0.42	0 (0-73) 0.92	12	1	2.61	NP	2.61	NP	2.64	NP
Larsson 2006	Dairy	HvL, whole	0.96	52 (0-76) 0.04	9	2	4.34	NP	4.45	NP	1.20	0.43
Zeng 2015	Egg consumption	Highest vs lowest	0.56	42 (0-69) 0.06	12	2	4.26	NP	3.82	NP	2.51	NP
Qiu 2016	Fat; total fat	Highest vs lowest	0.57	60 (22-75) 0.00	17	4	5.82	NP	6.65	NP	1.82	0.09
Qiu 2016	Fat; trans	Highest vs lowest	0.60	0 (0-68) 0.39	4	2	2.84	NP	2.83	NP	3.29	NP
Kolahdooz 2010	Meat	HvL, red	0.95	46 (0-73) 0.06	10	3	4.31	NP	3.96	NP	7.13	NP
Kolahdooz 2010	Meat	HvL, processed	0.37	0 (0-73) 0.93	7	0	1.79	NP	1.79	NP	1.56	NP
Wallin 2011	Meat	Per 100g/wk increment; red and processed§	0.11	0 (0-41) 1.00	21	0	1.13	NP	1.13	NP	1.14	NP
WCRF CUP 2013	Non starchy vegetables	Per 100g/day	0.21	28 (0-71) 0.23	6	1	0.49	0.44	0.58	0.57	0.30	0.19
Zhang 2018	Non herbal tea	HvL	0.03	0 (0-73) 0.53	19	1	2.44	NP	2.44	NP	2.74	NP
Qu 2014	Polyphenol; Total phytoestrogen intake	HvL	0.04	72 (36-84) 0.00	10	6	5.71	0.85	7.87	NP	0.78	<1E-100
Qu 2014	Polyphenol; Isoflavones	HvL	0.30	68 (0-85) 0.01	6	4	5.81	NP	5.63	NP	5.99	NP
Qu 2014	Polyphenol; Daidzein	HvL	0.33	62 (0-85) 0.05	4	2	3.92	NP	3.86	NP	4.00	NP
Qu 2014	Polyphenol; Flavones	HvL	NA	NA	4	1	0.97	0.86	0.97	0.86	0.97	0.86

Qu 2014	Polyphenol; Kaempferol	HvL	NA	NA	2	1	0.99	0.98	0.99	0.98	0.99	0.98
Qu 2014	Polyphenol; Genistein	HvL	0.26	46 (0-81) 0.14	3	2	3.97	NP	3.94	NP	4.00	NP
Qu 2014	Polyphenol; Glycitein	HvL	0.22	66 (0-88) 0.05	3	2	2.99	NP	2.97	NP	3.00	NP
Qu 2014	Polyphenol; Soyfoods	HvL	0.84	70 (0-85) 0.01	6	4	5.86	NP	5.88	NP	5.82	NP
Qu 2014	Polyphenol; Tofu	HvL	0.87	0 (0-68) 0.67	4	2	3.68	NP	3.68	NP	3.76	NP
Qu 2014	Polyphenol; Soybean	HvL	NA	NA	2	2	2.00	0.96	2.00	0.99	1.93	0.79
Qu 2014	Polyphenol; Non asian	HvL	0.13	76 (39-87) 0.00	7	4	3.69	0.81	5.03	NP	0.59	0.00
Qu 2014	Polyphenol; Asian	HvL	0.10	0(0-73) 0.79	3	2	2.94	NP	2.94	NP	2.97	NP
Zhan 2017	Tea	HvL	0.01	43 (0-66) 0.03	18	5	1.91	0.02	3.97	0.56	8.02	NP
Butler 2011	Tea; green	HvL	0.69	71 (0-88) 0.01	4	2	3.44	NP	3.60	NP	1.67	0.74
Butler 2011	Tea; black	HvL	0.44	15 (0-69) 0.32	15	2	3.88	NP	3.84	NP	4.71	NP
**Medical history**												
Zhang 2017	Diabetes Mellitus	DM vs no DM	0.30	80 (67-86) 0.00	17	6	6.33	NP	8.29	NP	6.71	NP
Li 2019	Endometriosis	Any vs none	0	76 (63-82) 0.00	25	21	18.90	0.34	22.40	NP	16.90	0.08
Bernatsky 2011	SLE	Observed vs expected	0.97	0 (0-68) 0.53	**5**	0	0.36	NP	0.36	NP	0.26	NP
**Use of medical/ hormonal therapy**												
Zhang 2016	Aspirin	Ever vs never	0.01	23 (0-54) 0.17	22	3	4.39	NP	5.92	NP	2.74	0.86
Zhang 2016	Aspirin	Ever vs never CC	0.01	38 (0-66) 0.07	14	3	4.07	NP	6.84	NP	2.07	0.48
Huo 2018	Antidepressants	Ever vs never	0.28	83 (75-88) 0.00	19	7	12.40	NP	15.2	NP	0.97	<1E-100
Collab Gr 2015	HRT- Prospective	Current/recent vs never	0.68	0 (0-50) 0.77	12	3	8.72	NP	8.72	NP	7.20	NP
Collab Gr 2015	HRT- Prospective	Ever vs never	0.71	0 (0-45) 0.81	17	4	6.27	NP	6.27	NP	4.90	NP
Collab Gr 2015	HRT – Prospective	Ever vs never (info duration of use and times since last use)	0.97	0(0-48) 0.45	14	2	6.71	NP	6.72	NP	4.87	NP
Garg 1998	HRT	Ever vs never; Invasive	0.17	32 (0-67) 0.15	10	2	2.68	NP	3.00	NP	2.82	NP
Garg 1998	HRT	Ever vs never; Invasive and borderline	0.14	21 (0-60) 0.24	12	2	2.63	NP	2.81	NP	3.36	NP
Shi 2015	HRT	Ever vs never (cont E+P)	0.07	0 (0-68) 0.50	4	1	2.55	NP	2.55	NP	1.63	NP
Shi 2015	HRT	Ever vs never (seq E+P)	0.18	50 (0-82) 0.11	4	2	2.70	NP	3.53	NP	1.75	0.80
Shi 2015	HRT	Ever vs never ET only	0.71	48 (0-74) 0.55	11	6	8.04	NP	8.28	NP	7.01	NP
Shi 2015	HRT	Ever vs never ET+PT	0.40	53 (0-76) 0.03	11	3	6.55	NP	5.60	NP	8.56	NP
Shi 2015	HRT	Ever vs never ET +E/PT	NA	NA	2	0	1.94	NP	1.94	NP	1.89	NP
Zhou 2008	HRT	Current vs ever	0.08	14 (0-69) 0.32	5	3	3.39	NP	3.61	NP	2.91	NP
Coll Grp OC 2008	OCP	Ever vs never	0.16	0 (0-37) 0.54	22	11	22.0	NP	22.9	NP	23.00	NP
Hankinson 1992	OCP	Ever vs never	0.41	13 (0-48) 0.28	22	1	1.58	NP	1.58	NP	0.73	0.71
Wen 2019	Metformin	Ever vs never	0.38	NA	3	2	2.41	NP	2.38	NP	2.42	NP
Baandrup 2013	NSAIDS Non aspirin	Ever vs never	0.55	0 (061) 0.93	16	0	1.02	NP	1.02	NP	1.02	NP
Liu 2014	Statin use	Any vs none	0.23	0 (0-64) 0.67	5	0	2.21	NP	2.21	NP	1.88	NP
Liu 2014	Statin use	Any vs none, longterm	0.31	0. (0-73) 0.78	3	1	2.08	NP	2.08	NP	2.04	NP
**Biochemical markers**												
Li 2017	CRP	Middle vs low	NA	NA	7	1	0.83	0.65	0.83	0.65	0.83	0.65
Li 2017	CRP	>10mg/L vs <10mg/L	0.152	13 (0-72) 0.33	7	2	4.00	NP	4.00	NP	3.85	NP
Li 2017	CRP	High vs low	0.088	61 (0-81) 0.00	7	4	2.26	0.16	3.64	0.78	3.68	0.81
**Reproductive factors**												
Wu 2019	Age at last birth	Highest vs lowest	0.58	74 (50-84) 0.31	13	5	7.65	0.14	9.05	0.15	2.37	0.06
Gong 2013	Age at menarche	Oldest vs youngest	0.87	42(0-63) 0.02	24	4	6.80	NP	6.93	NP	1.31	0.00
Li 2014	Breastfeeding	Ever vs Never	0.71	76 (68-82) <1E-100	55	24	3.07	0.01	3.13	0.01	3.85	<1E-100

Luan 2013	Breastfeeding	Per 5mo increase in duration	0.59	22 (0-78) 0.28	25	1	0.28	0.16	0.30	0.18	0.17	0.04
Ip 2007	Breastfeeding	>12mo vs never	0.30	71 (4-86) 0.00	6	2	3.36	NP	4.47	NP	0.74	0.12
Siristatidis 2013	IVF	Ever vs never (reference group general population; excl OC diag <1yr post rx)	0.64	65 (0-83) 0.01	6	2	1.96	NP	1.89	NP	1.55	NP
Siristatidis 2013	IVF	Ever vs never (reference group IVF population; total follow up)	0.91	23 (0-69) 0.26	6	1	2.22	NP	2.3	NP	1.64	0.74
Zhou 2017	PID	Ever vs never	0.35	NA	14	1	2.48	NP	2.76	NP	1.01	0.28
Negri 1991	Nulliparity	Yes vs no	0.87	0 (0-73) 0.85	3	1	0.95	0.95	0.95	0.95	0.89	0.89
Wang 2016	Tubal ligation	Ever vs never	0.78	86 (81-89) 0.00	25	16	20.9	NP	21.4	NP	14.3	0.50
**Carcinogens**												
Reid 2011	Asbestos[§]	Any vs none	0.64	28 (0 -61) 0.15	16	4	2.75	0.40	2.94	0.49	1.87	0.10
Camargo 2011	Asbestos; Occupational[§]	Total exposed vs nonexposed	0.72	35 (0-61) 0.06	20	6	2.30	0.01	2.47	0.02	1.06	<0.01
Camargo 2011	Asbestos; Occupational[§]	High exposed vs nonexposed	0.78	45 (0-77) 0.11	6	2	1.06	0.31	1.06	0.32	0.31	0.00
Berge 2017	Genital talc use	Ever vs never	0.70	39 (0-61) 0.02	27	13	14.5	NP	14.4	NP	17.2	NP
Penninkilampi 2018	Perineal talc use	Long term (>10yrs) vs none	0.30	45 (0-71) 0.04	12	4	2.44	0.26	2.62	0.33	1.05	0.53
**Physical activity**												
Zhong 2014	Recreational	Moderate vs low	0.59	45 (0-66) 0.02	20	4	3.25	0.65	3.70	0.86	4.07	0.97

Abbreviations:BMI, body mass index; cc, case control; CI, confidence interval; cm, centimetres; cont, continuous; CRP, C reactive protein; Dx, diagnosis; E+P, estrogen and progesterone; endo endometriosis; E/PT, combined estrogen and progesterone therapy; ET, estrogen/oestrogen therapy; excl, excluding; g, grams; HRT, hormone replacement therapy; IU, international units; IVF, in vitro fertilisaiton; inf, infertile; iya, in young adulthood; kg, kilograms; m2, metre squared; ml, millilitres; mo, months; ng, nanograms; nmol, nanomole; NSAIDS, non-steroidal anti-inflammatory drugs; OC, ovarian cancer; PCOS, polycystic ovarian syndrome; PID, pelvic inflammatory disease; PrMp, premenopausal; PoMp, postmenopausal; PT, progesterone therapy; ref, reference; RR, relative risk; RX, treatment; seq, sequential; ug, micrograms; WCRF, World Cancer Research Fund

[§]Risk of dying from ovarian cancer
